# Sustainability of locally driven centres for those affected by dementia: a protocol for the get real with meeting centres realist evaluation

**DOI:** 10.1136/bmjopen-2022-062697

**Published:** 2022-05-02

**Authors:** Thomas Morton, Shirley B Evans, Dawn Brooker, Tracey Williamson, Geoff Wong, Michela Tinelli, Faith Frost, Jennifer Bray, Nigel Hullah

**Affiliations:** 1 Association for Dementia Studies, University of Worcester, Worcester, UK; 2 Nuffield Department of Primary Care Health Sciences, Oxford University, Oxford, UK; 3 PSSRU, London School of Economics and Political Science, London, UK; 4 3 Nations Working Group for Dementia, Swansea, UK

**Keywords:** Dementia, Organisation of health services, SOCIAL MEDICINE, Old age psychiatry, Organisational development, QUALITATIVE RESEARCH

## Abstract

**Introduction:**

Improving support for people with early to moderate dementia to live at home in their communities is a global public health goal. Community adult social care is not robust in many parts of the UK, however, with the pandemic increasing pressure on services for this population. Community-led interventions can play a key role in supporting people postdiagnosis, helping delay decline, but many interventions struggle to sustain beyond 1–2 years. Meeting Centres (MCs) are one such intervention, which many UK community groups find attractive and achievable. However, it is not understood how these communities can ensure they are putting in place strategies that will help them sustain in the longer term, beyond start-up phase.

**Methods and analysis:**

This realist evaluation aims to understand the factors affecting sustainability of MCs in rural areas and learn lessons from MCs that have sustained beyond 3 years. Data will be collected using mixed methods: interviews and group discussions with stakeholders involved at every level in three case study locations in England and Wales, analysed with Soft Systems modelling; a Discrete Choice Experiment exploring what people across the UK value and are willing to pay for MCs, analysed with regression modelling. All data will be synthesised using a Realist logic of analysis to build a theoretical model of how, why, for whom, in what contexts and to what extent MCs can be successfully implemented for the long term.

**Ethics and dissemination:**

As participants may lack capacity for informed consent, favourable ethical opinion was received from a Health Research Authority research ethics committee. Resulting recommendations will be of interest to stakeholders including those commissioning, planning, running, supporting or attending MCs, as well as policy-makers and healthcare professionals. Knowledge will be shared with emerging MCs to help accelerate scale up of this intervention.

**Strengths and limitations of this study**

- A Realist approach is well suited to accommodate and account for the complexity of such ‘real life’ intervention programmes, as implemented under different conditions in different settings, to extract transferable conclusions.
- This study is designed to draw on and disseminate a wide range of knowledge and expertise from people with extensive first-hand experience in tackling the issues involved in keeping a community-led dementia intervention running long term.
- The Meeting Centre is an intervention type that is rapidly growing in popularity across the UK at this current time, hence this research well placed to offer timely practical insights to help multiple new community-led interventions of this type become established and avoid common pitfalls that may threaten sustainability.
- This study is designed to gather evidence for how to successfully sustain a Meeting Centre for people affected by dementia or similar community-led intervention in a rural UK context, not data on the effectiveness or otherwise of this particular intervention type.
- This research cannot guarantee solutions to all of the challenges to the sustainability of a community-led intervention such as a Meeting Centre; instead it may highlight some challenges that are insurmountable given current approaches and policy, regarding health and social care systems.

## Introduction

Supporting people living with dementia (and those that, in turn, care for or otherwise support them) to live as well as possible in their communities, with timely psychosocial support, is a global public health goal.^1^ However, support following a diagnosis of dementia is patchy across the UK,^2^ with people and families in some areas lacking any formal proactive support beyond occasional contact with primary care and third sector. With an ageing population^3^ and increasing pressure on already stretched health services^4^ policy has for some time pointed to the need to move towards a model of social care where more people are cared for and supported at home, in the community. Improving provision of early, postdiagnosis support, improving support for family/informal carers and improving support for integrated care (involving the voluntary and independent sectors)—all in a more dementia-friendly community environment—are contemporary UK Government priorities for dementia care.^2^

However, multiple prepandemic reports described a climate where the state of social care provision—mainly delivered piecemeal by private and third-sector organisations—is precarious and dysfunctional’ in many parts of the country^4^ and in some areas has ‘broken down’ creating 'care deserts'.^5^ There is an associated reliance on informal carers (eg, family members) to step in to meet the needs of loved ones, but there is a growing recognition that informal carers’ own health and well-being is often negatively impacted by their caring activities.^6^ The detrimental health impact of social isolation and loneliness is also increasingly being recognised,^7 8^ with survey data revealing 60% of people living with dementia report loneliness, isolation and losing touch with people in their lives since diagnosis, around a quarter feeling they are not part of their community and that people avoid them.^9^ Family carers can also be subject to such loneliness and isolation.^10^

There have been various attempts to mitigate these challenges in communities across the UK, in the form of groups and activities for people with dementia and family/informal carers. These aim to serve a number of functions. However, there are significant gaps in social care for people affected by dementia across the UK.^5 6 9^ Care systems are unprepared for the forecasted doubling of the number of people living with dementia (1.6 million) and tripling of social care costs by 2040.^11^ Scaling up provision of evidence-based community initiatives for people with dementia and those that support them is imperative.^12–18^ The benefits of community-based initiatives are now being recognised.^14–18^ However, they face a variety of challenges in sustaining long-term. These challenges and how to meet them are much talked about in the dementia care policy, rhetoric and practice arenas but have received very little research attention, as identified in the SCI-Dem review (2018–2020).^19 20^ This research also showed many community initiatives are not sustained beyond 1–2 years.

### Meeting centres

Meeting Centres (MCs) for people affected by dementia are a community initiative based on a successful Dutch model^12 13 21^ that have emerged in the UK since 2015. MCs are distinct from day care, supporting both people with dementia and those that support them (eg, children, partners and friends) together, and connecting people to each other and their community. They build on Dementia Friendly Communities and are a step up in support from Dementia Cafes. At their heart is a small social club (15 people per day plus supporting family, friends and carers), based in an ordinary community building, close-by to where people live. They typically operate up to three times per week, providing people the chance to build friendships, peer support, understand their problems, get help and prepare for the future. Evidence-based postdiagnostic interventions are also provided in MCs, geared to the needs of members and facilitated by a small team of staff and volunteers trained in person-centred dementia care and the Adaptation-Coping Model.^22^ Following substantial European research (MeetingDem: 2014–2017).^14 23–25^ MCs were recommended as a social care intervention for those affected by dementia^14^ and found to help people, their families and communities build resilience for the longer term.^12–15 26–32^ The first MC in the UK opened in 2015, and there are now more than 30 either running or with funding to open in 2022, with accelerated interested from communities around the UK since lockdown restrictions were eased in 2021.^33^

The focus is now turning from how to establish these interventions, to how to keep them going long-term, in the face of a challenging social care-funding climate. To date, early adopter MC sites have devised different strategies to mitigate against threats and circumstances affecting their successful continuation. A better understanding is needed of the issues MC stakeholders have faced and are likely to face, and what can be learnt from this to prevent ‘reinvention of the wheel’ and help ensure sustainability. This is particularly true in rural communities where people and families living with dementia face increased barriers to being able to access support, guidance and connection.^34^ No MC-focused studies have yet investigated the factors that are key for the sustaining MCs after the start-up stage, and there is little focusing on this aspect with regards to other community-based interventions broadly serving a similar function and demographic. If these kinds of interventions cannot survive long term, the gap in provision will remain and the situation can be expected to worsen significantly with the rise in numbers of people living with dementia needing support.

### Research question and overall aim

To understand the factors affecting the sustainability of MCs for people affected by dementia in rural areas, how these can best be tackled, and what lessons can be gleaned for emerging MCs.

### Objectives

1. To empirically test the theory developed by the SCI-Dem review regarding the factors involved in sustainability of a community intervention for people affected by dementia.
2. To produce an in-depth transferable understanding of the key factors that may threaten the long-term delivery of an MC in the form of a refined Realist programme theory.
3. To explore people’s willingness to pay for MC provision via a discrete choice experiment (DCE) with those who support people living with dementia, triangulated with the perspectives of people living with dementia.
4. From the programme theory and DCE above, to build a model of how best to design, implement and deliver an MC under different conditions so it has the best chance of sustaining long-term.
5. To develop evidence-informed guidance materials for those in practice and evidence-informed recommendations for use at commissioning and policy level.

## Methods and analysis

### Project overview

**Figure 1:**
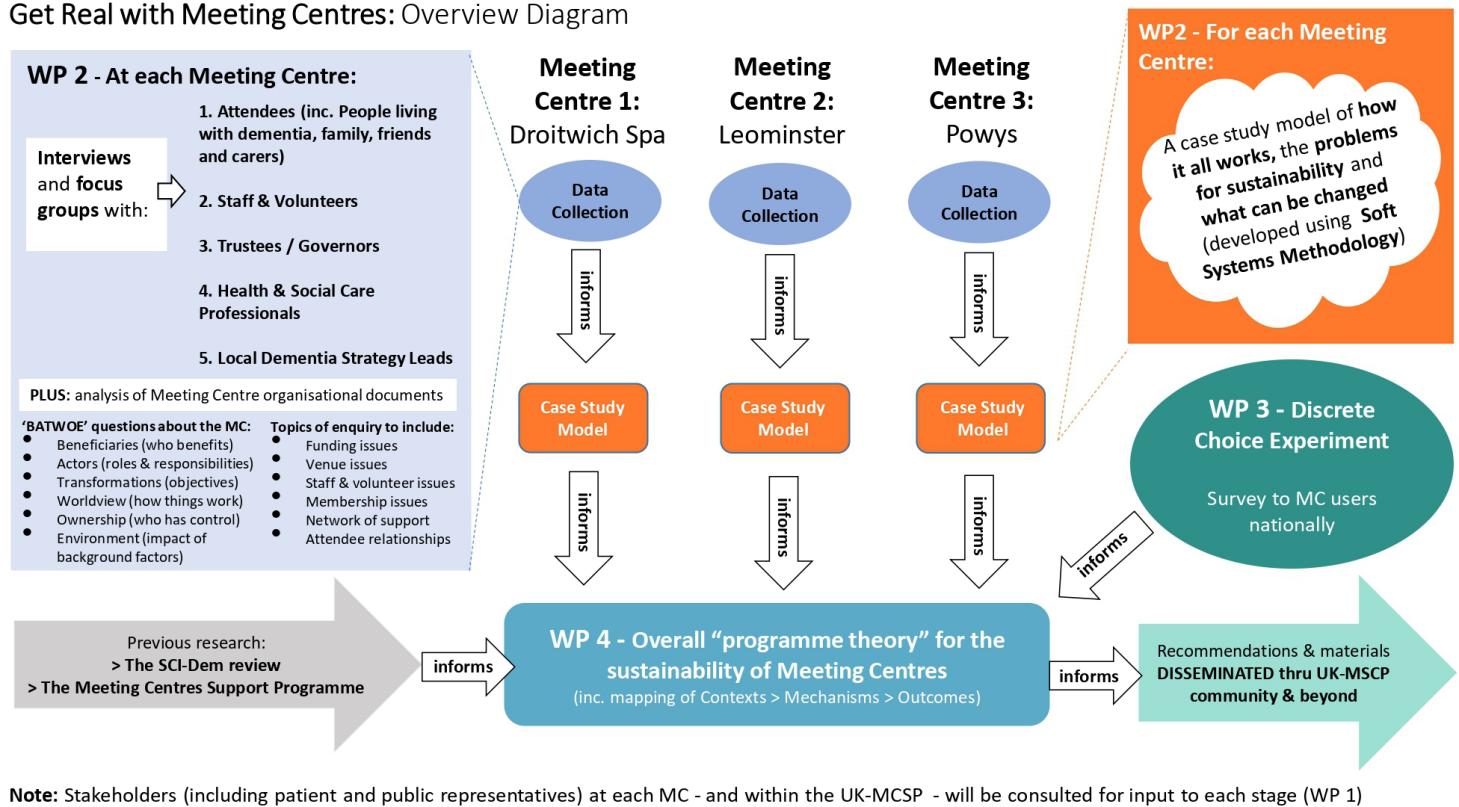
Overview of Get Real with Meeting Centres (MC) project. MCSP, Meeting Centre Support Programme.

This Realist Evaluation^35^ will comprise primary data collection from three MC research sites, that have each taken different approaches to serving different rural community settings, to investigate what works, under what circumstances, for whom, how and why, regarding configuring MCs for long-term sustainability. Data collection will comprise interviews and focus group discussions with participants in a range of roles relating to the MC under investigation, along with documentary and demographic data from each site. We will produce a case study model of each MC site with the aid of Soft Systems Methodology (SSM).^36^ These models will be synthesised and analysed using a Realist logic of analysis^35^ to generate theoretical causal chains of how contexts (background circumstances) can trigger mechanisms (responses and processes within people and organisations) to produce desired or undesired outcomes. We will also conduct a DCE survey regarding what activities and elements of MCs members most value and how much they are willing to pay for these, which will also feed into the overall Realist analysis. This work will be split across five work packages. Figure 1 shows an overview of the study framework.

The use of systems approaches to complement Realist thinking is an area of growing interest with multiple successful examples integrating the two in recent years.^37 38^ Both Realist and Systems approaches are appropriate for this research as they aim primarily to deal with, and understand, the complexity of systems with human actors in real-world settings. Social care interventions such as MCs tend to be especially complex as they can involve multiple agencies and are embedded in a wider community setting, often with informal and impermanent elements making up part of how they work. Realist approaches focus on explaining the causal mechanisms of action that underlie complex programmes or interventions, to explain why they may be successful in some instances but not in others.^39^ SSM^36^ describes a process of enquiry to uncover real-world complexity by consulting those involved with a programme, to build up a conceptual model and determine what action can be taken to change things. The approach is designed to tackle organisational problems where the exact nature of the problem may not be agreed on and need investigating. Hence, the issue of the sustainability of an MC programme, with all of the factors that could involve in a complex community-based setting, is a good fit for a combination of realist and systems approaches.

### Patient and public involvement

Patient and participant involvement (PPI) will be channelled through each MC research site, led by dedicated members of the project team including a lay coapplicant who is living with dementia. Prior to the start of this study, PPI was carried out with members of one MC and lay members of the UK Meeting Centre Support Programme (UK-MCSP) National Reference Group, which includes members of Dementia Engagement and Empowerment Project network^40^ and Together In Dementia Everyday carers’ network.^41^ Views were sought about the importance of the study, its design, focus, recruitment approaches, factors likely to affect participation, payment/other reward for PPI, dissemination, approaches to PPI in the study, support needs (eg, PPI training/development) and interest in further involvement if the study was funded. PPI representatives will be fully supported to input into data collection processes and materials, synthesis and interpretation of data, and the creation of recommendations and resources for dissemination, as well as channels of dissemination. We will also continue to work with the UK-MCSP National Reference Group, to provide steering input.

### Work package 1: stakeholder group engagement and enquiry

Iterative stakeholder consultation throughout a project is a standard part of Realist Evaluation.^42^ Stakeholders will include patient and public representatives (whose involvement is covered in the section above) as well as stakeholder professionals with experience of community dementia support. The latter will also be consulted regarding data collection processes and materials, synthesis and interpretation of data, creation of recommendations and resources for dissemination, and channels of dissemination. This work is organised as work package 1 as it is central to the progress of the evaluation.

### Work package 2: data gathering and MC case studies

We have identified three MCs in different rural communities that have been operational for over 3 years and that meet the Essential Features of Meeting Centres^43^: One in a small market town in Herefordshire (opened February 2016; rural; deprivation rank^44^ around MC: 3288 of 32 844 neighbourhoods in England); one in a larger market town in Worcestershire (opened September 2015; semi-rural; deprivation rank^44^: 17 429 of 32 844 neighbourhoods in England); One rural county in Mid Wales with four federated small town MCs run by the same organisation (opened March 2017; rural; deprivation rank^45^: areas ranging from 284 to 1687 of 1909 neighbourhoods in Wales). Study sites have been selected purposefully, as they have been able to continue operating for at least 2 years prior to the commencement of this study, notwithstanding some necessary pausing or alteration of activities during COVID-19 restrictions. In addition to different geographic and demographic factors, MCs at each site have taken their own individual approach to the implementation and delivery of the service.

Participants will likewise be selected purposefully for their role and involvement in each MC, and for the experience they might bring regarding issues outlined in the research question and objectives. The project team will work with MC leads to identify appropriate potential participants in each role and approach those in roles outside the MC itself. MC staff will approach MC attendees to invite them to participate in the study, help them to better understand the participant information and consent process to make an informed decision on taking part.

Interviews and focus groups will take place with those involved at every level, in the following manner per MC, as shown in table 1.

**Table 1:** Participants and methods of data collection

Role of participant in MC	Method of data collection	No of participants
MC attendees (people living with dementia)	Focus group and/or one-to-one interview (individual or supported by a partner)	6
MC attendees (people supporting someone with dementia)	Focus group and/or one-to-one interview	6
MC staff and volunteers	Focus group and/or one-to-one interview	6
Those involved with governance at each MC	One-to-one interview	4
Health/social care/third sector professionals involved in the local dementia care pathway	One-to-one interview	4
Other stakeholders involved in local dementia strategy, for example, Dementia Friendly Communities Programme	One-to-one interview	4
		**Total: 30**

MC, meeting centre.

We anticipate 30 participants per MC. This number should give us a range of perspectives per type of participant, at different levels, to draw on to create SSM conceptual models, while also being realistic in terms of numbers available to take part at each MC and practically manageable within the scope and timeframe of the study. Interviews and discussions are anticipated to take between 20 min and 1 hour and will be conducted on site at each MC where possible, though we anticipate a proportion will be conducted via virtual videoconferencing or telephone due to the impact of the COVID-19 or participant preference.

Interview schedules were developed and piloted with stakeholder input and can be found in online supplemental file 1. The development of these were guided in part by the factors involved in the sustainability of community-based interventions found in the SCI-Dem Realist Review^19 20^ (summarised in online supplemental file 2) but also a modified SSM ‘BATWOE’ structure as outline in table 2.

10.1136/bmjopen-2022-062697.supp1 Supplementary data

10.1136/bmjopen-2022-062697.supp2 Supplementary data

**Table 2:** The elements of the SSM ‘BATWOE’ structure^36 37^

**B**	Beneficiaries (who is the system aimed at helping, eg, people living with dementia and those that support them)
**A**	Actors (people’s roles and functions in the system, for example, staff, volunteers, governors, referrers, social care professionals, community stakeholders)
**T**	Transformations (ie, going from start-up MC to established MC to stable and thriving MC)
**W**	Worldview (eg, how do things work regarding sustainability, what are the challenges and what should be done?)
**O**	Ownerships (ie, who or what can influence or thwart success of an MC)
**E**	Environment (ie, what are the background contextual factors that could boosts or constrain success?)

MC, meeting centre; SSM, Soft Systems Methodology.

The content of interview and discussion transcripts pertaining to the sustainability of MCs will be extracted and categorised by theme (generated both deductively and inductively) using NVivo qualitative data analysis software.^46^ Two researchers will undertake this and independently theme and categorise 10% of each other’s workload and compare and discuss any discrepancies for standardisation purposes. Any remaining disagreements will be discussed with the whole research team, as will the final list of data categories. Data will then be analysed using SSM procedures,^36^ applying a sequence of steps to the data from each MC site to build a conceptual model of how things work which will be returned to participants to review. This will act as the basis for developing a Realist programme theory with all data combined (see work package 4)

### Work package 3: a DCE to measure people’s willingness to pay for successful MCs

Successful implementation of evidence-based services in health and social care depends largely on the fit of the services with the values and priorities of stakeholders who are shaping and participating in their delivery and use.^47^ A flexible health economics tool for measuring choices in health and social care-related settings is the DCE,^48^ which measures preferences from individual decision-makers over alternative scenarios (or service provisions). Each alternative is described by several attributes (or characteristics) and the choices made between two or more competing scenarios subsequently determine how preferences are influenced by each attribute (eg, which attributes are valued as well as their relative importance). It can also provide a measure of the overall value attached to different alternatives (and identify optimal service provision that meets stakeholder requirements and have the best chance of sustainability in the long term). When a cost attribute is included, the DCE technique can also allow weighing of the benefits and costs of service provisions and calculating: how much stakeholders may be willing to pay for a particular service provision and measure how their willingness to pay may vary from current provision to their preferred option. Hence a DCE survey will be developed to measure people’s preferences for what can MC provides in terms of types of activities, social opportunities and emotional support, as well as the frequency of meetings, their availability and costs.

The DCE follows steps as laid out in the standard guidelines.^49^ The attributes (characteristics) and their various levels will be informed by the essential features of an MC^43^ and the Adaptation-Coping Model,^22^ as well as data from our three case study sites regarding the typical range of cost to members and days/time open or available per week. Qualitative data regarding what members value about MCs, from early work package 2 interviews, will be then used to validate and refine the attributes and levels. PPI stakeholders will be also consulted on the development of the presentation, wording and format of the survey. Due to the cognitive load involved in completing the survey, our target group will be people who support/care for an attendee who is living with dementia, reporting preferences on their behalf. Triangulation focus groups (n=3–6 per MC site) will be conducted with people living with dementia at each MC to ensure the views of this population are not excluded. The questionnaire will be distributed via staff at all UK MCs, as either an online survey or paper copy, depending on preference, with the aim of securing responses from more than 300 people.^49 50^ Efficient experimental design techniques will be applied to create the DCE choice set^51^ and data will be modelled using logit techniques (NLOGIT V.6 software).

### Work package 4: realist theory refinement and development of materials for practice

Data from all work packages, categorised and organised as themes, will be further analysed using the same Realist logic of analysis^35^ as the SCI-Dem Realist Review,^19 20^ to develop a Realist programme theory. This second stage of analysis is needed to understand causation—that is, how differing contexts in different MCs trigger different mechanisms (the hidden causal processes within people and organisations) to cause desired or undesired outcomes. This will test the programme theory produced during the SCI-Dem review, and act as a basis for developing recommendations and materials that explain how to best implement community-based interventions to sustain past the start-up phase, in a variety of settings. As it is developed, this programme theory will be presented back to stakeholders in each MC (see Work Package 1) for feedback and advice that will be used to further validate and refine it. We will use this understanding to develop (among other things) tips on best practice, what pitfalls to avoid and what challenges may need to be planned for, grounded in the experiences and models of working of those involved with the three MCs at every level. Materials to disseminate this learning to those in practice, and at a commission level, will be developed in collaboration with stakeholders.

### Work package 5: investigation of who MCs do not reach or benefit

In December 2021, the NIHR approved an expression of interest to add a further work package to the Get Real study. This work package will investigate who is not being reached by MC support and why, in the following ways: A comparison of demographic data regarding who attends each case study MC and whether they are representative of the population of their communities (and if there are any demographic groups clearly under-represented); additional interviews with existing stakeholders involved in the MC referral process specifically regarding why referrals might not be made or declined; identification, recruitment and additional interviews with dyad pairs (n=10) who have been made are aware of an MC but have decided not to attend or stopped attending, to understand what happened and why. This will again feed into Work Package 4.

## Ethics and dissemination

Favourable ethical opinion for the whole study was received from a Health Research Authority (HRA) Research Ethics Committee (REC) prior to starting recruitment and data collection. This was a requirement of the National Institute for Health and Care Research (NIHR) funders and in any case deemed necessary because some participants may lack the capacity to provide informed consent, or their ability to consent may change over time. Under the terms of the Mental Capacity Act 2005,^52^ people who lack the capacity to consent cannot be included in research unless the research concerns their condition. This research is concerned with improving the implementation and sustainability of social care interventions related to the condition of dementia, in order to improve the provision of support for the benefit of people living with dementia and those who support them. Favourable ethical opinion was given by Wales REC4 (21/WA/0185).

### Consent and risk of distress

**Figure 2:**
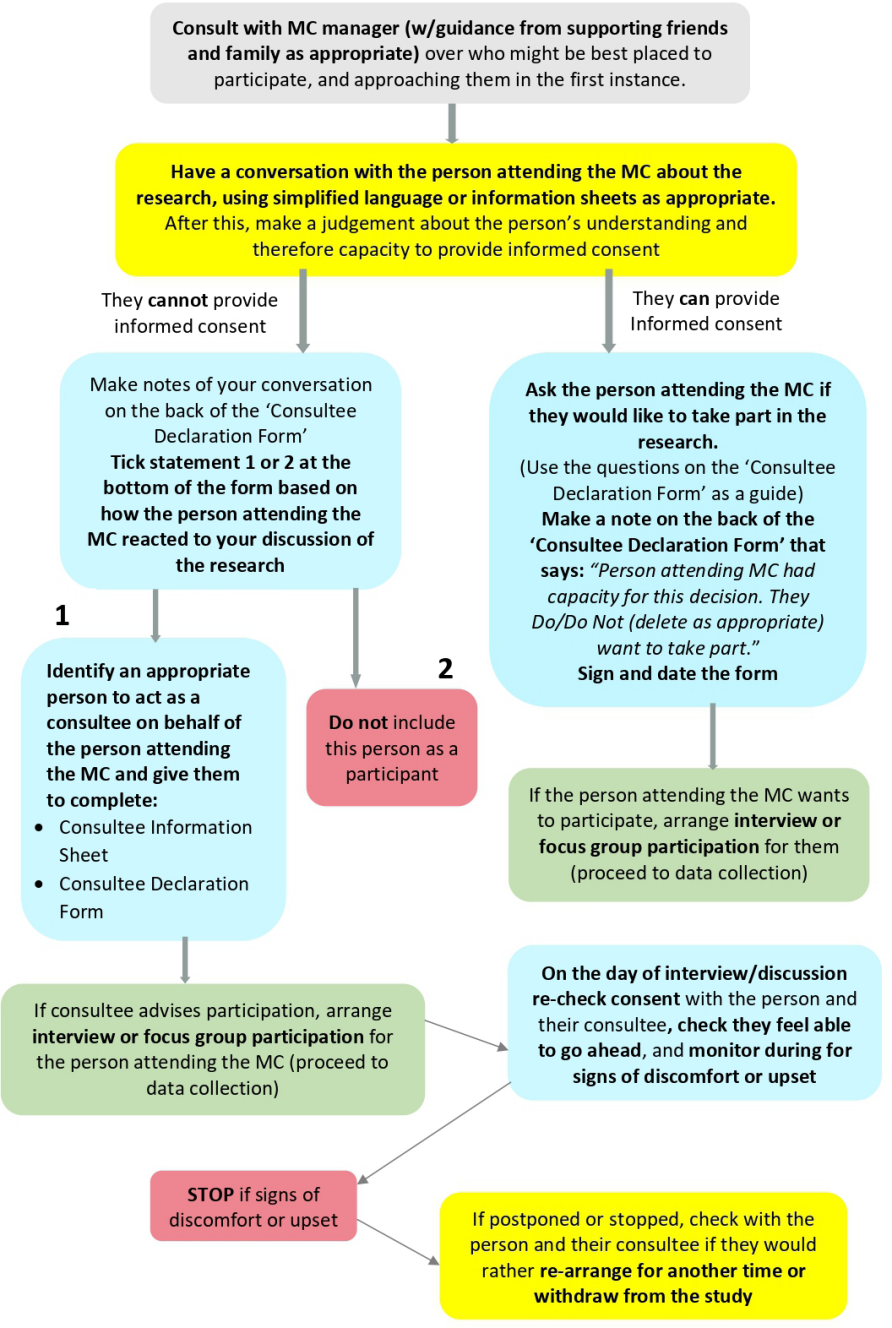
Consent process flow diagram. MC, meeting centre.

Participant information (see online supplemental file 3) and consent documents (see online supplemental file 4) were developed for a range of possible participants in line with HRA guidance.^53^ It was felt important to include MC attendees in the research for two reasons: (1) to ensure the perspectives of the people MCs are designed for are fully and authentically represented in line with a ‘Nothing about us, without us’ ethos; (2) to access key knowledge and experience, because attendees are in a position to offer key first-hand perspectives not directly available to others such as staff and governors, particularly regarding the factors that can encourage or act as a barrier to engaging with and attending a local MC. In order to undertake research ethically with these participants, the research team developed sensitive and relevant practices of informing and negotiating consent to participate. Figure 2 outlines the process of determining ability or provide informed consent:

10.1136/bmjopen-2022-062697.supp3 Supplementary data

10.1136/bmjopen-2022-062697.supp4 Supplementary data

The focus of the interviews, group discussions and questionnaire questions will be the MC, people’s experiences and opinions of it and preferences regarding what it provides. This focus is not expected to include topics that might be overly personal, sensitive, embarrassing or upsetting. Nevertheless, there is a risk semistructured interviews and discussion may stray into personal or sensitive areas that participants may not be comfortable with. For this reason, question topics will be explained to participants (and their consultee if they have one) before and interview or group discussion. Participants will also be told they do not have to answer any question they are not comfortable with and that they can stop the interview or leave the discussion at any time. The researchers will also be on alert for any signs of distress and will pause proceedings if a participant shows signs of discomfort or upset, or of reluctance to take part, at any time before or during interview or group discussion. The participant (and their consultee if they have one) will be asked if they are happy to continue, would prefer to rearrange for another time, or withdraw altogether. If there is any sign of discomfort with a sensitive or personal topic that is not necessary to discuss, researchers will automatically move the conversation on to a topic that is not personal or sensitive. Participants who are attendees of the MC will undertake interviews and discussions at the MC itself, with trained staff on hand to help if they do become distressed or upset. Questions will also be framed in terms of how to overcome challenges for success, and what can be learnt to help success in the future, and piloted to ensure there is no suggestion that our research means a MC’s future is under threat or that blame for any perceived failings in running it is being sought, which could be upsetting for participants.

The DCE questionnaire will be anonymous and only asks for opinions and preferences, hence there are not anticipated to be any risks in taking part. Participant information explaining the nature and objectives of the questionnaire, and consent questions, will be presented as part of the questionnaire itself. We will also hold focus group discussions with participants living with dementia in our MC case study sites, on the same questions covered in the DCE questionnaire, to triangulate, with an identical consent process to the other interviews and group discussions.

Regarding COVID-19 risks, we will only undertake face-to-face data collection if local and national guidance allows visitors to MCs and the MC and potential participants feel safe to do so. This situation will be continually monitored. Where face-to-face data collection is not possible or agreed, we will move to online data collection supplemented by telephone calls where preferred.

### Data management and confidentiality

All data gathered that may identify participants will be kept in password protected files and folders on the University of Worcester’s secure cloud-based storage, with unique participant identification codes used in data storage, known only to the research team. This includes interview and discussion recordings and transcripts, consent documentation and any personal data collected to maintain contact with participants. Where physical copies of data are necessary, they will be kept in locked cabinets on University premises. Face-to-face meetings will be recorded digitally on an encrypted recording device, online meetings using facilities provided by that online platform, with recordings transferred at the first opportunity and deleted once transcribed by a member of the research team or a trusted external transcription service and checked for accuracy.

Transcripts will be anonymised through the removal of names and other personal information. However, it should be noted that for the sake of analysis some information may be necessary to retain on a participant’s role within the system of each MC, or information specific to the local context, which may make jigsaw identification possible. Hence participants will be alerted to this when taking consent and their preferences on anonymity and identification will be gathered and checked before reporting. In reporting, MCs will not be identified specifically by name or town, but only by region and pertinent demographic factors.

### Dissemination

The UK-MCSP,^54^ led by the University of Worcester, has created a UK-wide Community of Learning and Practice comprising 300 organisations involved or interested in providing MCs in their communities, with more than 30 MCs now on-stream and more planned across the country. There is also a National Reference Group comprising 30 national organisations drawn from policy and practice. This networked community of stakeholders will be consulted to help co-create accessible resources and disseminate them according to their preferences, to ensure knowledge from this research is accessible to those involved in the day-to-day governance, management and running of MCs in the UK. This will also involve workshop activities with PPI representatives/stakeholders within the case study MCs. Dissemination will take place through these MC network channels, as well as to a wider audience through practitioner workshops, webinars, blogs, newsletters and social media. Learning form this study will also be incorporated into training for emerging and existing MC personnel provided by the University of Worcester.

In addition to academic publications and conference presentations, outputs will include an accessible publication and website downloads for a non-academic audience that will detail the three case studies and overall analysis, useful for all in similar community settings looking for a flexible template that they might implement; there will also be published evidence-based guidelines for commissioners and providers of community-based interventions for people affected by dementia. Specifically, Worcestershire County Council will use knowledge from this research to support new MCs in the county. A publicly accessible report summary will be available on University of Worcester Association for Dementia Studies website^55^ poststudy and findings will be promoted widely at MCs to reach study participants via posters, presentations and leaflets. A full study report will be made available on request.

## Supplementary Material

Reviewer comments

Author's manuscript
