# Supplementary material for: Sustainability of locally driven centres for those affected by dementia: a protocol for the get real with meeting centres realist evaluation
Source: BMJ Open. 2022 May 2;12(5):e062697. doi: 10.1136/bmjopen-2022-062697 (PMC9062872; doi:10.1136/bmjopen-2022-062697)
Supplement: Supplementary data [file bmjopen-2022-062697supp001.pdf]

Get Real with Meeting Centres

Interview/Group Discussion Schedule

For Meeting Centre personnel

Note: Which questions will be asked will be tailored to whether the interviewee has a strategic role or an operational role, in order to keep the number of questions concise and relevant, appropriate and understandable.

Introductions

Introduction of research staff and explanation of purpose:

"The purpose of this interview is to better understand how Meeting Centres have got up and running and how they have kept going over the past few years. We want to ask people for their knowledge and experience of how their Meeting Centre is run and what has made it a success, so that we can learn from it.

We will use what we learn to develop user-friendly guidance and materials to best help Meeting Centres survive and thrive in the long-term. We will also use this information to help policy makers and government understand what they can do to improve this situation. Our project will support the growth of more Meeting Centres to support people in desperate need. Thank you for agreeing to assist with this."

- Check provision of relevant study information sheet
- Confirm informed consent

Clarification of role:

- Ask for a description of the participant's role
- What do you do at the Meeting Centre? What are you responsible for?
- How did you get involved with the Meeting Centre?
- How long have you been involved with the Meeting Centre and has your role changed over time?

IRAS ID: 294636   Version: v0.3   09/06/21

Get Real with Meeting Centres

1: Questions about who the Meeting Centre is aimed at helping:

- Who can become a member and who else benefits?
- How do you assess if a Meeting Centre is appropriate for someone?
- How do you find people? Do they come to you or do you approach them?
- What PR is done?
- What makes people want to come along and keeps people coming back?
- What can put people off coming or lead people to leave?
- Whose needs might not be met and why?
- To what extent do people supporting other attendees (e.g. friends/family) attend themselves?
- Do friends and family feel they also benefit? Are there challenges for them (e.g. arranging transport, fit with working hours)?
- Are all attendees happy with the programme of activities? What is most enjoyed and what could be better?
- How often do you reflect/consult on what is being provided and how well it meets the changing needs of attendees over time?

2: Questions about people's roles at the Meeting Centre:

- Who is responsible for what regarding the planning and running for the Meeting Centre?
- How many people are there in each of the different roles and is that the preferred number?
- What is their background and how did they come to be involved/how were they recruited?
- Have any left those roles any why?
- What training have personnel had and how did that happen?
- Are attendees involved in decision making?
- Are there any external people (e.g. referrers, community stakeholders) who play a key role in supporting the Meeting Centre? How did they come to be involved?
- Are there any external people (e.g. care professionals, practitioners, people who deliver a service) who come into the Meeting Centre?
- Are there any challenges or points of tension in working with external people?

3: Questions about how a Meeting Centre has developed:

- What were the most important things to have in place when setting up? What helped or hindered these things?
- What are the most important things to have in place now, and going forward? Are these the same things, or is the focus different now?
- How has the Meeting Centre and its work changed over time?
- What and links to the broader community have been developed, and how?
- How did the Meeting Centre respond to the COVID-19 pandemic? How has that affected how it might function in the future?

IRAS ID: 294636   Version: v0.3   09/06/21

Get Real with Meeting Centres

4: Questions about how well things work.

- What things do you think are most key to keeping a Meeting Centre going long term?
- What are the key challenges in keeping going long term?
- What is good or not so good about how the Meeting Centre is run and its current situation?
- What opportunities are there for things to change?

5: Questions about who has the power to influence the success of a Meeting Centre, including:

- Whose support (person or organization) is essential to making a success of the Meeting Centre?
- What could influence their attitudes and decisions regarding the Meeting Centre?
- How could local or national authorities help or hinder the success of the Meeting Centre?
- Who in the Meeting Centre team could the Meeting Centre not continue without?
- How does the Meeting Centre meet its costs? What are the various sources of funding?
- How sustainable are these sources of funding? How difficult are they to come by?

6: Questions about the place and background circumstances around the Meeting Centre, including:

- Is the venue appropriate and working well for attendees and personnel?
- How does the local healthcare pathway fit with the Meeting Centre? Does it help, hinder or have no bearing on the Meeting Centre's success?
- How does the local/wider community view the Meeting Centre? Are there any misconceptions? Does it help, hinder or have no bearing on the Meeting Centre's success?
- How does the geography of the area (e.g. rural or urban) affect the Meeting Centre's success?
- How do people get to the Meeting Centre? What distance do they come from?
- How suitable is public transport? How available is community transport?

Interview closure

Is there anything else you'd like to tell us that you feel we should have asked you?

Many thanks for your time.

(Confirm any arrangements for checking accuracy of interpretation of views).

IRAS ID: 294636 Version: v0.3 09/06/21

Get Real with Meeting Centres

Interview/Group Discussion Schedule

For people who attend Meeting Centres

Note: Which questions are asked will be tailored to whether the interviewee is living with dementia or supporting someone who is, or if people are being interviewed as a pair, to ensure the questions are appropriate, understandable and kept concise and relevant. Concrete examples of people and organizations known to the participant will be used where possible, to make the questions less abstract.

Introductions

Introduction of research staff and explanation of purpose:

"The purpose of this interview is to better understand how Meeting Centres have got up and running and how they have kept going over the past few years. We want to ask people for their knowledge and experience of how their Meeting Centre is run and what has made it a success, so that we can learn from it.

We will use what we learn to develop user-friendly guidance and materials to best help Meeting Centres survive and thrive in the long-term. We will also use this information to help policy makers and government understand what they can do to improve this situation. Our project will support the growth of more Meeting Centres to support people in desperate need. Thank you for agreeing to assist with this."

- Check provision of relevant study information sheet
- Confirm who the participants are (and their relationship to each other if in a pair or group)
- Confirm informed consent or consultee declaration

IRAS ID: 294636   Version: v0.3   09/06/21

Get Real with Meeting Centres

1: Questions about who the Meeting Centre is aimed at helping:

- What do you like about attending the Meeting Centre?
- How did you find out about the Meeting Centre?
- What might put people off coming to the Meeting Centre or want to stop coming along?
- What do you think of the activities at the Meeting Centre?
- Is there anything that make it difficult or challenging to attend?
- How do you feel about the cost?

2: Questions about people's roles at the Meeting Centre:

- Do you have a role in choosing what activities take place and deciding things?
- Do you have a role in helping out around the Meeting Centre?
- How involved in the Meeting Centre is your GP, or any care workers, nurses or other health professionals you know?
- Who provides transport to get there?

3: Questions about how a Meeting Centre has developed:

- How has the Meeting Centre changed over time?
- What happened with the Meeting Centre during lockdown and how did you feel about it?

4: Questions about how well things work:

- What do you like most and what is not so good about the Meeting Centre?
- What is most important to the Meeting Centre's success, and what can get in the way?

IRAS ID: 294636 Version: v0.3 09/06/21

5: Questions about who has the power to influence the success of a Meeting Centre:

- Do you know if authorities like the County Council, the NHS, the Government or any relevant big national charities help or hinder the success of the Meeting Centre?
- Do you feel like you are in control of what happens at the Meeting Centre?

6: Questions about the place and background circumstances around the Meeting Centre:

- Is the venue appropriate and easy to get around?
- Do you know how your GPs, care workers etc. view the Meeting Centre?
- Do you know how the local community views the Meeting Centre?
- How easy is it for you to get to the Meeting Centre and what is transport like?

Interview closure

Is there anything else you'd like to tell us that you feel we should have asked you?

Many thanks for your time.

(Confirm any arrangements for checking accuracy of interpretation of views).

Get Real with Meeting Centres

Interview/Group Discussion Schedule

For external partners & stakeholders to Meeting Centres

Note: Which questions will be asked will be tailored to suit the relationship the external interviewee has with the Meeting Centre – e.g. whether a direct referrer or someone otherwise involved with the local dementia care pathway – to keep the number of questions concise and relevant, appropriate and understandable.

Introductions

Introduction of research staff and explanation of purpose:

"The purpose of this interview is to better understand how Meeting Centres have got up and running and how they have kept going over the past few years. We want to ask people for their knowledge and experience of how their Meeting Centre is run and what has made it a success, so that we can learn from it.

We will use what we learn to develop user-friendly guidance and materials to best help Meeting Centres survive and thrive in the long-term. We will also use this information to help policy makers and government understand what they can do to improve this situation. Our project will support the growth of more Meeting Centres to support people in desperate need. Thank you for agreeing to assist with this."

- Check provision of relevant study information sheet
- Confirm informed consent
- Ask for a description of the participant's role and involvement with the Meeting Centre

IRAS ID: 294636 Version: v0.3 09/06/21

Get Real with Meeting Centres

1: Questions about who the Meeting Centre is aimed at helping:

- What is your understanding of who can become a member and who else benefits?
- Do you have access to Meeting Centre advertising/info materials? Are they effective?
- How does the Meeting Centre reach the right people?
- If you are involved in referring, how do you assess if a Meeting Centre is appropriate for someone? What level of consultation is carried out with people and families?
- What factors do you think most make Meeting Centres attractive to people? What do people value most?
- What could put people off going to a Meeting Centre?
- To what extent do you think the Meeting Centre meets people's needs?
- What are the main benefits or challenges for the people supporting attendees (such as family carers)?

2: Questions about people's roles at the Meeting Centre:

- What role do you see yourself as playing in the Meeting Centre and how it functions?
- How did you get involved? Has your involvement changed over time?
- How important is the Meeting Centre, or initiatives like it, to your work?
- How important do you think your support is to the Meeting Centre?
- Who else (people or organizations) play a role in supporting the Meeting Centre? What do they do and how did they come to be involved?
- Are there any challenges or points of tension in working with the Meeting Centre?

3: Questions about how a Meeting Centre has developed:

- What was most important in helping or hindering the setting up of the Meeting Centre?
- What are the most important things to have in place now, and going forward? Are these the same things, or is the focus different now?
- How has the Meeting Centre and its work changed over time?
- Did you continue to have involvement with the Meeting Centre during lockdown? If so, how?
- How has the pandemic impacted upon the Meeting Centre, now and going forward?

4: Questions about how well things work.

- What do you think is most key to keeping a Meeting Centre going long term? What are the main challenges?
- What is good about how the Meeting Centre is run and its current situation?

IRAS ID: 294636 Version: v0.3 09/06/21

- What is not so good about how the Meeting Centre is run and its current situation?
- What opportunities are there for things to change – what should be done and what can be done?

5: Questions about who has the power to influence the success of a Meeting Centre, including:

- Whose support (person or organization) is essential to making a success of the Meeting Centre?
- Does anyone (person or organization) have the power to stop the Meeting Centre from being able to run?
- What could influence their attitudes and decisions regarding the Meeting Centre?
- How could local authorities help or hinder the success of the Meeting Centre? How could national authorities help?
- Who in the Meeting Centre team could the Meeting Centre not continue without?

6: Questions about the place and background circumstances around the Meeting Centre, including:

- Do you know if the venue is appropriate and working well for everyone?
- How does the local healthcare pathway fit with the Meeting Centre? Does it help, hinder or have no bearing on the Meeting Centre's success? How could things be done differently?
- How does the local/wider community view the Meeting Centre? Does it help, hinder or have no bearing on the Meeting Centre's success?
- How does the geography of the area (e.g. rural or urban) affect the Meeting Centre's success?
- How do people get to the Meeting Centre? What distance do they come from?
- How suitable is public transport? How available is community transport?

Interview closure

Is there anything else you'd like to tell us that you feel we should have asked you?

Many thanks for your time.

(Confirm any arrangements for checking accuracy of interpretation of views).

Morton T, *et al. BMJ Open* 2022; 12:e062697. doi: 10.1136/bmjopen-2022-062697
