# Supplementary material for: Sustainability of locally driven centres for those affected by dementia: a protocol for the get real with meeting centres realist evaluation
Source: BMJ Open. 2022 May 2;12(5):e062697. doi: 10.1136/bmjopen-2022-062697 (PMC9062872; doi:10.1136/bmjopen-2022-062697)
Supplement: Supplementary data [file bmjopen-2022-062697supp002.pdf]

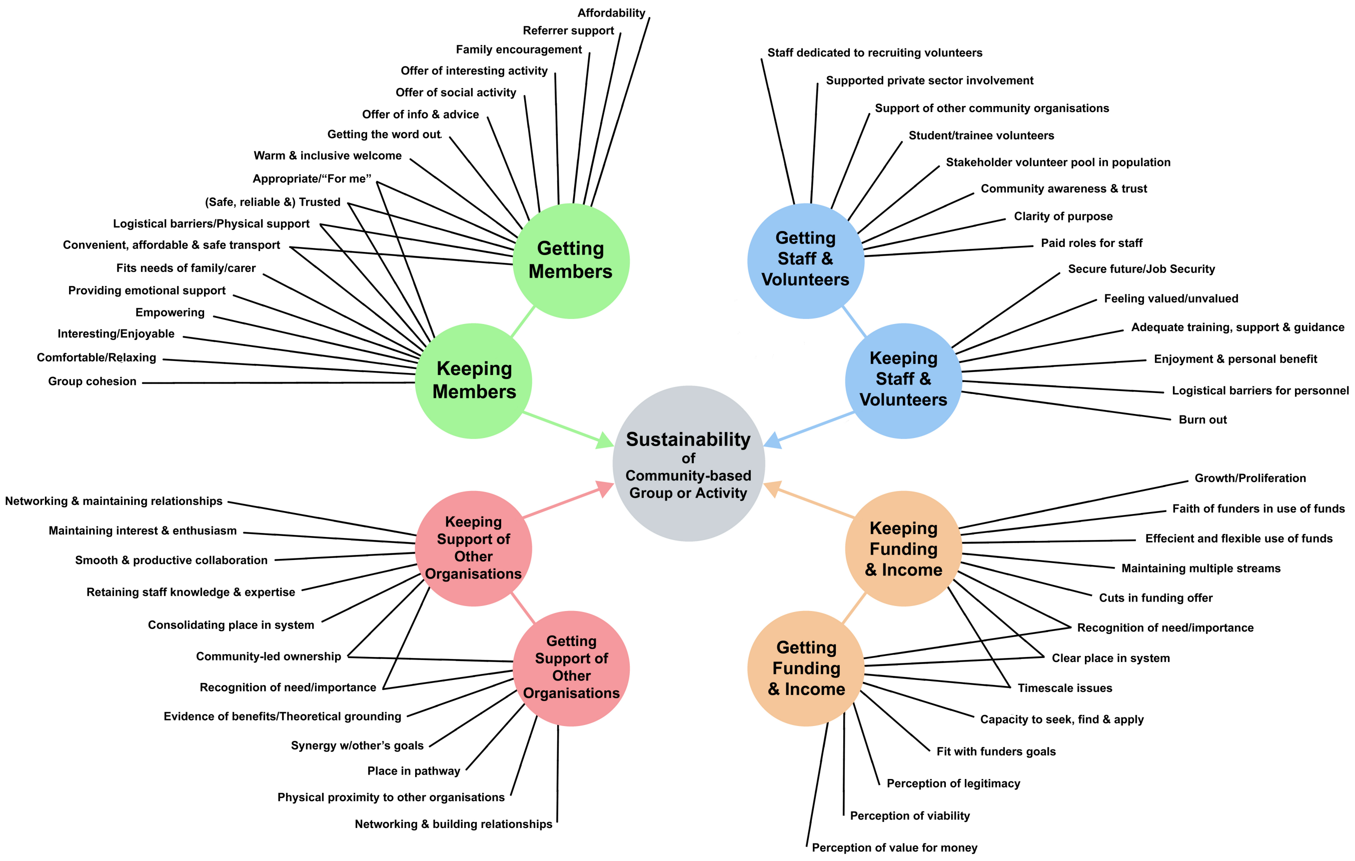

SCI-Dem overview: Factors affecting the sustainability of community-based groups and activities
