# Supplementary material for: Sustainability of locally driven centres for those affected by dementia: a protocol for the get real with meeting centres realist evaluation
Source: BMJ Open. 2022 May 2;12(5):e062697. doi: 10.1136/bmjopen-2022-062697 (PMC9062872; doi:10.1136/bmjopen-2022-062697)
Supplement: Supplementary data [file bmjopen-2022-062697supp003.pdf]

Information Sheet for people involved in Meeting Centres

Research Study Title - Get Real with Meeting Centres: A Realist Evaluation

You have received this information sheet because we would like you to take part in our research study. The following information explains what the research study is and what it would mean for you if you decide to be involved. Please take time to read over it and discuss the information with other people before you make your decision.

What is the purpose of the study?

This study will look at how three Meeting Centres have got up and running and how they have kept going over the past few years. One of these is the Meeting Centre that you have involvement with. Many more Meeting Centres are now starting, so it is important we learn more about what has helped to make Meeting Centres successful. We have developed this study so that we can learn from it and develop tips and guidance for others. We will take all the information people tell us and map out how each Meeting Centre works, how it has worked over the past few years, and how best to plan for the future, going forward.

Why am I being asked to take part?

We are asking you to take part as you have direct experience of being involved in a Meeting Centre. We want to ask people for their knowledge and experience of how their Meeting Centre is run and what has made it a success.

Do I have to take part?

No, you do not have to take part in the study if you do not want to. You will be given a week to decide. If you decide against it, it will have no impact on your involvement with your Meeting Centre. You can change your mind about being involved in the study at any time and without giving a reason. Information about people who have not consented to be involved will not be recorded as part of the study. You also have the right to withdraw your data after participation, in which case it will be destroyed on request.

Morton T, *et al. BMJ Open* 2022; 12:e062697. doi: 10.1136/bmjopen-2022-062697

If you decide that you would like to take part in this research study, you will first be asked to sign a consent form and then we will arrange with you the next steps of taking part.

What will happen if I wish to take part?

You will have the opportunity to be involved in informal interviews and group discussions over the coming months. You can make the decision on how much involvement you would like to have and which aspects you would like to get involved with.

The research study and COVID

We hope to be able to hold interviews and group discussions in person, but this is dependent on the COVID-19 restrictions. We are hopeful that in the near future it will be safe for us to visit each Meeting Centre. If so, we will abide by all current local and national guidance on what is safe to do when visiting. If this is not possible, then we will carry out our interviews and discussion groups by online meetings (such as via Zoom) or by telephone and email.

Even if we can attend Meeting Centres, you can let us know whether you would like to be involved face-to-face or via an online method such as Zoom.

Will my involvement be kept confidential?

We keep the specific feedback in individual interviews confidential, but we will state our general findings in our final report. However, if something is disclosed during interviews or discussions that could impact upon the safety of yourself or others, confidentially may be breached if necessary to ensure safeguarding. Any feedback that we get from you will be checked for accuracy and anonymised through the removal of people's names and other personal information. When we write up our findings, we will use quotes from people we have spoken with. You will be asked your preferences on whether or not you would like your name to be referenced by the quote. When the results of the study are presented, we will not use the name of the Meeting Centres involved in the study but will refer to them by region.

What are the possible disadvantages to taking part?

There are no obvious disadvantages to you taking part in the research. We are interested to hear your feedback.

What are the possible benefits of taking part?

By taking part in this study you will be providing valuable information that we can learn from, to help other Meeting Centres to be a success. You get to share your opinions on Meeting Centres, what you think of them and how they might improve to help those who attend them. You may also help your own Meeting Centre, by helping us to understand how it works and what else we can do to help it in the future.

We hope that you will find taking part in interviews and discussions with us interesting and enjoyable.

What will happen to the results of the study?

The results will give us the information to make recommendations to other Meeting Centres across the UK. The results of the study will be written up in a final report and shared with the National Institute of Health Research, who are funding the study. The results will also be used to write articles, reports or include in presentations. We will make sure our results are made available to share with you once the study is complete.

Who is organising or funding the study?

This study is being led by the Association for Dementia Studies at the University of Worcester. It is funded by the **National Institute for Health Research (NIHR)**

Who has reviewed the study?

This research study has been reviewed and approved by XXX. A research ethics committee is a group of people who review research studies to ensure they protect the dignity, rights, safety and well-being of research participants and researchers.

For any further information please contact:

Shirley Evans – PI

s.evans@worc.ac.uk

Association for Dementia Studies

University of Worcester

Henwick Grove, Worcester, WR2 6AJ

Thomas Morton – Research Associate

t.morton@worc.ac.uk

Association for Dementia Studies

University of Worcester

Henwick Grove, Worcester, WR2 6AJ

Thank you for taking time to read this information sheet.

Morton T, *et al. BMJ Open* 2022; 12:e062697. doi: 10.1136/bmjopen-2022-062697

Information Sheet (short version)

The Get Real with Meeting Centres Study

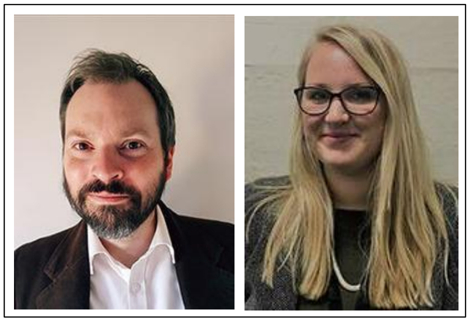

The Researchers:
Thomas (left) and Faith (Right)

What we are doing

We are doing a research study at the Meeting Centre that you go to because we want to find out more about Meeting Centres and what makes them successful. Meeting Centres are really popular and there are lots of new ones that are being set up. We hope that we can find out how to make them the best that they can be.

We would like you to talk to us about the Meeting Centre that you go to and your feelings about it.

How you can take part

Tell someone at your Meeting Centre if you would like to take part. You will be invited to take part in things like **group discussions** and **one-to-one conversations,** to talk to us about the Meeting Centre you go to.

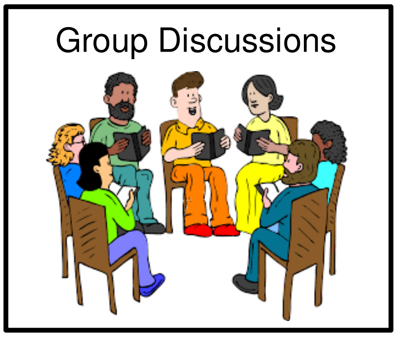

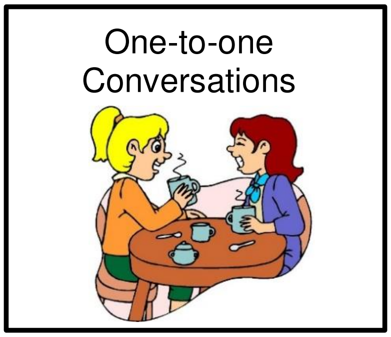

Morton T, *et al. BMJ Open* 2022; 12:e062697. doi: 10.1136/bmjopen-2022-062697

What will happen next?

We will ask you to sign a consent form saying that you want to take part. We will then arrange the next steps, like when you would like to talk with us and how.

Will my information be confidential?

The information you tell us will be checked for accuracy and anonymised by removing anyone's names and any other personal information. If we quote something you have said, we will ask you if you wish for your name to be used before we share it with anyone. Confidentiality will only be broken if there is a danger to the safety of yourself or someone else.

The Meeting Centre you go to will not be named in any reports, but we will describe it by the region it is located in.

What if I have a concern?

If you have any worries or questions please talk to us or someone else that you feel comfortable with. We are happy to arrange a time to come and talk with you and answer any questions. **For more info, contact:**

Thomas Morton

University of Worcester, Henwick Grove, Worcester, WR2 6AJ

Tel: 01905 542326 **Email: t.morton@worc.ac.uk**

Please remember:

You do not have to take part in this research study. It is your decision if you want to stop taking part in the research at any point and you do not have to give a reason why. If you do not decide to take part but then change your mind, that is fine as well. You can join in when you wish.

Whatever you decide, it will not affect your involvement with the Meeting Centre you go to.

This research has been approved by XXX

Thank you for taking time to read this information sheet.

Information Sheet for external partners working with and supporting Meeting Centres

External partners may include health, social care and third sector professionals, community-based partners and those involved with the local dementia pathway/DFC

Research Study Title - Get Real with Meeting Centres: A Realist Evaluation

You have received this information sheet because we would like you to take part in our research study. The following information explains what the research study is and what it would mean for you if you decide to get involved. Please take time to read over it and discuss the information.

What is the purpose of the study?

This study will look at how three Meeting Centres have got up and running and how they have kept going over the past few years. One of these is the Meeting Centre that you work with/ support. Many more Meeting Centres are now starting, so it is important we learn more about what has helped to make Meeting Centres successful. We have developed this study so that we can learn from it and develop tips and guidance for others. We will take all the information people tell us and map out how each Meeting Centre works, how it has worked over the past few years, and how best to plan for the future, going forward.

Why am I being asked to take part?

We are asking you to take part as you have direct experience of working with/supporting a Meeting Centre. We want to ask people for their knowledge and experience of how they think the Meeting Centre is run and what has made it a success.

Do I have to take part?

No, you do not have to take part in the study. You will be given a week to decide. If you decide against it, it will have no impact on your relationship with the Meeting Centre you currently work with/support. You can change your mind at any time and without giving a reason.

Morton T, *et al. BMJ Open* 2022; 12:e062697. doi: 10.1136/bmjopen-2022-062697

Information about people who have not consented to be involved will not be recorded as part of the study. You also have the right to withdraw your data after participation by which it will be destroyed on request.

If you decide that you would like to take part in this research study, you will first be asked to sign a consent form. We will then discuss with you the next steps of taking part.

What will happen if I wish to take part?

You will have the opportunity to be involved in informal interviews and group discussions over the coming months. You can make the decision on how much involvement you would like to have and which aspects you would like to get involved with.

The research study and COVID

We hope to be able to hold interviews and group discussions in person, but this is dependent on the COVID-19 restrictions. We are hopeful that in the near future it will be safe for us to visit each Meeting Centre. If so, we will abide by all current local and national guidance on what is safe to do when visiting. If this is not possible, then we will carry out our interviews and discussion group by online meetings (such as via Zoom) or by telephone and email.

Even if we can attend Meeting Centres, you can let us know whether you would like to be involved face-to-face or via an online method such as Zoom.

Will my involvement be kept confidential?

We keep the specific feedback in individual interviews confidential, but we will state our general findings in our final report. However, if something is disclosed during interviews or discussions that could impact upon the safety of yourself or others, confidentially may be breached if necessary to ensure safeguarding. Any feedback that we get from you will be checked for accuracy and anonymised through the removal of people's names and other personal information. When we write up our findings, we will use quotes from people we have spoken with. You will be asked your preferences on whether or not you would like your name

Morton T, *et al. BMJ Open* 2022; 12:e062697. doi: 10.1136/bmjopen-2022-062697

to be referenced by the quote. When the results of the study are presented, we will not use the name of the Meeting Centres involved in the study but will refer to them by region.

What are the possible disadvantages to taking part?

There are no obvious disadvantages to you taking part in the research. We are interested to hear your feedback.

What are the possible benefits of taking part?

By taking part in this study you will be providing valuable information that we can learn from to help other Meeting Centres to be a success. You get to share your opinions on Meeting Centres, what you think of them and how they might improve to help those who attend them. You may also help the Meeting Centre you work with/support, by helping us to understand how it works and what else we can do to help it in the future.

What will happen to the results of the study?

The results will give us the information to make recommendations to other Meeting Centres across the UK. They will be written up in a final report and shared with the National Institute of Health Research, who are funding the study. The results will also be used to write articles, reports or be included in presentations. We will make sure our results are made available to share with you once the study is complete.

Who is organising or funding the study?

This study is being led by the Association for Dementia Studies at the University of Worcester. It is funded by the **National Institute for Health Research (NIHR)**

Who has reviewed the study?

This research study has been reviewed and approved by XXX. A research ethics committee is a group of people who review research studies to ensure they protect the dignity, rights, safety and well-being of research participants and researchers.

For any further information please contact:

Shirley Evans – PI

s.evans@worc.ac.uk

Association for Dementia Studies

University of Worcester

Henwick Grove, Worcester, WR2 6AJ

Thomas Morton – Research Associate

t.morton@worc.ac.uk

Association for Dementia Studies

University of Worcester

Henwick Grove, Worcester, WR2 6AJ

Thank you for taking time to read this information sheet.

Information Sheet for Personal Consultees

A personal consultee is someone who advises on the preferences and past wishes of a person who is no longer able to give informed consent.

Research Study Title - Get Real with Meeting Centres: A Realist Evaluation

You have received this information sheet because we are inviting your relative/friend/the person you represent to take part in our research study. The following information explains what the research study is and what it would mean for the person you are acting as a personal consultee on behalf of to get involved. Please take time to read over it and discuss the information with other people before you make your decision.

What is the purpose of the study?

This study will look at how three Meeting Centres have got up and running and how they have kept going over the past few years. One of these is the Meeting Centre that your relative/friend /the person you represent goes to. Many more Meeting Centres are now starting, so it is important we learn more about what has helped to make Meeting Centres successful. We have developed this study so that we can learn from it and develop tips and guidance for others. We will take all the information people tell us and map out how each Meeting Centre works, how it has worked over the past few years, and how best to plan for the future, going forward.

Why am I being asked about this study?

We would like to involve your relative/friend/the person you represent in this study. Due to the fact that your relative/friend/the person you represent may be unable to decide for him/ herself, we are asking you to inform us on their behalf. We are asking them to take part as they have direct experience of going to a Meeting Centre. We want to ask people for their knowledge and experience of how they think the Meeting Centre is run and what has made it a success.

Does my relative/friend/the person I represent have to take part?

No. We are asking for your advice about whether they might object to taking part. You will be given a week to decide. They do not have to take part and their experience of going to their Meeting Centre will not change if they do not take part.

You may want to think about whether they have expressed an interest in taking part in research before. If you think they would **not object** to taking part, then please complete the **Consultee Declaration Form** and return it to the researchers (or a member of Meeting Centre staff to pass on to them).

If you feel that the person would not like to take part, it will have no impact on yours or their relationship with the Meeting Centre that they currently go to. You can change your mind at any time, without giving a reason. Information about people who have not consented to be involved will not be recorded as part of the study. You also have the right to withdraw your data after participation, in which case it will be destroyed on request.

If you think your relative/friend/the person you represent would like to take part in this research, we will arrange the next steps of taking part with both of you.

What will happen if I advise they would wish to take part?

If you say yes to your relative/friend/the person you represent taking part the following things will happen:

1. After reading this information sheet, you will need to complete a consultee declaration form as the person's consultee.
2. A member of the research team will then explain the study to you and your relative/ friend/the person you represent and invite them to take part in participating in an informal interview or a group discussion around the Meeting Centre that they go to.
3. You and your relative/friend/the person you represent can make the decision on how much involvement they would like to have and which aspects they would like to get involved with.

Morton T, *et al. BMJ Open* 2022; 12:e062697. doi: 10.1136/bmjopen-2022-062697

4. The researcher will take some notes of your relative/friend/the person you represent's feedback.
5. The research team will then look at this feedback and integrate it into their findings.

The research study and COVID

We hope to be able to hold interviews and group discussions in person, but this is dependent on the COVID-19 restrictions. We are hopeful that in the near future it will be safe for us to visit each Meeting Centre. If so, we will abide by all current local and national guidance on what is safe to do when visiting. If this is not possible, then we will carry out our interviews and discussion group by online meetings (such as via Zoom) or by telephone and email.

Even if we can attend Meeting Centres, you can let us know whether you would like to be involved face-to-face or via an online method such as Zoom.

Will the involvement of the person I represent be kept confidential?

We keep the specific feedback in individual interviews confidential, but we will state our general findings in our final report. However, if something is disclosed during interviews or discussions that could impact upon the safety of your relative/friend/the person you represent or others, confidentially may be breached if necessary to ensure safeguarding. Any feedback that we get from you will be checked for accuracy and anonymised through the removal of people's names and other personal information. When we write up our findings, we will use quotes from people we have spoken with, but these will be attributed to a pseudonym to retain anonymity in the case of people who are unable to give informed consent. When the results of the study are presented, we will not use the name of the Meeting Centres involved in the study but will refer to them by region.

What are the possible disadvantages to taking part?

There are no obvious disadvantages to your relative/friend/the person you represent taking part in the research. We are interested to hear their feedback.

What are the possible benefits of taking part?

By taking part in this study you will be providing valuable information that we can learn from to help other Meeting Centres to be a success. Your relative/friend/the person you represent will be able to have their say on Meeting Centres, what they think of them and how they might improve to help those who attend them. By being involved, they may also help the Meeting Centre that they go to by helping us to understand how it works and what else we can do to help it in the future.

What will happen to the results of the study?

The results will give us the information to make recommendations to other Meeting Centres across the UK. The results of the study will be written up in a final report and shared with the National Institute of Health Research, who are funding the study. The results will also be used to write articles, reports or be included in presentations. We will make sure our results are made available to share with you once the study is complete.

Who is organising or funding the study?

This study is being led by the Association for Dementia Studies at the University of Worcester. It is funded by the **National Institute for Health Research (NIHR)**

Who has reviewed the study?

This research study has been reviewed and approved by XXX. A research ethics committee is a group of people who review research studies to ensure they protect the dignity, rights, safety and well-being of research participants and researchers.

For any further information please contact:

Shirley Evans – PI

s.evans@worc.ac.uk

Association for Dementia Studies

University of Worcester

Henwick Grove, Worcester, WR2 6AJ

Thomas Morton – Research Associate

t.morton@worc.ac.uk

Association for Dementia Studies

University of Worcester

Henwick Grove, Worcester, WR2 6AJ

Thank you for taking time to read this information sheet.
