# Supplementary material for: Sustainability of locally driven centres for those affected by dementia: a protocol for the get real with meeting centres realist evaluation
Source: BMJ Open. 2022 May 2;12(5):e062697. doi: 10.1136/bmjopen-2022-062697 (PMC9062872; doi:10.1136/bmjopen-2022-062697)
Supplement: Supplementary data [file bmjopen-2022-062697supp004.pdf]

IRAS ID: 294636
Centre Number:
Study Number:
Participant Identification Number:

University of Worcester
Association for Dementia Studies

Research Study Consent Form

Title of Project: Get Real with Meeting Centres: A Realist Evaluation

Name of Researcher:

Please initial box

1. I confirm that I have read the information sheet dated 09/06/21 (version 0.4) for the above study. I have had the opportunity to consider the information, ask questions and have had these answered satisfactorily. ☐

2. I understand that my participation is voluntary and that I am free to withdraw at any time without giving any reason, without my involvement in the Meeting Centre or legal rights being affected. ☐

3. I understand that the information collected about me will be used to support other research in the future and may be shared anonymously with other researchers. ☐

4. I understand that an audio recording may be made of interviews and/or discussions that I take part in as part of this research, and do not object to this. ☐

5. I understand that quotes from what I say in my interviews and/or discussions may be used when the findings of this study are written up. ☐

   With regards to the above, please tick one of the below:

   I would like my name to be changed on any quotes used from me. ☐

   I am happy my name to be used on any quotes from me. ☐

Please turn over…

6. I agree to take part in the above study. ☐

7. I know who to contact if I have any concerns about this research. ☐

Name of participant:	Date:	Signature:
____________________	_______________	_______________

Name of person taking consent:	Date:	Signature:
____________________	_______________	_______________

Morton T, *et al. BMJ Open* 2022; 12:e062697. doi: 10.1136/bmjopen-2022-062697

IRAS ID: 294636

Centre Number:

Study Number:

Participant Identification Number for this study:

Consultee Declaration Form

Title of Project: Get Real with Meeting Centres: A Realist Evaluation

Name of Researcher:

Please initial box

1. I ……………………..….. have been consulted about ………………….……….'s participation in this research project. I have had the opportunity to ask questions about the study and understand what is involved. ☐

2. I understand that I do not have to act as consultee and I can change my mind at any time. ☐

3. In my opinion he/she would have no objection to taking part in the above study. ☐

4. I understand I can request he/she is withdrawn from the study at any time, without giving a reason and without his/her involvement in the Meeting Centre being affected. ☐

5. I understand that the information collected about him/her will be used to support other research in the future, and may be shared anonymously with other researchers. ☐

6. I understand that an audio recording may be made of interviews and/or discussions that he/she takes part in as part of this research, and in my opinion he/she would not object to this. ☐

7. I understand that quotes from what he/she says in interviews and/or discussions may be used when the findings of this study are written up, with names changed to preserve anonymity, and in my opinion he/she would not object to this. ☐

Please turn over…

8. I know who to contact if I have any concerns about this research. ☐

Name of Consultee: Date: Signature:

______________________ ________________ ________________

Relationship to participant:

____

Person undertaking consultation:

Name Date Signature

______________________ ________________ ________________

To be completed by researcher prior to seeking consultee declaration (tick one),

☐ 1. Potential participant showed signs of a positive or interested attitude when going through the participant information

☐ 2. Potential participant showed signs of a negative or anxious attitude toward the research or taking part in it when going through the information

Consent Form v0.4 – Get Real Study 09/06/2021
