# Supplementary material for: Sustainability of locally driven centres for those affected by dementia: a protocol for the get real with meeting centres realist evaluation
Source: BMJ Open. 2022 May 2;12(5):e062697. doi: 10.1136/bmjopen-2022-062697 (PMC9062872; doi:10.1136/bmjopen-2022-062697)
Supplement: Author's manuscript [file bmjopen-2022-062697.draft_revisions.pdf]

BMJ Open

BMJ Open is committed to open peer review. As part of this commitment we make the peer review history of every article we publish publicly available.

When an article is published we post the peer reviewers' comments and the authors' responses online. We also post the versions of the paper that were used during peer review. These are the versions that the peer review comments apply to.

The versions of the paper that follow are the versions that were submitted during the peer review process. They are not the versions of record or the final published versions. They should not be cited or distributed as the published version of this manuscript.

BMJ Open is an open access journal and the full, final, typeset and author-corrected version of record of the manuscript is available on our site with no access controls, subscription charges or pay-per-view fees (http://bmjopen.bmj.com).

If you have any questions on BMJ Open's open peer review process please email info.bmjopen@bmj.com

BMJ Open

The sustainability of locally-driven centres for those affected by dementia: A protocol for the Get Real with Meeting Centres realist evaluation

Journal:	*BMJ Open*
Manuscript ID	bmjopen-2022-062697
Article Type:	Protocol
Date Submitted by the Author:	08-Mar-2022
Complete List of Authors:	Morton, Thomas; University of Worcester, Association for Dementia Studies<br>Evans, Shirley; University of Worcester, Association for Dementia Studies<br>Brooker, Dawn; University of Worcester, Association for Dementia Studies<br>Williamson, Tracey; University of Worcester, Association for Dementia Studies<br>Wong, Geoff; Oxford University, Nuffield Department of Primary Care Health Sciences<br>Tinelli, M ; London School of Economics and Political Science, PSSRU<br>Frost, Faith; University of Worcester, Association for Dementia Studies<br>Bray, Jennifer; University of Worcester, Association for Dementia Studies<br>Hullah, Nigel; 3 Nations Working Group for Dementia
Keywords:	Dementia < NEUROLOGY, Organisation of health services < HEALTH SERVICES ADMINISTRATION & MANAGEMENT, SOCIAL MEDICINE, Old age psychiatry < PSYCHIATRY, Organisational development < HEALTH SERVICES ADMINISTRATION & MANAGEMENT, QUALITATIVE RESEARCH

SCHOLARONE™
Manuscripts

The sustainability of locally-driven centres for those affected by dementia: A protocol for the Get Real with Meeting Centres realist evaluation

Authors:

Thomas Morton, Association for Dementia Studies, University of Worcester, Worcester, UK.

Shirley B. Evans, Association for Dementia Studies, University of Worcester, Worcester, UK.

Dawn Brooker, Association for Dementia Studies, University of Worcester, Worcester, UK.

Tracey Williamson, Association for Dementia Studies, University of Worcester, Worcester, UK.

Geoff Wong, Nuffield Department of Primary Care Health Sciences, University of Oxford, Oxford, UK.

Michela Tinelli, Care and Policy Evaluation Centre, London School of Economics and Political Science, London, UK.

Faith Frost, Association for Dementia Studies, University of Worcester, Worcester, UK.

Jennifer Bray, Association for Dementia Studies, University of Worcester, Worcester, UK.

Nigel Hullah, 3 Nations Working Group for Dementia, Swansea, UK

Corresponding Author:

Dr Shirley B. Evans, Association for Dementia Studies, School of Allied Health and Community,

University of Worcester, Henwick Grove, Worcester WR2 6AJ.

Email: *shirley.evans@worc.ac.uk*. Tel: 01905 542664

For peer review only

The sustainability of locally-driven centres for those affected by dementia: A protocol for the Get Real with Meeting Centres realist evaluation

Thomas Morton,[1] Shirley B. Evans,[2] Dawn Brooker,[3] Tracey Williamson,[4] Geoff Wong,[5] Michela Tinelli,[6] Faith Frost,[7] Jennifer Bray,[8] Nigel Hullah[9]

ABSTRACT

Introduction: Improving support for people with early to moderate dementia to live at home in their communities is a global public health goal. Community adult social care is not robust in many parts of the UK, however, with the pandemic increasing pressure on services for this population. Community-led interventions can play a key role in supporting people post-diagnosis, helping delay decline, but many interventions struggle to sustain beyond 1-2 years. Meeting Centres (MCs) are one such intervention, which many UK community groups find attractive and achievable. However, it is not understood how these communities can ensure they are putting in place strategies that will help them sustain in the longer term, beyond start-up phase.

Methods and analysis: This Realist Evaluation aims to understand the factors affecting sustainability of MCs in rural areas and learn lessons from MCs that have sustained beyond three years. Data will be collected using mixed methods: interviews and group discussions with stakeholders involved at every level in three case study locations in England and Wales, analysed with Soft Systems modelling; a Discrete Choice Experiment exploring what people across the UK value and are willing to pay for MCs, analysed with regression modelling. All data will be synthesised using a Realist logic of analysis to build a theoretical model of how, why, for whom, in what contexts and to what extent MCs can be successfully implemented for the long-term.

Ethics and dissemination: As participants may lack capacity for informed consent, favourable ethical opinion was sought from a Health Research Authority research ethics committee. Resulting recommendations will be of interest to stakeholders including those commissioning, planning,

running, supporting or attending MCs, as well as policymakers and healthcare professionals. Knowledge will be shared with emerging MCs to help accelerate scale up of this intervention.

Key words: *Dementia, post-diagnostic support, psychosocial intervention, integrated care, organisation of health services, community medicine, social medicine, old age psychiatry*

Strengths and limitations of this study

- A Realist approach is well suited to accommodate and account for the complexity of such ‘real life’ intervention programmes, as implemented under different conditions in different settings, to extract transferable conclusions.
- This study is designed to draw upon and disseminate a wide range of knowledge and expertise from people with extensive first-hand experience in tackling the issues involved in keeping a community-led dementia intervention running long-term.
- The Meeting Centre is an intervention type that is rapidly growing in popularity across the UK at this current time, hence this research well placed to offer timely practical insights to help multiple new community-led interventions of this type become established and avoid common pitfalls that may threaten sustainability.
- This study is designed to gather evidence for how to successfully sustain a Meeting Centre for people affected by dementia or similar community-led intervention in a rural UK context, not data on the effectiveness or otherwise of this particular intervention type.
- This research cannot guarantee solutions to all of the challenges to the sustainability of a community-led intervention such as a Meeting Centre; instead it may highlight some challenges that are insurmountable given current approaches and policy, regarding health and social care systems.

For peer review only

INTRODUCTION

Supporting people living with dementia (and those that, in turn, care for or otherwise support them) to live as well as possible in their communities, with timely psychosocial support, is a global public health goal[1]. However, support following a diagnosis of dementia is patchy across the UK[2], with people and families in some areas lacking any formal proactive support beyond occasional contact with primary care and third sector. With an aging population[3] and increasing pressure on already stretched health services[4] policy has for some time pointed to the need to move towards a model of social care where more people are cared for and supported at home, in the community. Improving provision of early, post-diagnosis support, improving support for family/informal carers and improving support for integrated care (involving the voluntary and independent sectors) – all in a more dementia-friendly community environment – are contemporary UK Government priorities for dementia care[2].

However, multiple pre-pandemic reports described a climate where the state of social care provision – mainly delivered piecemeal by private and third-sector organisations – is "precarious and dysfunctional" in many parts of the country[4] and in some areas has "broken down" creating "care deserts"[5]. There is an associated reliance on informal carers (e.g. family members) to step in to meet the needs of loved ones, but there is a growing recognition that informal carers' own health and wellbeing is often negatively impacted by their caring activities[6]. The detrimental health impact of social isolation and loneliness is also increasingly being recognised[7-8], with survey data revealing 60% of people living with dementia report loneliness, isolation and losing touch with people in their lives since diagnosis, around a quarter feeling they are not part of their community and that people avoid them[9]. Family carers can also be subject to such loneliness and isolation[10].

For peer review only

There have been various attempts to mitigate these challenges in communities across the UK, in the form of groups and activities for people with dementia and family/informal carers. These aim to serve a number of functions. However, there are significant gaps in social care for people affected by dementia across the UK[5-6,9]. Care systems are unprepared for the forecasted doubling of the number of people living with dementia (1.6 million) and tripling of social care costs by 2040[11]. Scaling up provision of evidence-based community initiatives for people with dementia and those that support them is imperative[12-18]. The benefits of community-based initiatives are now being recognised[14-18]. However, they face a variety of challenges in sustaining long-term. These challenges and how to meet them are much talked about in the dementia care policy, rhetoric and practice arenas but have received very little research attention, as identified in the SCI-Dem review [2018-20][19-20]. This research also showed many community initiatives are not sustained beyond 1-2 years.

Meeting Centres

Meeting Centres (MCs) for people affected by dementia are a community initiative based upon a successful Dutch model[12-13,21] that have emerged in the UK since 2015. MCs are distinct from day care, supporting both people with dementia and those that support them (e.g. children, partners and friends) together, and connecting people to each other and their community. They build on Dementia Friendly Communities and are a step up in support from Dementia Cafes. At their heart is a small social club (15 people per day plus supporting family, friends and carers), based in an ordinary community building, close-by to where people live. They typically operate up to three times per week, providing people the chance to build friendships, peer support, understand their problems, get help and prepare for the future. Evidence-based post-diagnostic interventions are also provided in MCs, geared to the needs of members and facilitated by a small team of staff and volunteers trained in person-centred dementia care and the Adaptation-Coping Model[22]. Following substantial European research [MeetingDem: 2014–17][14,23-25], MCs were recommended as a social care intervention for those affected by dementia[14] and found to help people, their families and

For peer review only

communities build resilience for the longer term[12-15,26-32]. The first MC in the UK opened in 2015, and there are now more than 30 either running or with funding to open in 2022, with accelerated interested from communities around the UK since lockdown restrictions were eased in 2021[33].

The focus is now turning from how to establish these interventions, to how to keep them going long-term, in the face of a challenging social care-funding climate. To date, early adopter MC sites have devised different strategies to mitigate against threats and circumstances affecting their successful continuation. A better understanding is needed of the issues MC stakeholders have faced and are likely to face, and what can be learnt from this to prevent 'reinvention of the wheel' and help ensure sustainability. This is particularly true in rural communities where people and families living with dementia face increased barriers to being able to access support, guidance and connection[34]. No MC-focused studies have yet investigated the factors that are key for the sustaining MCs after the start-up stage, and there is little focussing on this aspect with regards to other community-based interventions broadly serving a similar function and demographic. If these kinds of interventions cannot survive long term, the gap in provision will remain and the situation can be expected to worsen significantly with the rise in numbers of people living with dementia needing support.

METHODS AND ANALYSIS

Research question and overall aim: The understand the factors affecting the sustainability of Meeting Centres for people affected by dementia in rural areas, how these can best be tackled, and what lessons can be gleaned for emerging Meeting Centres.

Objectives

1: To empirically test the theory developed by the SCI-Dem review regarding the factors involved in sustainability of a community intervention for people affected by dementia.

For peer review only

2: To produce and in-depth transferable understanding of the key factors that may threaten the long-term delivery of an MC in the form of a refined Realist programme theory.

3: To explore people's willingness to pay for MC provision via a Discrete Choice Experiment (DCE) with those who support people living with dementia, triangulated with the perspectives of people living with dementia.

4: From the programme theory and DCE above, to build a model of how best to design, implement and deliver an MC under different conditions so it has the best chance of sustaining long-term.

5: To develop evidence-informed guidance materials for those in practice and evidence-informed recommendations for commissioning and policy level.

Project overview

This Realist Evaluation[35] will comprise primary data collection from three MC research sites, that have each taken different approaches to serving different rural community settings, to investigate what works, under what circumstances, for whom, how and why, regarding configuring MCs for long-term sustainability. Data collection will comprise interviews and focus group discussions with participants in a range of roles relating to the MC under investigation, along with documentary and demographic data from each site. We will produce a case study model of each MC site with the aid of Soft Systems Methodology (SSM)[36]. These models will be synthesised and analysed using a Realist logic of analysis[35] to generate theoretical causal chains of how contexts (background circumstances) can trigger mechanisms (responses and processes within people and organisations) to produce desired or undesired outcomes. We will also conduct a DCE survey regarding what activities and elements of MCs members most value and how much they are willing to pay for these, which will also feed into the overall Realist analysis. This work will be split across five work packages. **Figure 1** shows an overview of the study framework.

[Figure 1 here]

For peer review only - http://bmjopen.bmj.com/site/about/guidelines.xhtml

The use of Systems approaches to complement Realist thinking is an area of growing interest with multiple successful examples integrating the two in recent years[37-38]. Both Realist and Systems approaches are appropriate for this research as they aim primarily to deal with, and understand, the complexity of systems with human actors in real-world settings. Social care interventions such as MCs tend to be especially complex as they can involve multiple agencies and are embedded in a wider community setting, often with informal and impermanent elements making up part of how they work. Realist approaches focus on explaining the causal mechanisms of action that underlie complex programmes or interventions, to explain why they may be successful in some instances but not in others[39]. Soft Systems Methodology[36] (SSM) describes a process of enquiry to uncover real-world complexity by consulting those involved with a programme, to build up a conceptual model and determine what action can be taken to change things. The approach is designed to tackle organisational problems where the exact nature of the problem may not be agreed upon and need investigating. Hence, the issue of the sustainability of an MC programme, with all of the factors that could involve in a complex community-based setting, is a good fit for a combination of Realist and Systems approaches.

Patient and Public Involvement

Patient and participant involvement (PPI) will be channelled through each MC research site, led by dedicated members of the project team including a lay co-applicant who is living with dementia. Prior to the start of this study, PPI was carried out with members of one MC and lay members of the UK Meeting Centre Support Programme (UK-MCSP) National Reference Group, which includes members of DEEP (Dementia Engagement and Empowerment Project network)[40] and TIDE (Together In Dementia Everyday carers' network)[41]. Views were sought about the importance of the study, its design, focus, recruitment approaches, factors likely to affect participation, payment/other reward for PPI, dissemination, approaches to PPI in the study, support needs (e.g. PPI training/development) and interest in further involvement if the study was funded. PPI representatives will be fully

For peer review only

supported to input into data collection processes and materials, synthesis and interpretation of data, and the creation of recommendations and resources for dissemination, as well as channels of dissemination. We will also continue to work with the UK-MCSP National Reference Group, to provide steering input.

Work package 1 - Stakeholder group engagement and enquiry

Iterative stakeholder consultation throughout a project is a standard part of Realist Evaluation[42]. Stakeholders will include patient and public representatives (whose involvement is covered in the section above) as well as stakeholder professionals with experience of community dementia support. The latter will also be consulted regarding data collection processes and materials, synthesis and interpretation of data, creation of recommendations and resources for dissemination, and channels of dissemination. This work is organised as work package 1 as it is central to the progress of the evaluation.

Work package 2 – Data gathering and Meeting Centre case studies

We have identified three MCs in different rural communities that have been operational for over three years and that meet the Essential Features of Meeting Centres[43]: One in a small market town in Herefordshire (opened February 2016; rural; deprivation rank[44] around MC: 3,288 of 32,844 neighbourhoods in England); one in a larger market town in Worcestershire (opened September 2015; semi-rural; deprivation rank[44]: 17,429 of 32,844 neighbourhoods in England); One rural county in Mid Wales with four federated small town MCs run by the same organisation (opened March 2017; rural; deprivation rank[45]: areas ranging from 284 to 1,687 of 1,909 neighbourhoods in Wales). Study sites have been selected purposefully, as they have been able to continue operating for at least two years prior to the commencement of this study, notwithstanding some necessary pausing or alteration of activities during COVID-19 restrictions. In addition to different geographic and demographic factors, MCs at each site have taken their own individual approach to the implementation and delivery of the service.

For peer review only

Participants will likewise be selected purposefully for their role and involvement in each MC, and for the experience they might bring regarding issues outlined in the research question and objectives. The project team will work with MC leads to identify appropriate potential participants in each role and approach those in roles outside the MC itself. MC staff will approach MC attendees to invite them to participate in the study, help them to better understand the participant information and consent process to make an informed decision on taking part.

Interviews and focus groups will take place with those involved at every level, in the following manner per MC, as shown in **Table 1**:

**Role of participant in MC**	**Method of data collection**	**Number of participants**
MC attendees (people living with dementia)	Focus group and/or one to one interview (individual or supported by a partner)	6
MC attendees (people supporting someone with dementia)	Focus group and/or one to one interview	6
MC staff and volunteers	Focus group and/or one to one interview	6
Those involved with governance at each MC	One to one interview	4
Health/social care/third sector professionals involved in the local dementia care pathway	One to one interview	4

For peer review only

Other stakeholders involved in local dementia strategy e.g. Dementia Friendly Communities Programme	One to one interview	4
		**Total:30**

Table 1. Participants and methods of data collection

We anticipate 30 participants per MC. This number should give us a range of perspectives per type of participant, at different levels, to draw on to create SSM conceptual models, while also being realistic in terms of numbers available to take part at each MC and practically manageable within the scope and timeframe of the study. Interviews and discussions are anticipated to take between 20 minutes and one hour and will be conducted on site at each MC where possible, though we anticipate a proportion will be conducted via virtual video conferencing or telephone due to the impact of the COVID -19 or participant preference.

Interview schedules were developed and piloted with stakeholder input and can be found in **Supplementary file 1**. The development of these were guided in part by the factors involved in the sustainability of community-based interventions found in the SCI-Dem Realist Review[19-20] (summarised in **Supplementary file 2**) but also a modified SSM "BATWOE" structure as outline in **Table 2.**

**B**	Beneficiaries (who is the system aimed at helping, e.g. people living with dementia and those that support them)
**A**	Actors (people's roles and functions in the system, e.g. staff, volunteers, governors, referrers, social care professionals, community stakeholders)

For peer review only

**T**	Transformations (i.e. going from start-up MC to established MC to stable and thriving MC)
**W**	Worldview (e.g. how do things work regarding sustainability, what are the challenges and what should be done?)
**O**	Ownerships (i.e. who or what can influence or thwart success of an MC)
**E**	Environment (i.e. what are the background contextual factors that could boosts or constrain success?)

Table 2. The elements of the SSM "BATWOE" structure[36-37].

The content of interview and discussion transcripts pertaining to the sustainability of MCs will be extracted and categorised by theme (generated both deductively and inductively) using NVivo qualitative data analysis software[46]. Two researchers will undertake this and independently theme and categorise 10% of each other's workload and compare and discuss any discrepancies for standardisation purposes. Any remaining disagreements will be discussed with the whole research team, as will the final list of data categories. Data will then be analysed using SSM procedures[36], applying a sequence of steps to the data from each MC site to build a conceptual model of how things work which will be returned to participants to review. This will act as the basis for developing a Realist programme theory with all data combined (see work package 4)

Work package 3 - A DCE to measure people's willingness to pay for successful MCs

Successful implementation of evidence-based services in health and social care depends largely on the fit of the services with the values and priorities of stakeholders who are shaping and participating in their delivery and use[47]. A flexible health economics tool for measuring choices in health and social care-related settings is the DCE[48], which measures preferences from individual decision-makers over alternative scenarios (or service provisions). Each alternative is described by

For peer review only

several attributes (or characteristics) and the choices made between two or more competing scenarios subsequently determine how preferences are influenced by each attribute (e.g., which attributes are valued as well as their relative importance). It can also provide a measure of the overall value attached to different alternatives (and identify optimal service provision that meets stakeholder requirements and have the best chance of sustainability in the long term). When a cost attribute is included, the DCE technique can also allow weighing of the benefits and costs of service provisions and calculating: how much stakeholders may be willing to pay for a particular service provision and measure how their willingness to pay may vary from current provision to their preferred option. Hence a DCE survey will be developed to measure people's preferences for what can MC provides in terms of types of activities, social opportunities and emotional support, as well as the frequency of meetings, their availability and costs.

The DCE follows steps as laid out in the standard guidelines[49]. The attributes (characteristics) and their various levels will be informed by the Essential Features of a Meeting Centre[43] and the Adaptation-Coping Model[22], as well as data from our three case study sites regarding the typical range of cost to members and days/time open or available per week. Qualitative data regarding what members value about MCs, from early Work Package 2 interviews, will be then used to validate and refine the attributes and levels. PPI stakeholders will be also consulted on the development of the presentation, wording and format of the survey. Due to the cognitive load involved in completing the survey, our target group will be people who support/care for an attendee who is living with dementia, reporting preferences on their behalf. Triangulation focus groups (n=3-6 per MC site) will be conducted with people living with dementia at each MC to ensure the views of this population are not excluded. The questionnaire will be distributed via staff at all UK MCs, as either an online survey or paper copy, depending on preference, with the aim of securing responses from more than 300 people[49-50]. Efficient experimental design techniques will be applied to create the DCE choice set[51] and data will be modelled using logit techniques (NLOGIT 6 software).

For peer review only

Work package 4 - Realist theory refinement and development of materials for practice

Data from all work packages, categorised and organised as themes, will be further analysed using the same Realist logic of analysis[35] as the SCI-Dem Realist Review[19-20], to develop a Realist programme theory. This second stage of analysis is needed to understand causation – i.e. how differing contexts in different MCs trigger different mechanisms (the hidden causal processes within people and organisations) to cause desired or undesired outcomes. This will test the programme theory produced during the SCI-Dem review, and act as a basis for developing recommendations and materials that explain how to best implement community-based interventions to sustain past the start-up phase, in a variety of settings. As it is developed, this programme theory will be presented back to stakeholders in each MC (see Work Package 1) for feedback and advice that will be used to further validate and refine it. We will use this understanding to develop (amongst other things) tips on best practice, what pitfalls to avoid and what challenges may need to be planned for, grounded in the experiences and models of working of those involved with the three MCs at every level. Materials to disseminate this learning to those in practice, and at a commission level, will be developed in collaboration with stakeholders.

Work package 5 – Investigation of who MCs do not reach or benefit

In December 2021 the NIHR approved an expression of interest to add a further work package to the Get Real study. This work package will investigate who is not being reached by MC support and why, in the following ways: A comparison of demographic data regarding who attends each case study MC and whether they are representative of the population of their communities (and if there are any demographic groups clearly under-represented); additional interviews with existing stakeholders involved in the MC referral process specifically regarding why referrals might not be made or declined; identification, recruitment and additional interviews with dyad pairs (n=10) who have been made are aware of an MC but have decided not to attend or stopped attending, to understand what happened and why. This will again feed into Work Package 4.

For peer review only

ETHICS AND DISSEMINATION

Favourable ethical opinion for the whole study was sought from a Health Research Authority (HRA) Research Ethics Committee (REC) prior to starting recruitment and data collection. This was a requirement of the NIHR funders and in any case deemed necessary because some participants may lack the capacity to provide informed consent, or their ability to consent may change over time. Under the terms of the Mental Capacity Act 2005[52], people who lack the capacity to consent cannot be included in research unless the research concerns their condition. This research is concerned with improving the implementation and sustainability of social care interventions related to the condition of dementia, in order to improve the provision of support for the benefit of people living with dementia and those who support them. Favourable ethical opinion was given by Wales REC4 (21/WA/0185).

Consent and risk of distress

Participant information (see **Supplementary file 3**) and consent documents (see **Supplementary file 4**) were developed for a range of possible participants in line with HRA guidance[53]. It was felt important to include MC attendees in the research for two reasons: 1) to ensure the perspectives of the people MCs are designed for are fully and authentically represented in line with a "Nothing about us, without us" ethos; 2) to access key knowledge and experience, because attendees are in a position to offer key first-hand perspectives not directly available to others such as staff and governors, particularly regarding the factors that can encourage or act as a barrier to engaging with and attending a local MC. In order to undertake research ethically with these participants, the research team developed sensitive and relevant practices of informing and negotiating consent to participate. **Figure 2** outlines the process of determining ability or provide informed consent:

[Figure 2 here]

For peer review only

The focus of the interviews, group discussions and questionnaire questions will be the MC, people's experiences and opinions of it and preferences regarding what it provides. This focus is not expected to include topics that might be overly personal, sensitive, embarrassing or upsetting. Nevertheless, there is a risk semi-structured interviews and discussion may stray into personal or sensitive areas that participants may not be comfortable with. For this reason, question topics will be explained to participants (and their consultee if they have one) before and interview or group discussion. Participants will also be told they do not have to answer any question they are not comfortable with and that they can stop the interview or leave the discussion at any time. The researchers will also be on alert for any signs of distress and will pause proceedings if a participant shows signs of discomfort or upset, or of reluctance to take part, at any time before or during interview or group discussion. The participant (and their consultee if they have one) will be asked if they are happy to continue, would prefer to re-arrange for another time, or withdraw altogether. If there is any sign of discomfort with a sensitive or personal topic that is not necessary to discuss, researchers will automatically move the conversation on to a topic that is not personal or sensitive. Participants who are attendees of the MC will undertake interviews and discussions at the Meeting Centre itself, with trained staff on hand to help if they do become distressed or upset. Questions will also be framed in terms of how to overcome challenges for success, and what can be learned to help success in the future, and piloted to ensure there is no suggestion that our research means a MC's future is under threat or that blame for any perceived failings in running it is being sought, which could be upsetting for participants.

The DCE questionnaire will be anonymous and only asks for opinions and preferences, hence there are not anticipated to be any risks in taking part. Participant information explaining the nature and objectives of the questionnaire, and consent questions, will be presented as part of the questionnaire itself. We will also hold focus group discussions with participants living with dementia in our MC case study sites, on the same questions covered in the DCE questionnaire, to triangulate, with an identical consent process to the other interviews and group discussions.

For peer review only

Regarding COVID-19 risks, we will only undertake face-to-face data collection if local and national guidance allows visitors to MCs and the MC and potential participants feel safe to do so. This situation will be continually monitored. Where face-to-face data collection is not possible or agreed, we will move to online data collection supplemented by telephone calls where preferred.

Data management and confidentiality

All data gathered that may identify participants will be kept in password protected files and folders on the University of Worcester's secure cloud-based storage, with unique participant identification codes used in data storage, known only to the research team. This includes interview and discussion recordings and transcripts, consent documentation and any personal data collected to maintain contact with participants. Where physical copies of data are necessary, they will be kept in locked cabinets on University premises. Face-to-face meetings will be recorded digitally on an encrypted recording device, online meetings using facilities provided by that online platform, with recordings transferred at the first opportunity and deleted once transcribed by a member of the research team or a trusted external transcription service and checked for accuracy.

Transcripts will be anonymised through the removal of names and other personal information. However, it should be noted that for the sake of analysis some information may be necessary to retain on a participant's role within the system of each MC, or information specific to the local context, which may make jigsaw identification possible. Hence participants will be alerted to this when taking consent and their preferences on anonymity and identification will be gathered and checked before reporting. In reporting, MCs will not be identified specifically by name or town, but only by region and pertinent demographic factors.

Dissemination

The UK-MCSP[54], led by the University of Worcester, has created a UK-wide Community of Learning and Practice comprising 300 organisations involved or interested in providing MCs in their

For peer review only

communities, with more than 30 MCs now on-stream and more planned across the country. There is also a National Reference Group comprising 30 national organisations drawn from policy and practice. This networked community of stakeholders will be consulted to help co-create accessible resources and disseminate them according to their preferences, to ensure knowledge from this research is accessible to those involved in the day-to-day governance, management and running of MCs in the UK. This will also involve workshop activities with PPI representatives/stakeholders within the case study MCs. Dissemination will take place through these MC network channels, as well as to a wider audience through practitioner workshops, webinars, blogs, newsletters and social media. Learning form this study will also be incorporated into training for emerging and existing MC personnel provided by the University of Worcester.

In addition to academic publications and conference presentations, outputs will include an accessible publication and website downloads for a non-academic audience that will detail the three case studies and overall analysis, useful for all in similar community settings looking for a flexible template that they might implement; there will also be published evidence-based guidelines for commissioners and providers of community-based interventions for people affected by dementia. Specifically, Worcestershire County Council will use knowledge from this research to support new MCs in the county. A publicly accessible report summary will be available on University of Worcester Association for Dementia Studies website[55] post-study and findings will be promoted widely at MCs to reach study participants via posters, presentations and leaflets. A full study report will be made available on request.

Word count 4,348

Figure legends

Figure 1. Overview of Get Real with Meeting Centres project.

For peer review only

Figure 2. Consent process flow diagram.

Author affiliations and email

1 Association for Dementia Studies, University of Worcester, Worcester, UK. *t.morton@worc.ac.uk*

2 Association for Dementia Studies, University of Worcester, Worcester, UK. *shirley.evans@worc.ac.uk*

3 Association for Dementia Studies, University of Worcester, Worcester, UK. *d.brooker@worc.ac.uk*

4 Association for Dementia Studies, University of Worcester, Worcester, UK. *Tracey.Williamson@wales.nhs.uk*

5 Nuffield Department of Primary Care Health Sciences, University of Oxford, Oxford, UK. *geoffrey.wong@phc.ox.ac.uk*

6 Care and Policy Evaluation Centre, London School of Economics and Political Science, London, UK. *M.Tinelli@lse.ac.uk*

7 Association for Dementia Studies, University of Worcester, Worcester, UK. *f.frost@worc.ac.uk*

8 Association for Dementia Studies, University of Worcester, Worcester, UK. *j.bray@worc.ac.uk*

9 3 Nations Working Group for Dementia, Swansea, UK. *nigelhullah@aol.co.uk*

Corresponding Author Dr Shirley Evans, Association for Dementia Studies, School of Allied Health and Community, University of Worcester, Henwick Grove, Worcester WR2 6AJ. Email: *shirley.evans@worc.ac.uk*. Tel: 01905 542664

Twitter Follow Thomas Morton @ThomasMortonADS

Author Contributions DB, SBE and TM conceptualised the study with input from TW, GW, MT, FF, JB and NH. TM wrote the first draft of this manuscript. SBE, DB, TW, GW, MT, FF, JB and NH critically contributed to and refined this manuscript. All authors have read and approved the final manuscript.

For peer review only

Acknowledgements The authors would like to thank all those who shared their invaluable experience and contributed to advising and guiding this project as a stakeholder consultant, and all those involved with Meeting Centres, whether running, attending, supporting or in governance, who have agreed to take part in this research or facilitate others to do so.

Funding This paper presents independent research funded by the National Institute for Health Research (NIHR) under its Research for Patient Benefit (RfPB), Research for Social Care Programme (Grant Reference Number NIHR201861). The views expressed are those of the authors and not necessarily those of the NIHR or the Department of Health and Social Care.

Competing interests GW is Deputy Chair of the National Institute for Health Research Health Technology Assessment Prioritisation Committee: Integrated Community Health and Social Care (A).

Ethics approval This project has been reviewed by Health Research Authority research ethics committee Wales REC 4. REC reference: 21/WA/0185; IRAS project ID: 294636.

ISRCTN registration number 10332079

Open Access This is an Open Access article distributed in accordance with the terms of the Creative Commons Attribution (CC BY 4.0) license, which permits others to distribute, remix, adapt and build upon this work, for commercial use, provided the original work is properly cited. See: http://creativecommons.org/licenses/by/4.0/

REFERENCES

For peer review only

1. World Health Organisation. *Global action plan on the public health response to dementia 2017-2025.* Geneva: World Health Organisation, 2017. https://www.who.int/publications-detail/global-action-plan-on-the-public-health-response-to-dementia-2017---2025

2. Department of Health. *Prime Minister's challenge on dementia 2020.* London: Department of Health, 2015. https://assets.publishing.service.gov.uk/government/uploads/system/uploads/attachment_data/file/414344/pm-dementia2020.pdf

3. Office for National Statistics (ONS). *Living longer: Caring in later life.* London: ONS, 2019. https://www.ons.gov.uk/peoplepopulationandcommunity/birthsdeathsandmarriages/ageing/articles/livinglongerhowourpopulationischangingandwhyitm atters/2019-03-15

4. Care Quality Commission. *The state of health care and adult social care in England 2018/19.* Newcastle-upon-Tyne: Care Quality Commission, 2019. https://www.cqc.org.uk/sites/default/files/20191015b_stateofcare1819_fullreport.pdf

5. Incisive Health. *Care deserts: the impact of a dysfunctional market in adult social care provision.* London: Incisive Health, 2019. https://www.ageuk.org.uk/globalassets/age-uk/documents/reports-and-publications/reports-and-briefings/care-- support/care-deserts---age-ukreport.pdf

6. Age UK. *Briefing: Health and Care of Older People in England 2019.* London: Age UK, 2019. https://www.ageuk.org.uk/globalassets/ageuk/documents/reports-and-publications/reports-and-briefings/health--wellbeing/age_uk_briefing_state_of_health_and_care_of_older_people_july2019.pdf

7. Cornwell EY, Waite LJ. Social disconnectedness, perceived isolation and health among older adults. *J Health Soc Behav* 2009;50:31–48.doi.org/10.1177/002214650905000103

8. Holt-Lunstad J, Smith TB, Baker M, *et al.* Loneliness and social isolation as risk factors for mortality: a meta-analytic review. *Perspect Psychol Sci* 2015;10:227–237.doi.org/10.1177/1745691614568352

9. Alzheimer's Society. *A lonely future: 120,000 people with dementia living alone, set to double in the next 20 years.* London: Alzheimer's Society, 2019. https://www.alzheimers.org.uk/news/2019-05-15/lonely-future-120000-people-dementia-living-alone-set-double-next-20-years

10. Brodaty H, Donkin M. Family carers of people with dementia. *Dialogues Clin Neurosci* 2009;11:217–228.pmid:http://www.ncbi.nlm.nih.gov/pubmed/19585957

11. Wittenberg R, Hu B, Barraza-Araiza L, *et al. Projections of older people with dementia and costs of dementia care in the United Kingdom, 2019-2040 (CPEC Working Paper 50).* London: LSE, 2019. https://www.alzheimers.org.uk/sites/default/files/2019-11/cpec_report_november_2019.pdf

12. Dröes RM, Breebaart E, Meiland FJ, *et al.* Effect of Meeting Centres Support Program on feelings of competence of family carers and delay of institutionalization of people with dementia. *Aging Ment Health* 2004;8:201-211. doi.org/10.1080/13607860410001669732

13. Dröes RM, Meiland FJM, Schmitz M, *et al.* Effect of combined support for people with dementia and carers versus regular day care on behaviour and mood of persons with dementia: results from a multicentre implementation study. *Int J Geriatr Psychiatry* 2004;19:1-12.

14. Brooker D, Evans SC, Evans SB, *et al.* Evaluation of the implementation of the Meeting Centres Support Program in Italy, Poland, and the UK; exploration of the effects on people with dementia. *Int J Geriatr Psychiatry* 2018;33:883-892. doi: 10.1002/gps.4865.

15. Evans SB, Evans SC, Brooker D, *et al.* The impact of the implementation of the Dutch combined Meeting Centres Support Programme for family caregivers of people with dementia in Italy, Poland and UK. *Aging Ment Health* 2020;24:280-290. doi: 10.1080/13607863.2018.1544207

For peer review only

16. Lord K, Beresford-Dent J, Rapaport P, *et al.* Developing the New Interventions for independence in Dementia Study (NIDUS) theoretical model for supporting people to live well with dementia at home for longer: a systematic review of theoretical models and Randomised Controlled Trial evidence. *Soc Psychiatry Psychiatr Epidemiol* 2020;55:1-14. doi: 10.1007/s00127-019-01784-w

17. McDermott O, Charlesworth G, Hogervorst E, *et al.* Psychosocial interventions for people with dementia: a synthesis of systematic reviews. *Aging Ment Health* 2019;23:393–403. doi: 10.1080/13607863.2017.1423031

18. Van't Leven N, Prick A-EJC, Groenewoud JG, *et al.* Dyadic interventions for community-dwelling people with dementia and their family caregivers: a systematic review. *Int Psychogeriatr* 2013;25:1581-1603. doi:10.1017/S1041610213000860. pmid:http://www.ncbi.nlm.nih.gov/pubmed/23883489

19. Morton T, Atkinson T, Brooker D, *et al.* Sustainability of community-based interventions for people affected by dementia: a protocol for the SCI-Dem realist review. *BMJ Open* 2019;9:e032109. doi: 10.1136/bmjopen-2019-032109

20. Morton T, Wong G, Atkinson T, *et al.* Sustaining community-based interventions for people affected by dementia long term: the SCI-Dem realist review. *BMJ Open* 2021;11:e047789. doi: 10.1136/bmjopen-2020-047789

21. Dröes RM, Meiland FJM, de Lange J, *et al.* The meeting centres support programme; an effective way of supporting people with dementia who live at home and their carers. *Dementia-London* 2003;2:421-438.

22. Brooker D, Evans S, Droes RM. Framing outcomes of post-diagnostic psychosocial interventions in dementia: The Adaptation-Coping Model and Adjusting to Change. *Working with Older People* 2017;21(1). doi: 10.1108/WWOP-12-2016-0039

For peer review only

23. Dröes RM, Meiland FJM, Evans S, *et al.* Comparison of the adaptive implementation and evaluation of the Meeting Centers Support Program for people with dementia and their family carers in Europe; study protocol of the MEETINGDEM project. *BMC Geriatr* 2017;17:79.

24. Mangiaracina F, Chattat R, Farina E, *et al.* Not re-inventing the wheel: the adaptive implementation of the meeting centres support programme in four European countries. *Aging Ment Health* 2016;21:40–48. doi:10.1080/13607863.2016.1258540

25. The MeetingDem Network. *The MeetingDem Network website.* Amsterdam: VU University Medical Centre, 2018. https://www.meetingdem.eu/

26. Dröes RM, Meiland FJM, Schmitz MJ, *et al.* Effect of the Meeting Centres Support Program on informal carers of people with dementia: Results from a multi-centre study. *Aging Ment Health* 2006;10:112-24. doi: 10.1080/13607860500310682

27. Dröes RM, Meiland FJM, Schmitz MJ, *et al.* How do people with dementia and their carers evaluate the Meeting Centres Support Programme? *Non-Pharmacological Therapies in Dementia* 2011;2(1).

28. Lion KM, Szcześniak D, Bulińska K, *et al.* Does the Meeting Centre Support Programme decrease the experience of stigmatisation among people with cognitive deficits? *Aging Ment Health* 2021;25:160-169. doi:10.1080/13607863.2019.1683815

29. Mazurek J, Szcześniak D, Lion KM, *et al.* Does the Meeting Centres Support Programme reduce unmet care needs of community-dwelling older people with dementia? A controlled, 6-month follow-up Polish study. *Clin Interv Aging* 2019;14:113-122. https://doi.org/10.2147/CIA.S185683

30. Meiland FJM, Dröes RM, de Lange J, *et al.* (2010). Predictors of effective support in meeting centers for people with dementia and their carers. *Non-Pharmacological Therapies in Dementia* 2010;1:251-270.

For peer review only

31. Szcześniak D, Dröes R, Meiland F, *et al.* Does the community-based combined Meeting Center Support Programme (MCSP) make the pathway to day-care activities easier for people living with dementia? A comparison before and after implementation of MCSP in three European countries. *Int Psychogeriatr* 2018;30:1717-1734. doi:10.1017/S1041610217002885

32. Szcześniak D, Rymaszewska J, Saibene FL, *et al.* Meeting centres support programme highly appreciated by people with dementia and carers: a European cross-country evaluation. *Aging Ment Health* 2021;25:149-159. doi:10.1080/13607863.2019.1683814

33. Association for Dementia Studies. *ADS blog, November 18, 2021: Meeting Centres – what's the situation?* Worcester: University of Worcester, 2021. https://adsdementiablog.wordpress.com/2021/11/18/meeting-centres-whats-the-situation/#more-2075

34. Bould E, Ball A, Donoghue T, *et al. Dementia-Friendly Rural Communities Guide.* London: The Alzheimer's Society, 2018.

35. Pawson R, Tilley N. *Realistic Evaluation.* London: Sage Publications, 1997.

36. Checkland P. *Systems Thinking, Systems Practice.* New York: J. Wiley, 1999.

37. Dalkin S, Lhussier M, Williams L, *et al.* Exploring the use of Soft Systems Methodology with realist approaches: A novel way to map programme complexity and develop and refine programme theory. *Evaluation* 2018;24:84–97. doi.org/10.1177/1356389017749036

38. Williams L, Rycroft-Malone J, Burton CR, *et al.* Improving skills and care standards in the support workforce for older people: a realist synthesis of workforce development interventions. *BMJ Open* 2016;6:e011964. doi:10.1136/bmjopen-2016-011964

39. Hewitt G, Sims S, Harris R. The realist approach to evaluation research: An introduction. *International Journal of Therapy and Rehabilitation* 2012;19:250–9.

For peer review only

40. DEEP. DEEP: The UK Network of Dementia Voices website, 2021. Available: http://dementiavoices.org.uk

41. TIDE. TIDE: Together in Dementia Everyday website, 2021. Available: https://www.tide.uk.net/

42. Wong G, Westhorp G, Greenhalgh J, *et al.* Quality and reporting standards, resources, training materials and information for realist evaluation: the RAMESES II project. *Health Services and Delivery Research* 2017;5(28). https://doi.org/10.3310/hsdr05280

43. Brooker D. *The Essential Features of a Meeting Centre: UK Meeting Centres Support Programme (UKMCSP).* Worcester: University of Worcester, 2019. https://www.worcester.ac.uk/documents/Essential-Features-of-a-Meeting-Centre-booklet-November-2019-edition.pdf

44. Ministry of Housing, Communities & Local Government. *English Indices of Deprivation 2019.* London: Ministry of Housing, Communities & Local Governmen, 2019. Available: http://imd-bypostcode.opendatacommunities.org/imd/2019

45. Welsh Government. *Welsh Index of Multiple Deprivation 2019.* Cardiff: Welsh Government, 2019. Available: https://gov.wales/welsh-index-multiple-deprivation-full-indexupdate-ranks-2019

46. QSR International. *NVivo (Version 12).* Melbourne: QSR International Pty Ltd, 2018. https://www.qsrinternational.com/nvivo-qualitative-data-analysis-software/home

47. Salloum RG, Shenkman EA, Louviere JJ, *et al.* Application of discrete choice experiments to enhance stakeholder engagement as a strategy for advancing implementation: a systematic review. *Implement Sci* 2017;12:140. https://doi.org/10.1186/s13012-017-0675-8

48. de Bekker-Grob EW, Ryan M, Gerard, K. Discrete choice experiments in health economics: a review of the literature. *Health Econ* 2021;21:145-172. doi: 10.1002/hec.1697

For peer review only

49. Johnson FR, Lancsar E, Marshall D, *et al.* Constructing experimental designs for discrete-choice experiments: report of the ISPOR Conjoint Analysis Experimental Design Good Research Practices Task Force. *Value Health* 2013;16:3–13. doi:10.1016/j.jval.2012.08.2223

50. de Bekker-Grob EW, Donkers B, Jonker MF, *et al.* Sample size requirements for discrete-choice experiments in healthcare: a Practical Guide. *Patient* 2015;8:373–384. https://doi.org/10.1007/s40271-015-0118-z

51. Choice Metrics. *Ngene: Next generation stated choice experiments.* Sydney: Choice Metrics, 2018. http://www.choice-metrics.com

52. United Kingdom. *Mental Capacity Act 2005, c.9.* Available: https://www.legislation.gov.uk/ukpga/2005/9/contents

53. NHS Health Research Authority. *Consent and participant information guidance.* London: NHS Health Research Authority, 2021. Available: http://www.hra-decisiontools.org.uk/consent/

54. The Association for Dementia Studies. *UK Meeting Centres Support Programme (UK MCSP) webpage.* Worcester: University of Worcester, 2018. Available: https://www.worcester.ac.uk/about/academic-schools/school-of-allied-health-and-community/allied-health-research/association-for-dementiastudies/ads-research/uk-meeting-centres.aspx

55. Association for Dementia Studies. *Dementia research webpage.* Worcester: University of Worcester, 2022. Available: https://www.worcester.ac.uk/about/academic-schools/school-of-allied-health-and-community/allied-health-research/association-for-dementia-studies/ads-research/

For peer review only

Get Real with Meeting Centres: Overview Diagram

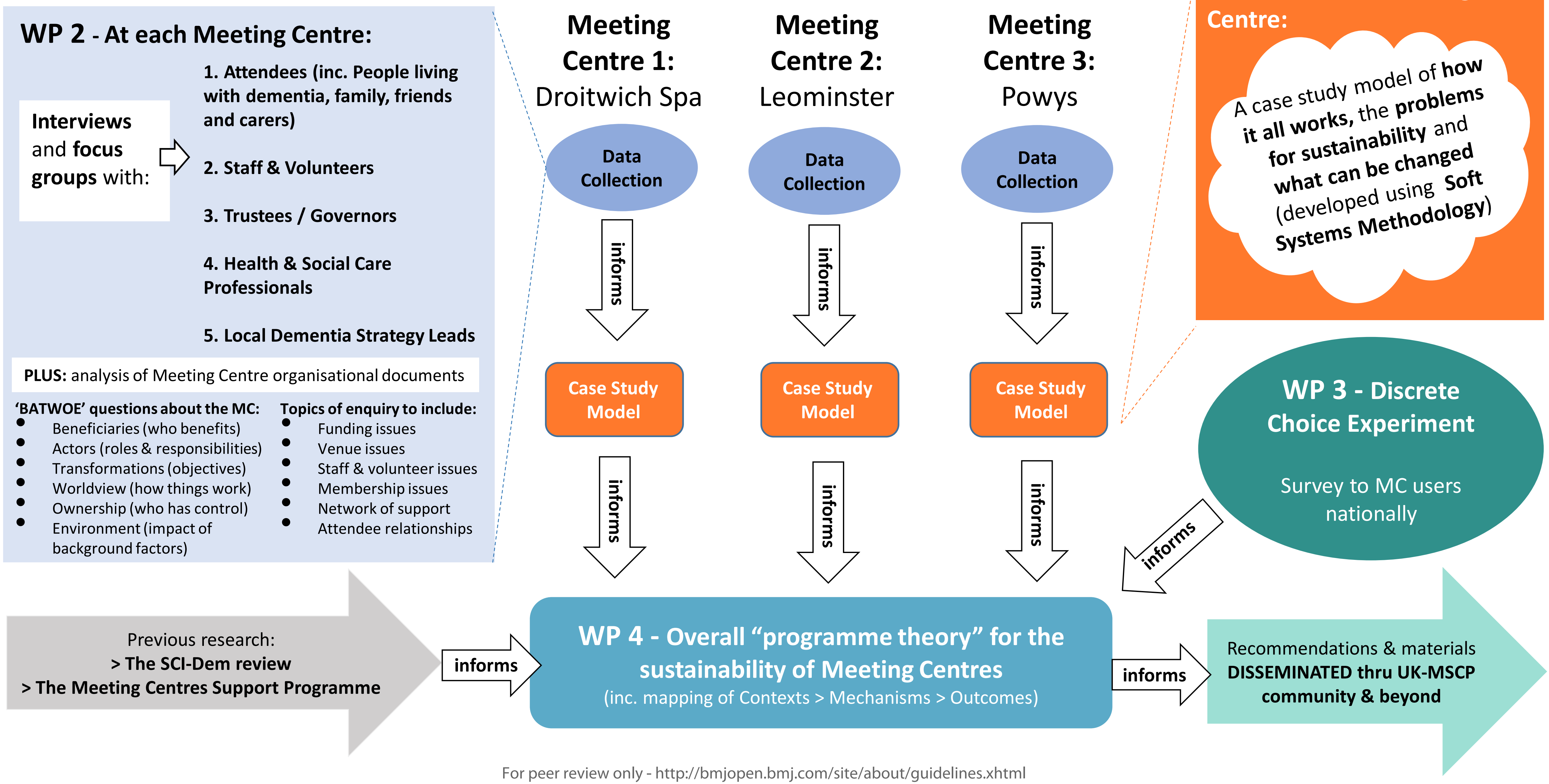

Note: Stakeholders (including patient and public representatives) at each MC - and within the UK-MCSP - will be consulted for input to each stage (WP 1)

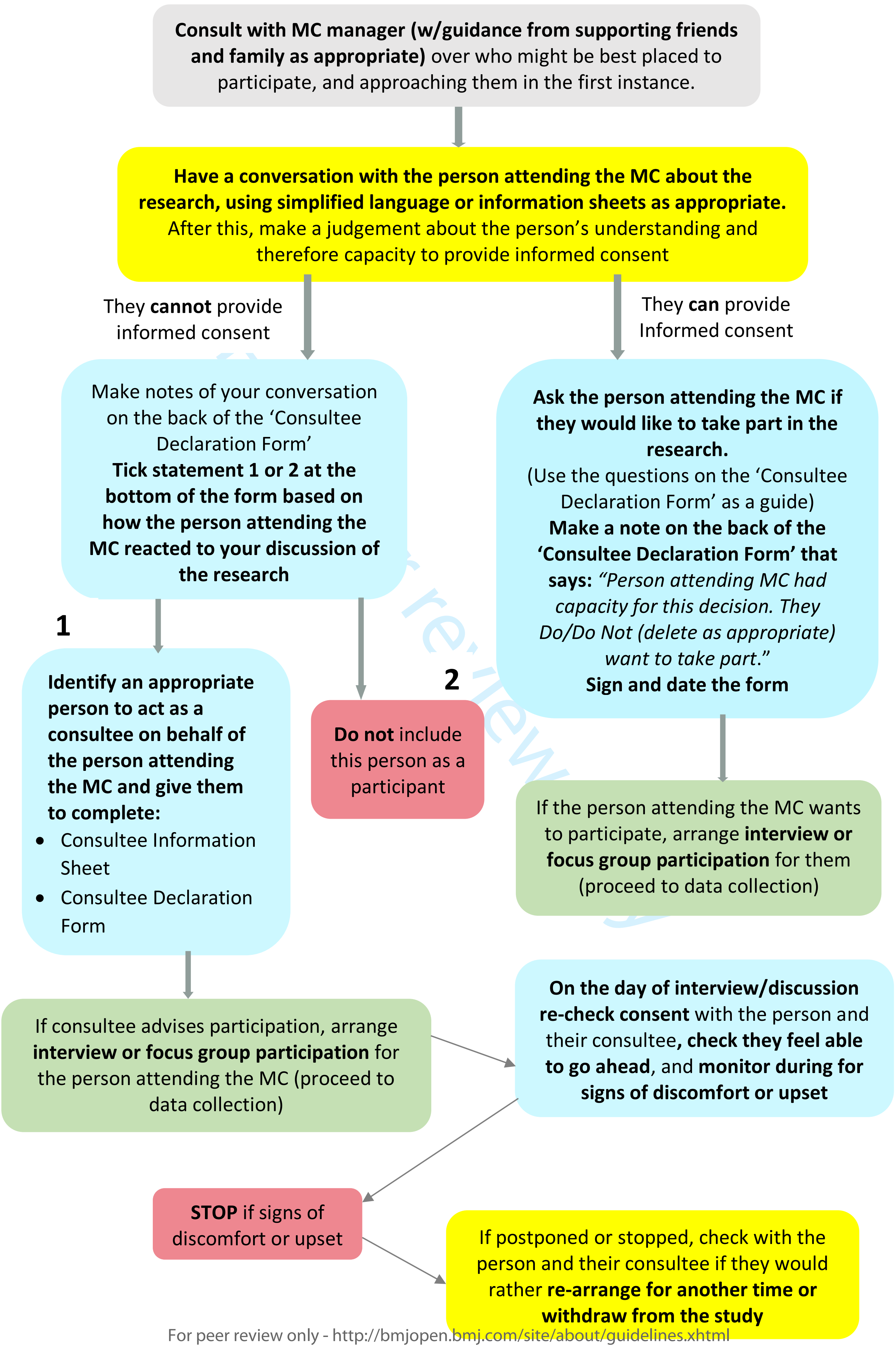
Consult with MC manager (w/guidance from supporting friends and family as appropriate) over who might be best placed to participate, and approaching them in the first instance.
Have a conversation with the person attending the MC about the research, using simplified language or information sheets as appropriate. After this, make a judgement about the person's understanding and therefore capacity to provide informed consent
They cannot provide informed consent
They can provide Informed consent
Make notes of your conversation on the back of the 'Consultee Declaration Form' Tick statement 1 or 2 at the bottom of the form based on how the person attending the MC reacted to your discussion of the research
Ask the person attending the MC if they would like to take part in the research. (Use the questions on the 'Consultee Declaration Form' as a guide) Make a note on the back of the 'Consultee Declaration Form' that says: "Person attending MC had capacity for this decision. They Do/Do Not (delete as appropriate) want to take part." Sign and date the form
1
2
Identify an appropriate person to act as a consultee on behalf of the person attending the MC and give them to complete:
• Consultee Information Sheet
• Consultee Declaration Form
Do not include this person as a participant
If the person attending the MC wants to participate, arrange interview or focus group participation for them (proceed to data collection)
If consultee advises participation, arrange interview or focus group participation for the person attending the MC (proceed to data collection)
On the day of interview/discussion re-check consent with the person and their consultee, check they feel able to go ahead, and monitor during for signs of discomfort or upset
STOP if signs of discomfort or upset
If postponed or stopped, check with the person and their consultee if they would rather re-arrange for another time or withdraw from the study

Interview/Group Discussion Schedule

For Meeting Centre personnel

Note: Which questions will be asked will be tailored to whether the interviewee has a strategic role or an operational role, in order to keep the number of questions concise and relevant, appropriate and understandable.

Introductions

Introduction of research staff and explanation of purpose:

"The purpose of this interview is to better understand how Meeting Centres have got up and running and how they have kept going over the past few years. We want to ask people for their knowledge and experience of how their Meeting Centre is run and what has made it a success, so that we can learn from it.

We will use what we learn to develop user-friendly guidance and materials to best help Meeting Centres survive and thrive in the long-term. We will also use this information to help policy makers and government understand what they can do to improve this situation. Our project will support the growth of more Meeting Centres to support people in desperate need. Thank you for agreeing to assist with this."

- Check provision of relevant study information sheet
- Confirm informed consent

Clarification of role:

- Ask for a description of the participant's role
- What do you do at the Meeting Centre? What are you responsible for?
- How did you get involved with the Meeting Centre?
- How long have you been involved with the Meeting Centre and has your role changed over time?

For peer review only - http://bmjopen.bmj.com/site/about/guidelines.xhtml

Get Real with Meeting Centres

1: Questions about who the Meeting Centre is aimed at helping:

- Who can become a member and who else benefits?
- How do you assess if a Meeting Centre is appropriate for someone?
- How do you find people? Do they come to you or do you approach them?
- What PR is done?
- What makes people want to come along and keeps people coming back?
- What can put people off coming or lead people to leave?
- Whose needs might not be met and why?
- To what extent do people supporting other attendees (e.g. friends/family) attend themselves?
- Do friends and family feel they also benefit? Are there challenges for them (e.g. arranging transport, fit with working hours)?
- Are all attendees happy with the programme of activities? What is most enjoyed and what could be better?
- How often do you reflect/consult on what is being provided and how well it meets the changing needs of attendees over time?

2: Questions about people's roles at the Meeting Centre:

- Who is responsible for what regarding the planning and running for the Meeting Centre?
- How many people are there in each of the different roles and is that the preferred number?
- What is their background and how did they come to be involved/how were they recruited?
- Have any left those roles any why?
- What training have personnel had and how did that happen?
- Are attendees involved in decision making?
- Are there any external people (e.g. referrers, community stakeholders) who play a key role in supporting the Meeting Centre? How did they come to be involved?
- Are there any external people (e.g. care professionals, practitioners, people who deliver a service) who come into the Meeting Centre?
- Are there any challenges or points of tension in working with external people?

3: Questions about how a Meeting Centre has developed:

- What were the most important things to have in place when setting up? What helped or hindered these things?
- What are the most important things to have in place now, and going forward? Are these the same things, or is the focus different now?
- How has the Meeting Centre and its work changed over time?
- What and links to the broader community have been developed, and how?
- How did the Meeting Centre respond to the COVID-19 pandemic? How has that affected how it might function in the future?

IRAS ID: 294636 Version: v0.3 09/06/21

4: Questions about how well things work.

- What things do you think are most key to keeping a Meeting Centre going long term?
- What are the key challenges in keeping going long term?
- What is good or not so good about how the Meeting Centre is run and its current situation?
- What opportunities are there for things to change?

5: Questions about who has the power to influence the success of a Meeting Centre, including:

- Whose support (person or organization) is essential to making a success of the Meeting Centre?
- What could influence their attitudes and decisions regarding the Meeting Centre?
- How could local or national authorities help or hinder the success of the Meeting Centre?
- Who in the Meeting Centre team could the Meeting Centre not continue without?
- How does the Meeting Centre meet its costs? What are the various sources of funding?
- How sustainable are these sources of funding? How difficult are they to come by?

6: Questions about the place and background circumstances around the Meeting Centre, including:

- Is the venue appropriate and working well for attendees and personnel?
- How does the local healthcare pathway fit with the Meeting Centre? Does it help, hinder or have no bearing on the Meeting Centre's success?
- How does the local/wider community view the Meeting Centre? Are there any misconceptions? Does it help, hinder or have no bearing on the Meeting Centre's success?
- How does the geography of the area (e.g. rural or urban) affect the Meeting Centre's success?
- How do people get to the Meeting Centre? What distance do they come from?
- How suitable is public transport? How available is community transport?

Interview closure

Is there anything else you'd like to tell us that you feel we should have asked you?

Many thanks for your time.

(Confirm any arrangements for checking accuracy of interpretation of views).

For peer review only - http://bmjopen.bmj.com/site/about/guidelines.xhtml

Interview/Group Discussion Schedule

For people who attend Meeting Centres

Note: Which questions are asked will be tailored to whether the interviewee is living with dementia or supporting someone who is, or if people are being interviewed as a pair, to ensure the questions are appropriate, understandable and kept concise and relevant. Concrete examples of people and organizations known to the participant will be used where possible, to make the questions less abstract.

Introductions

Introduction of research staff and explanation of purpose:

"The purpose of this interview is to better understand how Meeting Centres have got up and running and how they have kept going over the past few years. We want to ask people for their knowledge and experience of how their Meeting Centre is run and what has made it a success, so that we can learn from it.

We will use what we learn to develop user-friendly guidance and materials to best help Meeting Centres survive and thrive in the long-term. We will also use this information to help policy makers and government understand what they can do to improve this situation. Our project will support the growth of more Meeting Centres to support people in desperate need. Thank you for agreeing to assist with this."

- Check provision of relevant study information sheet
- Confirm who the participants are (and their relationship to each other if in a pair or group)
- Confirm informed consent or consultee declaration

For peer review only - http://bmjopen.bmj.com/site/about/guidelines.xhtml

Get Real with Meeting Centres

1: Questions about who the Meeting Centre is aimed at helping:

- What do you like about attending the Meeting Centre?
- How did you find out about the Meeting Centre?
- What might put people off coming to the Meeting Centre or want to stop coming along?
- What do you think of the activities at the Meeting Centre?
- Is there anything that make it difficult or challenging to attend?
- How do you feel about the cost?

2: Questions about people's roles at the Meeting Centre:

- Do you have a role in choosing what activities take place and deciding things?
- Do you have a role in helping out around the Meeting Centre?
- How involved in the Meeting Centre is your GP, or any care workers, nurses or other health professionals you know?
- Who provides transport to get there?

3: Questions about how a Meeting Centre has developed:

- How has the Meeting Centre changed over time?
- What happened with the Meeting Centre during lockdown and how did you feel about it?

4: Questions about how well things work:

- What do you like most and what is not so good about the Meeting Centre?
- What is most important to the Meeting Centre's success, and what can get in the way?

For peer review only - http://bmjopen.bmj.com/site/about/guidelines.xhtml

Get Real with Meeting Centres

5: Questions about who has the power to influence the success of a Meeting Centre:

- Do you know if authorities like the County Council, the NHS, the Government or any relevant big national charities help or hinder the success of the Meeting Centre?
- Do you feel like you are in control of what happens at the Meeting Centre?

6: Questions about the place and background circumstances around the Meeting Centre:

- Is the venue appropriate and easy to get around?
- Do you know how your GPs, care workers etc. view the Meeting Centre?
- Do you know how the local community views the Meeting Centre?
- How easy is it for you to get to the Meeting Centre and what is transport like?

Interview closure

Is there anything else you'd like to tell us that you feel we should have asked you?

Many thanks for your time.

(Confirm any arrangements for checking accuracy of interpretation of views).

For peer review only - http://bmjopen.bmj.com/site/about/guidelines.xhtml

Interview/Group Discussion Schedule

For external partners & stakeholders to Meeting Centres

Note: Which questions will be asked will be tailored to suit the relationship the external interviewee has with the Meeting Centre – e.g. whether a direct referrer or someone otherwise involved with the local dementia care pathway – to keep the number of questions concise and relevant, appropriate and understandable.

For peer review only

Introductions

Introduction of research staff and explanation of purpose:

"The purpose of this interview is to better understand how Meeting Centres have got up and running and how they have kept going over the past few years. We want to ask people for their knowledge and experience of how their Meeting Centre is run and what has made it a success, so that we can learn from it.

We will use what we learn to develop user-friendly guidance and materials to best help Meeting Centres survive and thrive in the long-term. We will also use this information to help policy makers and government understand what they can do to improve this situation. Our project will support the growth of more Meeting Centres to support people in desperate need. Thank you for agreeing to assist with this."

- Check provision of relevant study information sheet
- Confirm informed consent
- Ask for a description of the participant's role and involvement with the Meeting Centre

For peer review only - http://bmjopen.bmj.com/site/about/guidelines.xhtml

Get Real with Meeting Centres

1: Questions about who the Meeting Centre is aimed at helping:

- What is your understanding of who can become a member and who else benefits?
- Do you have access to Meeting Centre advertising/info materials? Are they effective?
- How does the Meeting Centre reach the right people?
- If you are involved in referring, how do you assess if a Meeting Centre is appropriate for someone? What level of consultation is carried out with people and families?
- What factors do you think most make Meeting Centres attractive to people? What do people value most?
- What could put people off going to a Meeting Centre?
- To what extent do you think the Meeting Centre meets people's needs?
- What are the main benefits or challenges for the people supporting attendees (such as family carers)?

2: Questions about people's roles at the Meeting Centre:

- What role do you see yourself as playing in the Meeting Centre and how it functions?
- How did you get involved? Has your involvement changed over time?
- How important is the Meeting Centre, or initiatives like it, to your work?
- How important do you think your support is to the Meeting Centre?
- Who else (people or organizations) play a role in supporting the Meeting Centre? What do they do and how did they come to be involved?
- Are there any challenges or points of tension in working with the Meeting Centre?

3: Questions about how a Meeting Centre has developed:

- What was most important in helping or hindering the setting up of the Meeting Centre?
- What are the most important things to have in place now, and going forward? Are these the same things, or is the focus different now?
- How has the Meeting Centre and its work changed over time?
- Did you continue to have involvement with the Meeting Centre during lockdown? If so, how?
- How has the pandemic impacted upon the Meeting Centre, now and going forward?

4: Questions about how well things work.

- What do you think is most key to keeping a Meeting Centre going long term? What are the main challenges?
- What is good about how the Meeting Centre is run and its current situation?

IRAS ID: 294636 Version: v0.3 09/06/21

For peer review only

- What is not so good about how the Meeting Centre is run and its current situation?
- What opportunities are there for things to change – what should be done and what can be done?

5: Questions about who has the power to influence the success of a Meeting Centre, including:

- Whose support (person or organization) is essential to making a success of the Meeting Centre?
- Does anyone (person or organization) have the power to stop the Meeting Centre from being able to run?
- What could influence their attitudes and decisions regarding the Meeting Centre?
- How could local authorities help or hinder the success of the Meeting Centre? How could national authorities help?
- Who in the Meeting Centre team could the Meeting Centre not continue without?

6: Questions about the place and background circumstances around the Meeting Centre, including:

- Do you know if the venue is appropriate and working well for everyone?
- How does the local healthcare pathway fit with the Meeting Centre? Does it help, hinder or have no bearing on the Meeting Centre's success? How could things be done differently?
- How does the local/wider community view the Meeting Centre? Does it help, hinder or have no bearing on the Meeting Centre's success?
- How does the geography of the area (e.g. rural or urban) affect the Meeting Centre's success?
- How do people get to the Meeting Centre? What distance do they come from?
- How suitable is public transport? How available is community transport?

For peer review only

Interview closure

Is there anything else you'd like to tell us that you feel we should have asked you?

Many thanks for your time.

(Confirm any arrangements for checking accuracy of interpretation of views).

For peer review only - http://bmjopen.bmj.com/site/about/guidelines.xhtml

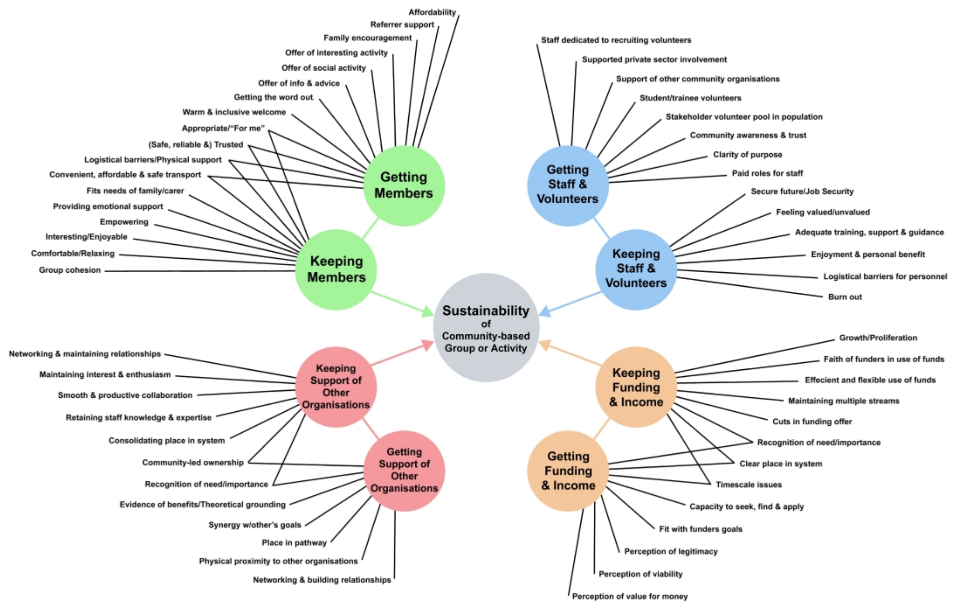

SCI-Dem overview: Factors affecting the sustainability of community-based groups and activities

600x424mm (300 x 300 DPI)

Information Sheet for people involved in Meeting Centres

Research Study Title - Get Real with Meeting Centres: A Realist Evaluation

You have received this information sheet because we would like you to take part in our research study. The following information explains what the research study is and what it would mean for you if you decide to be involved. Please take time to read over it and discuss the information with other people before you make your decision.

What is the purpose of the study?

This study will look at how three Meeting Centres have got up and running and how they have kept going over the past few years. One of these is the Meeting Centre that you have involvement with. Many more Meeting Centres are now starting, so it is important we learn more about what has helped to make Meeting Centres successful. We have developed this study so that we can learn from it and develop tips and guidance for others. We will take all the information people tell us and map out how each Meeting Centre works, how it has worked over the past few years, and how best to plan for the future, going forward.

Why am I being asked to take part?

We are asking you to take part as you have direct experience of being involved in a Meeting Centre. We want to ask people for their knowledge and experience of how their Meeting Centre is run and what has made it a success.

Do I have to take part?

No, you do not have to take part in the study if you do not want to. You will be given a week to decide. If you decide against it, it will have no impact on your involvement with your Meeting Centre. You can change your mind about being involved in the study at any time and without giving a reason. Information about people who have not consented to be involved will not be recorded as part of the study. You also have the right to withdraw your data after participation, in which case it will be destroyed on request.

For peer review only
For peer review only - http://bmjopen.bmj.com/site/about/guidelines.xhtml

If you decide that you would like to take part in this research study, you will first be asked to sign a consent form and then we will arrange with you the next steps of taking part.

What will happen if I wish to take part?

You will have the opportunity to be involved in informal interviews and group discussions over the coming months. You can make the decision on how much involvement you would like to have and which aspects you would like to get involved with.

The research study and COVID

We hope to be able to hold interviews and group discussions in person, but this is dependent on the COVID-19 restrictions. We are hopeful that in the near future it will be safe for us to visit each Meeting Centre. If so, we will abide by all current local and national guidance on what is safe to do when visiting. If this is not possible, then we will carry out our interviews and discussion groups by online meetings (such as via Zoom) or by telephone and email.

Even if we can attend Meeting Centres, you can let us know whether you would like to be involved face-to-face or via an online method such as Zoom.

Will my involvement be kept confidential?

We keep the specific feedback in individual interviews confidential, but we will state our general findings in our final report. However, if something is disclosed during interviews or discussions that could impact upon the safety of yourself or others, confidentially may be breached if necessary to ensure safeguarding. Any feedback that we get from you will be checked for accuracy and anonymised through the removal of people’s names and other personal information. When we write up our findings, we will use quotes from people we have spoken with. You will be asked your preferences on whether or not you would like your name to be referenced by the quote. When the results of the study are presented, we will not use the name of the Meeting Centres involved in the study but will refer to them by region.

For peer review only - http://bmjopen.bmj.com/site/about/guidelines.xhtml

What are the possible disadvantages to taking part?

There are no obvious disadvantages to you taking part in the research. We are interested to hear your feedback.

What are the possible benefits of taking part?

By taking part in this study you will be providing valuable information that we can learn from, to help other Meeting Centres to be a success. You get to share your opinions on Meeting Centres, what you think of them and how they might improve to help those who attend them. You may also help your own Meeting Centre, by helping us to understand how it works and what else we can do to help it in the future.

We hope that you will find taking part in interviews and discussions with us interesting and enjoyable.

What will happen to the results of the study?

The results will give us the information to make recommendations to other Meeting Centres across the UK. The results of the study will be written up in a final report and shared with the National Institute of Health Research, who are funding the study. The results will also be used to write articles, reports or include in presentations. We will make sure our results are made available to share with you once the study is complete.

Who is organising or funding the study?

This study is being led by the Association for Dementia Studies at the University of Worcester. It is funded by the **National Institute for Health Research (NIHR)**

Who has reviewed the study?

This research study has been reviewed and approved by XXX. A research ethics committee is a group of people who review research studies to ensure they protect the dignity, rights, safety and well-being of research participants and researchers.

For peer review only - http://bmjopen.bmj.com/site/about/guidelines.xhtml

For any further information please contact:

Shirley Evans – PI

s.evans@worc.ac.uk

Association for Dementia Studies

University of Worcester

Henwick Grove, Worcester, WR2 6AJ

Thomas Morton – Research Associate

t.morton@worc.ac.uk

Association for Dementia Studies

University of Worcester

Henwick Grove, Worcester, WR2 6AJ

For peer review only

Thank you for taking time to read this information sheet.

For peer review only - http://bmjopen.bmj.com/site/about/guidelines.xhtml

Information Sheet (short version)

The Get Real with Meeting Centres Study

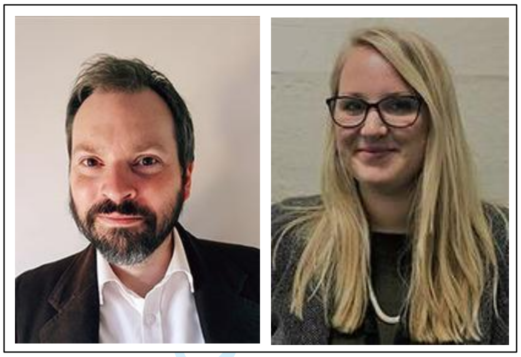

The Researchers:
Thomas (left) and Faith (Right)

For peer review only

What we are doing

We are doing a research study at the Meeting Centre that you go to because we want to find out more about Meeting Centres and what makes them successful. Meeting Centres are really popular and there are lots of new ones that are being set up. We hope that we can find out how to make them the best that they can be.

We would like you to talk to us about the Meeting Centre that you go to and your feelings about it.

How you can take part

Tell someone at your Meeting Centre if you would like to take part. You will be invited to take part in things like **group discussions** and **one-to-one conversations,** to talk to us about the Meeting Centre you go to.

Group Discussions

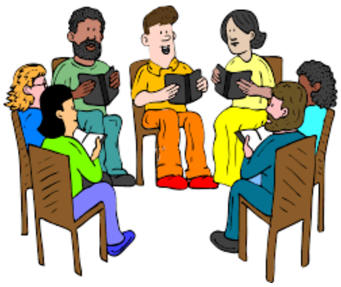

One-to-one Conversations

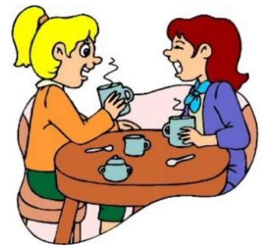

For peer review only - http://bmjopen.bmj.com/site/about/guidelines.xhtml

What will happen next?

We will ask you to sign a consent form saying that you want to take part. We will then arrange the next steps, like when you would like to talk with us and how.

Will my information be confidential?

The information you tell us will be checked for accuracy and anonymised by removing anyone's names and any other personal information. If we quote something you have said, we will ask you if you wish for your name to be used before we share it with anyone. Confidentiality will only be broken if there is a danger to the safety of yourself or someone else.

The Meeting Centre you go to will not be named in any reports, but we will describe it by the region it is located in.

What if I have a concern?

If you have any worries or questions please talk to us or someone else that you feel comfortable with. We are happy to arrange a time to come and talk with you and answer any questions. **For more info, contact:**

Thomas Morton

University of Worcester, Henwick Grove, Worcester, WR2 6AJ

Tel: 01905 542326 **Email: t.morton@worc.ac.uk**

Please remember:

You do not have to take part in this research study. It is your decision if you want to stop taking part in the research at any point and you do not have to give a reason why. If you do not decide to take part but then change your mind, that is fine as well. You can join in when you wish.

Whatever you decide, it will not affect your involvement with the Meeting Centre you go to.

This research has been approved by XXX

Thank you for taking time to read this information sheet.

For peer review only - http://bmjopen.bmj.com/site/about/guidelines.xhtml

Information Sheet for external partners working with and supporting Meeting Centres

External partners may include health, social care and third sector professionals, community-based partners and those involved with the local dementia pathway/DFC

Research Study Title - Get Real with Meeting Centres: A Realist Evaluation

You have received this information sheet because we would like you to take part in our research study. The following information explains what the research study is and what it would mean for you if you decide to get involved. Please take time to read over it and discuss the information.

What is the purpose of the study?

This study will look at how three Meeting Centres have got up and running and how they have kept going over the past few years. One of these is the Meeting Centre that you work with/ support. Many more Meeting Centres are now starting, so it is important we learn more about what has helped to make Meeting Centres successful. We have developed this study so that we can learn from it and develop tips and guidance for others. We will take all the information people tell us and map out how each Meeting Centre works, how it has worked over the past few years, and how best to plan for the future, going forward.

Why am I being asked to take part?

We are asking you to take part as you have direct experience of working with/supporting a Meeting Centre. We want to ask people for their knowledge and experience of how they think the Meeting Centre is run and what has made it a success.

Do I have to take part?

No, you do not have to take part in the study. You will be given a week to decide. If you decide against it, it will have no impact on your relationship with the Meeting Centre you currently work with/support. You can change your mind at any time and without giving a reason.

For peer review only - http://bmjopen.bmj.com/site/about/guidelines.xhtml

Information about people who have not consented to be involved will not be recorded as part of the study. You also have the right to withdraw your data after participation by which it will be destroyed on request.

If you decide that you would like to take part in this research study, you will first be asked to sign a consent form. We will then discuss with you the next steps of taking part.

What will happen if I wish to take part?

You will have the opportunity to be involved in informal interviews and group discussions over the coming months. You can make the decision on how much involvement you would like to have and which aspects you would like to get involved with.

The research study and COVID

We hope to be able to hold interviews and group discussions in person, but this is dependent on the COVID-19 restrictions. We are hopeful that in the near future it will be safe for us to visit each Meeting Centre. If so, we will abide by all current local and national guidance on what is safe to do when visiting. If this is not possible, then we will carry out our interviews and discussion group by online meetings (such as via Zoom) or by telephone and email.

Even if we can attend Meeting Centres, you can let us know whether you would like to be involved face-to-face or via an online method such as Zoom.

Will my involvement be kept confidential?

We keep the specific feedback in individual interviews confidential, but we will state our general findings in our final report. However, if something is disclosed during interviews or discussions that could impact upon the safety of yourself or others, confidentially may be breached if necessary to ensure safeguarding. Any feedback that we get from you will be checked for accuracy and anonymised through the removal of people's names and other personal information. When we write up our findings, we will use quotes from people we have spoken with. You will be asked your preferences on whether or not you would like your name

For peer review only - http://bmjopen.bmj.com/site/about/guidelines.xhtml

to be referenced by the quote. When the results of the study are presented, we will not use the name of the Meeting Centres involved in the study but will refer to them by region.

What are the possible disadvantages to taking part?

There are no obvious disadvantages to you taking part in the research. We are interested to hear your feedback.

What are the possible benefits of taking part?

By taking part in this study you will be providing valuable information that we can learn from to help other Meeting Centres to be a success. You get to share your opinions on Meeting Centres, what you think of them and how they might improve to help those who attend them. You may also help the Meeting Centre you work with/support, by helping us to understand how it works and what else we can do to help it in the future.

What will happen to the results of the study?

The results will give us the information to make recommendations to other Meeting Centres across the UK. They will be written up in a final report and shared with the National Institute of Health Research, who are funding the study. The results will also be used to write articles, reports or be included in presentations. We will make sure our results are made available to share with you once the study is complete.

Who is organising or funding the study?

This study is being led by the Association for Dementia Studies at the University of Worcester. It is funded by the **National Institute for Health Research (NIHR)**

Who has reviewed the study?

This research study has been reviewed and approved by XXX. A research ethics committee is a group of people who review research studies to ensure they protect the dignity, rights, safety and well-being of research participants and researchers.

For peer review only - http://bmjopen.bmj.com/site/about/guidelines.xhtml

For any further information please contact:

Shirley Evans – PI

s.evans@worc.ac.uk

Association for Dementia Studies

University of Worcester

Henwick Grove, Worcester, WR2 6AJ

Thomas Morton – Research Associate

t.morton@worc.ac.uk

Association for Dementia Studies

University of Worcester

Henwick Grove, Worcester, WR2 6AJ

Thank you for taking time to read this information sheet.

For peer review only - http://bmjopen.bmj.com/site/about/guidelines.xhtml

Information Sheet for Personal Consultees

A personal consultee is someone who advises on the preferences and past wishes of a person who is no longer able to give informed consent.

Research Study Title - Get Real with Meeting Centres: A Realist Evaluation

You have received this information sheet because we are inviting your relative/friend/the person you represent to take part in our research study. The following information explains what the research study is and what it would mean for the person you are acting as a personal consultee on behalf of to get involved. Please take time to read over it and discuss the information with other people before you make your decision.

What is the purpose of the study?

This study will look at how three Meeting Centres have got up and running and how they have kept going over the past few years. One of these is the Meeting Centre that your relative/friend /the person you represent goes to. Many more Meeting Centres are now starting, so it is important we learn more about what has helped to make Meeting Centres successful. We have developed this study so that we can learn from it and develop tips and guidance for others. We will take all the information people tell us and map out how each Meeting Centre works, how it has worked over the past few years, and how best to plan for the future, going forward.

Why am I being asked about this study?

We would like to involve your relative/friend/the person you represent in this study. Due to the fact that your relative/friend/the person you represent may be unable to decide for him/ herself, we are asking you to inform us on their behalf. We are asking them to take part as they have direct experience of going to a Meeting Centre. We want to ask people for their knowledge and experience of how they think the Meeting Centre is run and what has made it a success.

For peer review only - http://bmjopen.bmj.com/site/about/guidelines.xhtml

Does my relative/friend/the person I represent have to take part?

No. We are asking for your advice about whether they might object to taking part. You will be given a week to decide. They do not have to take part and their experience of going to their Meeting Centre will not change if they do not take part.

You may want to think about whether they have expressed an interest in taking part in research before. If you think they would **not object** to taking part, then please complete the **Consultee Declaration Form** and return it to the researchers (or a member of Meeting Centre staff to pass on to them).

If you feel that the person would not like to take part, it will have no impact on yours or their relationship with the Meeting Centre that they currently go to. You can change your mind at any time, without giving a reason. Information about people who have not consented to be involved will not be recorded as part of the study. You also have the right to withdraw your data after participation, in which case it will be destroyed on request.

If you think your relative/friend/the person you represent would like to take part in this research, we will arrange the next steps of taking part with both of you.

What will happen if I advise they would wish to take part?

If you say yes to your relative/friend/the person you represent taking part the following things will happen:

1. After reading this information sheet, you will need to complete a consultee declaration form as the person's consultee.
2. A member of the research team will then explain the study to you and your relative/ friend/the person you represent and invite them to take part in participating in an informal interview or a group discussion around the Meeting Centre that they go to.
3. You and your relative/friend/the person you represent can make the decision on how much involvement they would like to have and which aspects they would like to get involved with.

For peer review only - http://bmjopen.bmj.com/site/about/guidelines.xhtml

4. The researcher will take some notes of your relative/friend/the person you represent's feedback.
5. The research team will then look at this feedback and integrate it into their findings.

The research study and COVID

We hope to be able to hold interviews and group discussions in person, but this is dependent on the COVID-19 restrictions. We are hopeful that in the near future it will be safe for us to visit each Meeting Centre. If so, we will abide by all current local and national guidance on what is safe to do when visiting. If this is not possible, then we will carry out our interviews and discussion group by online meetings (such as via Zoom) or by telephone and email.

Even if we can attend Meeting Centres, you can let us know whether you would like to be involved face-to-face or via an online method such as Zoom.

Will the involvement of the person I represent be kept confidential?

We keep the specific feedback in individual interviews confidential, but we will state our general findings in our final report. However, if something is disclosed during interviews or discussions that could impact upon the safety of your relative/friend/the person you represent or others, confidentially may be breached if necessary to ensure safeguarding. Any feedback that we get from you will be checked for accuracy and anonymised through the removal of people's names and other personal information. When we write up our findings, we will use quotes from people we have spoken with, but these will be attributed to a pseudonym to retain anonymity in the case of people who are unable to give informed consent. When the results of the study are presented, we will not use the name of the Meeting Centres involved in the study but will refer to them by region.

What are the possible disadvantages to taking part?

There are no obvious disadvantages to your relative/friend/the person you represent taking part in the research. We are interested to hear their feedback.

For peer review only - http://bmjopen.bmj.com/site/about/guidelines.xhtml

What are the possible benefits of taking part?

By taking part in this study you will be providing valuable information that we can learn from to help other Meeting Centres to be a success. Your relative/friend/the person you represent will be able to have their say on Meeting Centres, what they think of them and how they might improve to help those who attend them. By being involved, they may also help the Meeting Centre that they go to by helping us to understand how it works and what else we can do to help it in the future.

What will happen to the results of the study?

The results will give us the information to make recommendations to other Meeting Centres across the UK. The results of the study will be written up in a final report and shared with the National Institute of Health Research, who are funding the study. The results will also be used to write articles, reports or be included in presentations. We will make sure our results are made available to share with you once the study is complete.

Who is organising or funding the study?

This study is being led by the Association for Dementia Studies at the University of Worcester. It is funded by the **National Institute for Health Research (NIHR)**

Who has reviewed the study?

This research study has been reviewed and approved by XXX. A research ethics committee is a group of people who review research studies to ensure they protect the dignity, rights, safety and well-being of research participants and researchers.

For peer review only - http://bmjopen.bmj.com/site/about/guidelines.xhtml

For any further information please contact:

Shirley Evans – PI

s.evans@worc.ac.uk

Association for Dementia Studies

University of Worcester

Henwick Grove, Worcester, WR2 6AJ

Thomas Morton – Research Associate

t.morton@worc.ac.uk

Association for Dementia Studies

University of Worcester

Henwick Grove, Worcester, WR2 6AJ

Thank you for taking time to read this information sheet.

For peer review only

For peer review only - http://bmjopen.bmj.com/site/about/guidelines.xhtml

IRAS ID: 294636
Centre Number:
Study Number:
Participant Identification Number:

University of Worcester
Association for Dementia Studies

Research Study Consent Form

Title of Project: Get Real with Meeting Centres: A Realist Evaluation

Name of Researcher:

Please initial box

1. I confirm that I have read the information sheet dated 09/06/21 (version 0.4) for the above study. I have had the opportunity to consider the information, ask questions and have had these answered satisfactorily. ☐

2. I understand that my participation is voluntary and that I am free to withdraw at any time without giving any reason, without my involvement in the Meeting Centre or legal rights being affected. ☐

3. I understand that the information collected about me will be used to support other research in the future and may be shared anonymously with other researchers. ☐

4. I understand that an audio recording may be made of interviews and/or discussions that I take part in as part of this research, and do not object to this. ☐

5. I understand that quotes from what I say in my interviews and/or discussions may be used when the findings of this study are written up. ☐

   With regards to the above, please tick one of the below:

   I would like my name to be changed on any quotes used from me. ☐

   I am happy my name to be used on any quotes from me. ☐

Please turn over…

For peer review only - http://bmjopen.bmj.com/site/about/guidelines.xhtml

6. I agree to take part in the above study. ☐

7. I know who to contact if I have any concerns about this research. ☐

Name of participant: ____________ Date: ____________ Signature: ____________

Name of person taking consent: ____________ Date: ____________ Signature: ____________

For peer review only

For peer review only - http://bmjopen.bmj.com/site/about/guidelines.xhtml

IRAS ID: 294636

Centre Number:

Study Number:

Participant Identification Number for this study:

University of Worcester
Association for Dementia Studies

Consultee Declaration Form

Title of Project: Get Real with Meeting Centres: A Realist Evaluation

Name of Researcher:

Please initial box

1. I …………………….…….. have been consulted about …………………….…….’s participation in this research project. I have had the opportunity to ask questions about the study and understand what is involved. ☐

2. I understand that I do not have to act as consultee and I can change my mind at any time. ☐

3. In my opinion he/she would have no objection to taking part in the above study. ☐

4. I understand I can request he/she is withdrawn from the study at any time, without giving a reason and without his/her involvement in the Meeting Centre being affected. ☐

5. I understand that the information collected about him/her will be used to support other research in the future, and may be shared anonymously with other researchers. ☐

6. I understand that an audio recording may be made of interviews and/or discussions that he/she takes part in as part of this research, and in my opinion he/she would not object to this. ☐

7. I understand that quotes from what he/she says in interviews and/or discussions may be used when the findings of this study are written up, with names changed to preserve anonymity, and in my opinion he/she would not object to this. ☐

Please turn over…

For peer review only - http://bmjopen.bmj.com/site/about/guidelines.xhtml

8. I know who to contact if I have any concerns about this research. ☐

Name of Consultee: Date: Signature:

Relationship to participant:

Person undertaking consultation:

Name Date Signature

To be completed by researcher prior to seeking consultee declaration (tick one),

☐ 1. Potential participant showed signs of a positive or interested attitude when going through the participant information

☐ 2. Potential participant showed signs of a negative or anxious attitude toward the research or taking part in it when going through the information

For peer review only

For peer review only - http://bmjopen.bmj.com/site/about/guidelines.xhtml

RAMESES II reporting standards for realist evaluations: checklist

Title: The sustainability of locally-driven centres for those affected by dementia: A protocol for the Get Real with Meeting Centres realist evaluation

TITLE			Reported in document Y/N/Unclear/ Not applicable	Page(s) in document	Comment
**1**		In the title, identify the document as a realist evaluation.	Y	1,2	
**SUMMARY OR ABSTRACT**					
**2**		Journal articles will usually require an abstract, while reports and other forms of publication will usually benefit from a short summary. The abstract or summary should include brief details on: the policy, programme or initiative under evaluation; programme setting; purpose of the evaluation; evaluation question(s) and/or objective(s); evaluation strategy; data collection, documentation and analysis methods; key findings and conclusions.<br><br>Where journals require it and the nature of the study is appropriate, brief details of respondents to the evaluation and recruitment and sampling processes may also be included.<br><br>Sufficient detail should be provided to identify that a realist approach was used and that realist programme theory was developed and/or refined.	Y	2-3	Abstract presented in study protocol format

For peer review only

**INTRODUCTION**					
**3**	Rationale for evaluation	Explain the purpose of the evaluation and the implications for its focus and design.	Y	4-6	
**4**	Programme theory	Describe the initial programme theory (or theories) that underpin the programme, policy or initiative.	Y	4, 6, 10, 11. Supplementary file 2.	As this is a study protocol these will be developed, however programme theory from the previous SCI-Dem study will act as a preliminary framework, referenced in the Introduction (page 4) and Methods (pages 6 and 10) sections and described in Supplementary file 2. MCs are also guided by the Adaptation Coping Model, referenced on pages 4 and 11.
**5**	Evaluation questions, objectives and focus	State the evaluation question(s) and specify the objectives for the evaluation. Describe whether and how the programme theory was used to define the scope and focus of the evaluation.	Y	6-7	At the start of the Methods section.
**6**	Ethical approval	State whether the realist evaluation required and has gained ethical approval from the relevant authorities, providing details as appropriate. If ethical approval was deemed unnecessary, explain why.	Y	13	As this is a study protocol, this is dealt with in the Ethics and Dissemination section.
**METHODS**					

For peer review only

**7**	Rationale for using realist evaluation	Explain why a realist evaluation approach was chosen and (if relevant) adapted.	Y	8	
**8**	Environment surrounding the evaluation	Describe the environment in which the evaluation took place.	Y	9	
**9**	Describe the programme policy, initiative or product evaluated	Provide relevant details on the programme, policy or initiative evaluated.	Y	5,7,9	
**10**	Describe and justify the evaluation design	A description and justification of the evaluation design (i.e. the account of what was planned, done and why) should be included, at least in summary form or as an appendix, in the document which presents the main findings. If this is not done, the omission should be justified and a reference or link to the evaluation design given. It may also be useful to publish or make freely available (e.g. online on a website) any original evaluation design document or protocol, where they exist.	Y	7-13	
**11**	Data collection methods	Describe and justify the data collection methods – which ones were used, why and how they fed into developing, supporting, refuting or refining programme theory.<br><br>Provide details of the steps taken to enhance the trustworthiness of data collection and documentation.	Y	9-13	

For peer review only

**12**	Recruitment process and sampling strategy	Describe how respondents to the evaluation were recruited or engaged and how the sample contributed to the development, support, refutation or refinement of programme theory.	Y	7-15	Recruitment and sampling is covered both in the Methods, and Ethics and Dissemination sections.
**13**	Data analysis	Describe in detail how data were analysed. This section should include information on the constructs that were identified, the process of analysis, how the programme theory was further developed, supported, refuted and refined, and (where relevant) how analysis changed as the evaluation unfolded.	Y	10-13	As this is a study protocol, this covers plans for data analysis.
**RESULTS**					
**14**	Details of participants	Report (if applicable) who took part in the evaluation, the details of the data they provided and how the data was used to develop, support, refute or refine programme theory.	Not applicable		Study protocol
**15**	Main findings	Present the key findings, linking them to contexts, mechanisms and outcome configurations. Show how they were used to further develop, test or refine the programme theory.	Not applicable		Study protocol
**DISCUSSION**					
**16**	Summary of findings	Summarise the main findings with attention to the evaluation questions, purpose of the evaluation, programme theory and intended audience.	Not applicable		Study protocol

For peer review only

**17**	Strengths, limitations and future directions	Discuss both the strengths of the evaluation and its limitations. These should include (but need not be limited to): (1) consideration of all the steps in the evaluation processes; and (2) comment on the adequacy, trustworthiness and value of the explanatory insights which emerged.<br><br>In many evaluations, there will be an expectation to provide guidance on future directions for the programme, policy or initiative, its implementation and/or design. The particular implications arising from the realist nature of the findings should be reflected in these discussions.	Not applicable		Study protocol
**18**	Comparison with existing literature	Where appropriate, compare and contrast the evaluation's findings with the existing literature on similar programmes, policies or initiatives.	Not applicable		Study protocol
**19**	Conclusion and recommendations	List the main conclusions that are justified by the analyses of the data. If appropriate, offer recommendations consistent with a realist approach.	Not applicable		Study protocol
**20**	Funding and conflict of interest	State the funding source (if any) for the evaluation, the role played by the funder (if any) and any conflicts of interests of the evaluators.	Y	13,18	

Adapted from table 1 in: Wong G, Westhorp G, Manzano A, et al. RAMESES II reporting standards for realist evaluations. BMC Med 2016;14:96

For peer review only

BMJ Open

The sustainability of locally-driven centres for those affected by dementia: A protocol for the Get Real with Meeting Centres realist evaluation

Journal:	*BMJ Open*
Manuscript ID	bmjopen-2022-062697.R1
Article Type:	Protocol
Date Submitted by the Author:	14-Mar-2022
Complete List of Authors:	Morton, Thomas; University of Worcester, Association for Dementia Studies<br>Evans, Shirley; University of Worcester, Association for Dementia Studies<br>Brooker, Dawn; University of Worcester, Association for Dementia Studies<br>Williamson, Tracey; University of Worcester, Association for Dementia Studies<br>Wong, Geoff; Oxford University, Nuffield Department of Primary Care Health Sciences<br>Tinelli, M ; London School of Economics and Political Science, PSSRU<br>Frost, Faith; University of Worcester, Association for Dementia Studies<br>Bray, Jennifer; University of Worcester, Association for Dementia Studies<br>Hullah, Nigel; 3 Nations Working Group for Dementia
**Primary Subject Heading**:	Health services research
Secondary Subject Heading:	Geriatric medicine, Neurology, Mental health, Qualitative research, Evidence based practice
Keywords:	Dementia < NEUROLOGY, Organisation of health services < HEALTH SERVICES ADMINISTRATION & MANAGEMENT, SOCIAL MEDICINE, Old age psychiatry < PSYCHIATRY, Organisational development < HEALTH SERVICES ADMINISTRATION & MANAGEMENT, QUALITATIVE RESEARCH

SCHOLARONE™
Manuscripts

The sustainability of locally-driven centres for those affected by dementia: A protocol for the Get Real with Meeting Centres realist evaluation

Authors:

Thomas Morton, Association for Dementia Studies, University of Worcester, Worcester, UK.

Shirley B. Evans, Association for Dementia Studies, University of Worcester, Worcester, UK.

Dawn Brooker, Association for Dementia Studies, University of Worcester, Worcester, UK.

Tracey Williamson, Association for Dementia Studies, University of Worcester, Worcester, UK.

Geoff Wong, Nuffield Department of Primary Care Health Sciences, University of Oxford, Oxford, UK.

Michela Tinelli, Care and Policy Evaluation Centre, London School of Economics and Political Science, London, UK.

Faith Frost, Association for Dementia Studies, University of Worcester, Worcester, UK.

Jennifer Bray, Association for Dementia Studies, University of Worcester, Worcester, UK.

Nigel Hullah, 3 Nations Working Group for Dementia, Swansea, UK

Corresponding Author:

Dr Shirley B. Evans, Association for Dementia Studies, School of Allied Health and Community,

University of Worcester, Henwick Grove, Worcester WR2 6AJ.

Email: *shirley.evans@worc.ac.uk*. Tel: 01905 542664

For peer review only

The sustainability of locally-driven centres for those affected by dementia: A protocol for the Get Real with Meeting Centres realist evaluation

Thomas Morton,[1] Shirley B. Evans,[2] Dawn Brooker,[3] Tracey Williamson,[4] Geoff Wong,[5] Michela Tinelli,[6] Faith Frost,[7] Jennifer Bray,[8] Nigel Hullah[9]

ABSTRACT

Introduction: Improving support for people with early to moderate dementia to live at home in their communities is a global public health goal. Community adult social care is not robust in many parts of the UK, however, with the pandemic increasing pressure on services for this population. Community-led interventions can play a key role in supporting people post-diagnosis, helping delay decline, but many interventions struggle to sustain beyond 1-2 years. Meeting Centres (MCs) are one such intervention, which many UK community groups find attractive and achievable. However, it is not understood how these communities can ensure they are putting in place strategies that will help them sustain in the longer term, beyond start-up phase.

Methods and analysis: This Realist Evaluation aims to understand the factors affecting sustainability of MCs in rural areas and learn lessons from MCs that have sustained beyond three years. Data will be collected using mixed methods: interviews and group discussions with stakeholders involved at every level in three case study locations in England and Wales, analysed with Soft Systems modelling; a Discrete Choice Experiment exploring what people across the UK value and are willing to pay for MCs, analysed with regression modelling. All data will be synthesised using a Realist logic of analysis to build a theoretical model of how, why, for whom, in what contexts and to what extent MCs can be successfully implemented for the long-term.

Ethics and dissemination: As participants may lack capacity for informed consent, favourable ethical opinion was received from a Health Research Authority research ethics committee. Resulting recommendations will be of interest to stakeholders including those commissioning, planning,

For peer review only

running, supporting or attending MCs, as well as policymakers and healthcare professionals. Knowledge will be shared with emerging MCs to help accelerate scale up of this intervention.

Key words: *Dementia, post-diagnostic support, psychosocial intervention, integrated care, organisation of health services, community medicine, social medicine, old age psychiatry*

Strengths and limitations of this study

- A Realist approach is well suited to accommodate and account for the complexity of such 'real life' intervention programmes, as implemented under different conditions in different settings, to extract transferable conclusions.
- This study is designed to draw upon and disseminate a wide range of knowledge and expertise from people with extensive first-hand experience in tackling the issues involved in keeping a community-led dementia intervention running long-term.
- The Meeting Centre is an intervention type that is rapidly growing in popularity across the UK at this current time, hence this research well placed to offer timely practical insights to help multiple new community-led interventions of this type become established and avoid common pitfalls that may threaten sustainability.
- This study is designed to gather evidence for how to successfully sustain a Meeting Centre for people affected by dementia or similar community-led intervention in a rural UK context, not data on the effectiveness or otherwise of this particular intervention type.
- This research cannot guarantee solutions to all of the challenges to the sustainability of a community-led intervention such as a Meeting Centre; instead it may highlight some challenges that are insurmountable given current approaches and policy, regarding health and social care systems.

For peer review only

INTRODUCTION

Supporting people living with dementia (and those that, in turn, care for or otherwise support them) to live as well as possible in their communities, with timely psychosocial support, is a global public health goal[1]. However, support following a diagnosis of dementia is patchy across the UK[2], with people and families in some areas lacking any formal proactive support beyond occasional contact with primary care and third sector. With an aging population[3] and increasing pressure on already stretched health services[4] policy has for some time pointed to the need to move towards a model of social care where more people are cared for and supported at home, in the community. Improving provision of early, post-diagnosis support, improving support for family/informal carers and improving support for integrated care (involving the voluntary and independent sectors) – all in a more dementia-friendly community environment – are contemporary UK Government priorities for dementia care[2].

However, multiple pre-pandemic reports described a climate where the state of social care provision – mainly delivered piecemeal by private and third-sector organisations – is "precarious and dysfunctional" in many parts of the country[4] and in some areas has "broken down" creating "care deserts"[5]. There is an associated reliance on informal carers (e.g. family members) to step in to meet the needs of loved ones, but there is a growing recognition that informal carers' own health and wellbeing is often negatively impacted by their caring activities[6]. The detrimental health impact of social isolation and loneliness is also increasingly being recognised[7-8], with survey data revealing 60% of people living with dementia report loneliness, isolation and losing touch with people in their lives since diagnosis, around a quarter feeling they are not part of their community and that people avoid them[9]. Family carers can also be subject to such loneliness and isolation[10].

For peer review only

There have been various attempts to mitigate these challenges in communities across the UK, in the form of groups and activities for people with dementia and family/informal carers. These aim to serve a number of functions. However, there are significant gaps in social care for people affected by dementia across the UK[5-6,9]. Care systems are unprepared for the forecasted doubling of the number of people living with dementia (1.6 million) and tripling of social care costs by 2040[11]. Scaling up provision of evidence-based community initiatives for people with dementia and those that support them is imperative[12-18]. The benefits of community-based initiatives are now being recognised[14-18]. However, they face a variety of challenges in sustaining long-term. These challenges and how to meet them are much talked about in the dementia care policy, rhetoric and practice arenas but have received very little research attention, as identified in the SCI-Dem review [2018-20][19-20]. This research also showed many community initiatives are not sustained beyond 1-2 years.

Meeting Centres

Meeting Centres (MCs) for people affected by dementia are a community initiative based upon a successful Dutch model[12-13,21] that have emerged in the UK since 2015. MCs are distinct from day care, supporting both people with dementia and those that support them (e.g. children, partners and friends) together, and connecting people to each other and their community. They build on Dementia Friendly Communities and are a step up in support from Dementia Cafes. At their heart is a small social club (15 people per day plus supporting family, friends and carers), based in an ordinary community building, close-by to where people live. They typically operate up to three times per week, providing people the chance to build friendships, peer support, understand their problems, get help and prepare for the future. Evidence-based post-diagnostic interventions are also provided in MCs, geared to the needs of members and facilitated by a small team of staff and volunteers trained in person-centred dementia care and the Adaptation-Coping Model[22]. Following substantial European research [MeetingDem: 2014–17][14,23-25], MCs were recommended as a social care intervention for those affected by dementia[14] and found to help people, their families and

communities build resilience for the longer term[12-15,26-32]. The first MC in the UK opened in 2015, and there are now more than 30 either running or with funding to open in 2022, with accelerated interested from communities around the UK since lockdown restrictions were eased in 2021[33].

The focus is now turning from how to establish these interventions, to how to keep them going long-term, in the face of a challenging social care-funding climate. To date, early adopter MC sites have devised different strategies to mitigate against threats and circumstances affecting their successful continuation. A better understanding is needed of the issues MC stakeholders have faced and are likely to face, and what can be learnt from this to prevent 'reinvention of the wheel' and help ensure sustainability. This is particularly true in rural communities where people and families living with dementia face increased barriers to being able to access support, guidance and connection[34]. No MC-focused studies have yet investigated the factors that are key for the sustaining MCs after the start-up stage, and there is little focussing on this aspect with regards to other community-based interventions broadly serving a similar function and demographic. If these kinds of interventions cannot survive long term, the gap in provision will remain and the situation can be expected to worsen significantly with the rise in numbers of people living with dementia needing support.

Research question and overall aim: The understand the factors affecting the sustainability of Meeting Centres for people affected by dementia in rural areas, how these can best be tackled, and what lessons can be gleaned for emerging Meeting Centres.

Objectives

1: To empirically test the theory developed by the SCI-Dem review regarding the factors involved in sustainability of a community intervention for people affected by dementia.

2: To produce an in-depth transferable understanding of the key factors that may threaten the long-term delivery of an MC in the form of a refined Realist programme theory.

For peer review only

3: To explore people's willingness to pay for MC provision via a Discrete Choice Experiment (DCE) with those who support people living with dementia, triangulated with the perspectives of people living with dementia.

4: From the programme theory and DCE above, to build a model of how best to design, implement and deliver an MC under different conditions so it has the best chance of sustaining long-term.

5: To develop evidence-informed guidance materials for those in practice and evidence-informed recommendations for use at commissioning and policy level.

METHODS AND ANALYSIS

Project overview

This Realist Evaluation[35] will comprise primary data collection from three MC research sites, that have each taken different approaches to serving different rural community settings, to investigate what works, under what circumstances, for whom, how and why, regarding configuring MCs for long-term sustainability. Data collection will comprise interviews and focus group discussions with participants in a range of roles relating to the MC under investigation, along with documentary and demographic data from each site. We will produce a case study model of each MC site with the aid of Soft Systems Methodology (SSM)[36]. These models will be synthesised and analysed using a Realist logic of analysis[35] to generate theoretical causal chains of how contexts (background circumstances) can trigger mechanisms (responses and processes within people and organisations) to produce desired or undesired outcomes. We will also conduct a DCE survey regarding what activities and elements of MCs members most value and how much they are willing to pay for these, which will also feed into the overall Realist analysis. This work will be split across five work packages. **Figure 1** shows an overview of the study framework.

[Figure 1 here]

For peer review only

The use of Systems approaches to complement Realist thinking is an area of growing interest with multiple successful examples integrating the two in recent years[37-38]. Both Realist and Systems approaches are appropriate for this research as they aim primarily to deal with, and understand, the complexity of systems with human actors in real-world settings. Social care interventions such as MCs tend to be especially complex as they can involve multiple agencies and are embedded in a wider community setting, often with informal and impermanent elements making up part of how they work. Realist approaches focus on explaining the causal mechanisms of action that underlie complex programmes or interventions, to explain why they may be successful in some instances but not in others[39]. Soft Systems Methodology[36] (SSM) describes a process of enquiry to uncover real-world complexity by consulting those involved with a programme, to build up a conceptual model and determine what action can be taken to change things. The approach is designed to tackle organisational problems where the exact nature of the problem may not be agreed upon and need investigating. Hence, the issue of the sustainability of an MC programme, with all of the factors that could involve in a complex community-based setting, is a good fit for a combination of Realist and Systems approaches.

Patient and Public Involvement

Patient and participant involvement (PPI) will be channelled through each MC research site, led by dedicated members of the project team including a lay co-applicant who is living with dementia. Prior to the start of this study, PPI was carried out with members of one MC and lay members of the UK Meeting Centre Support Programme (UK-MCSP) National Reference Group, which includes members of DEEP (Dementia Engagement and Empowerment Project network)[40] and TIDE (Together In Dementia Everyday carers' network)[41]. Views were sought about the importance of the study, its design, focus, recruitment approaches, factors likely to affect participation, payment/other reward for PPI, dissemination, approaches to PPI in the study, support needs (e.g. PPI training/development) and interest in further involvement if the study was funded. PPI representatives will be fully

For peer review only

supported to input into data collection processes and materials, synthesis and interpretation of data, and the creation of recommendations and resources for dissemination, as well as channels of dissemination. We will also continue to work with the UK-MCSP National Reference Group, to provide steering input.

Work package 1 - Stakeholder group engagement and enquiry

Iterative stakeholder consultation throughout a project is a standard part of Realist Evaluation[42]. Stakeholders will include patient and public representatives (whose involvement is covered in the section above) as well as stakeholder professionals with experience of community dementia support. The latter will also be consulted regarding data collection processes and materials, synthesis and interpretation of data, creation of recommendations and resources for dissemination, and channels of dissemination. This work is organised as work package 1 as it is central to the progress of the evaluation.

Work package 2 – Data gathering and Meeting Centre case studies

We have identified three MCs in different rural communities that have been operational for over three years and that meet the Essential Features of Meeting Centres[43]: One in a small market town in Herefordshire (opened February 2016; rural; deprivation rank[44] around MC: 3,288 of 32,844 neighbourhoods in England); one in a larger market town in Worcestershire (opened September 2015; semi-rural; deprivation rank[44]: 17,429 of 32,844 neighbourhoods in England); One rural county in Mid Wales with four federated small town MCs run by the same organisation (opened March 2017; rural; deprivation rank[45]: areas ranging from 284 to 1,687 of 1,909 neighbourhoods in Wales). Study sites have been selected purposefully, as they have been able to continue operating for at least two years prior to the commencement of this study, notwithstanding some necessary pausing or alteration of activities during COVID-19 restrictions. In addition to different geographic and demographic factors, MCs at each site have taken their own individual approach to the implementation and delivery of the service.

For peer review only

Participants will likewise be selected purposefully for their role and involvement in each MC, and for the experience they might bring regarding issues outlined in the research question and objectives. The project team will work with MC leads to identify appropriate potential participants in each role and approach those in roles outside the MC itself. MC staff will approach MC attendees to invite them to participate in the study, help them to better understand the participant information and consent process to make an informed decision on taking part.

Interviews and focus groups will take place with those involved at every level, in the following manner per MC, as shown in **Table 1**:

**Role of participant in MC**	**Method of data collection**	**Number of participants**
MC attendees (people living with dementia)	Focus group and/or one to one interview (individual or supported by a partner)	6
MC attendees (people supporting someone with dementia)	Focus group and/or one to one interview	6
MC staff and volunteers	Focus group and/or one to one interview	6
Those involved with governance at each MC	One to one interview	4
Health/social care/third sector professionals involved in the local dementia care pathway	One to one interview	4

For peer review only

Other stakeholders involved in local dementia strategy e.g. Dementia Friendly Communities Programme	One to one interview	4
		**Total:30**

Table 1. Participants and methods of data collection

We anticipate 30 participants per MC. This number should give us a range of perspectives per type of participant, at different levels, to draw on to create SSM conceptual models, while also being realistic in terms of numbers available to take part at each MC and practically manageable within the scope and timeframe of the study. Interviews and discussions are anticipated to take between 20 minutes and one hour and will be conducted on site at each MC where possible, though we anticipate a proportion will be conducted via virtual video conferencing or telephone due to the impact of the COVID -19 or participant preference.

Interview schedules were developed and piloted with stakeholder input and can be found in **Supplementary file 1**. The development of these were guided in part by the factors involved in the sustainability of community-based interventions found in the SCI-Dem Realist Review[19-20] (summarised in **Supplementary file 2**) but also a modified SSM "BATWOE" structure as outline in **Table 2.**

**B**	Beneficiaries (who is the system aimed at helping, e.g. people living with dementia and those that support them)
**A**	Actors (people's roles and functions in the system, e.g. staff, volunteers, governors, referrers, social care professionals, community stakeholders)

For peer review only

**T**	Transformations (i.e. going from start-up MC to established MC to stable and thriving MC)
**W**	Worldview (e.g. how do things work regarding sustainability, what are the challenges and what should be done?)
**O**	Ownerships (i.e. who or what can influence or thwart success of an MC)
**E**	Environment (i.e. what are the background contextual factors that could boosts or constrain success?)

Table 2. The elements of the SSM "BATWOE" structure[36-37].

The content of interview and discussion transcripts pertaining to the sustainability of MCs will be extracted and categorised by theme (generated both deductively and inductively) using NVivo qualitative data analysis software[46]. Two researchers will undertake this and independently theme and categorise 10% of each other's workload and compare and discuss any discrepancies for standardisation purposes. Any remaining disagreements will be discussed with the whole research team, as will the final list of data categories. Data will then be analysed using SSM procedures[36], applying a sequence of steps to the data from each MC site to build a conceptual model of how things work which will be returned to participants to review. This will act as the basis for developing a Realist programme theory with all data combined (see work package 4)

Work package 3 - A DCE to measure people's willingness to pay for successful MCs

Successful implementation of evidence-based services in health and social care depends largely on the fit of the services with the values and priorities of stakeholders who are shaping and participating in their delivery and use[47]. A flexible health economics tool for measuring choices in health and social care-related settings is the DCE[48], which measures preferences from individual decision-makers over alternative scenarios (or service provisions). Each alternative is described by

For peer review only

several attributes (or characteristics) and the choices made between two or more competing scenarios subsequently determine how preferences are influenced by each attribute (e.g., which attributes are valued as well as their relative importance). It can also provide a measure of the overall value attached to different alternatives (and identify optimal service provision that meets stakeholder requirements and have the best chance of sustainability in the long term). When a cost attribute is included, the DCE technique can also allow weighing of the benefits and costs of service provisions and calculating: how much stakeholders may be willing to pay for a particular service provision and measure how their willingness to pay may vary from current provision to their preferred option. Hence a DCE survey will be developed to measure people's preferences for what can MC provides in terms of types of activities, social opportunities and emotional support, as well as the frequency of meetings, their availability and costs.

The DCE follows steps as laid out in the standard guidelines[49]. The attributes (characteristics) and their various levels will be informed by the Essential Features of a Meeting Centre[43] and the Adaptation-Coping Model[22], as well as data from our three case study sites regarding the typical range of cost to members and days/time open or available per week. Qualitative data regarding what members value about MCs, from early Work Package 2 interviews, will be then used to validate and refine the attributes and levels. PPI stakeholders will be also consulted on the development of the presentation, wording and format of the survey. Due to the cognitive load involved in completing the survey, our target group will be people who support/care for an attendee who is living with dementia, reporting preferences on their behalf. Triangulation focus groups (n=3-6 per MC site) will be conducted with people living with dementia at each MC to ensure the views of this population are not excluded. The questionnaire will be distributed via staff at all UK MCs, as either an online survey or paper copy, depending on preference, with the aim of securing responses from more than 300 people[49-50]. Efficient experimental design techniques will be applied to create the DCE choice set[51] and data will be modelled using logit techniques (NLOGIT 6 software).

For peer review only

Work package 4 - Realist theory refinement and development of materials for practice

Data from all work packages, categorised and organised as themes, will be further analysed using the same Realist logic of analysis[35] as the SCI-Dem Realist Review[19-20], to develop a Realist programme theory. This second stage of analysis is needed to understand causation – i.e. how differing contexts in different MCs trigger different mechanisms (the hidden causal processes within people and organisations) to cause desired or undesired outcomes. This will test the programme theory produced during the SCI-Dem review, and act as a basis for developing recommendations and materials that explain how to best implement community-based interventions to sustain past the start-up phase, in a variety of settings. As it is developed, this programme theory will be presented back to stakeholders in each MC (see Work Package 1) for feedback and advice that will be used to further validate and refine it. We will use this understanding to develop (amongst other things) tips on best practice, what pitfalls to avoid and what challenges may need to be planned for, grounded in the experiences and models of working of those involved with the three MCs at every level. Materials to disseminate this learning to those in practice, and at a commission level, will be developed in collaboration with stakeholders.

Work package 5 – Investigation of who MCs do not reach or benefit

In December 2021 the NIHR approved an expression of interest to add a further work package to the Get Real study. This work package will investigate who is not being reached by MC support and why, in the following ways: A comparison of demographic data regarding who attends each case study MC and whether they are representative of the population of their communities (and if there are any demographic groups clearly under-represented); additional interviews with existing stakeholders involved in the MC referral process specifically regarding why referrals might not be made or declined; identification, recruitment and additional interviews with dyad pairs (n=10) who have been made are aware of an MC but have decided not to attend or stopped attending, to understand what happened and why. This will again feed into Work Package 4.

For peer review only

ETHICS AND DISSEMINATION

Favourable ethical opinion for the whole study was received from a Health Research Authority (HRA) Research Ethics Committee (REC) prior to starting recruitment and data collection. This was a requirement of the NIHR funders and in any case deemed necessary because some participants may lack the capacity to provide informed consent, or their ability to consent may change over time. Under the terms of the Mental Capacity Act 2005[52], people who lack the capacity to consent cannot be included in research unless the research concerns their condition. This research is concerned with improving the implementation and sustainability of social care interventions related to the condition of dementia, in order to improve the provision of support for the benefit of people living with dementia and those who support them. Favourable ethical opinion was given by Wales REC4 (21/WA/0185).

Consent and risk of distress

Participant information (see **Supplementary file 3**) and consent documents (see **Supplementary file 4**) were developed for a range of possible participants in line with HRA guidance[53]. It was felt important to include MC attendees in the research for two reasons: 1) to ensure the perspectives of the people MCs are designed for are fully and authentically represented in line with a "Nothing about us, without us" ethos; 2) to access key knowledge and experience, because attendees are in a position to offer key first-hand perspectives not directly available to others such as staff and governors, particularly regarding the factors that can encourage or act as a barrier to engaging with and attending a local MC. In order to undertake research ethically with these participants, the research team developed sensitive and relevant practices of informing and negotiating consent to participate. **Figure 2** outlines the process of determining ability or provide informed consent:

[Figure 2 here]

For peer review only

The focus of the interviews, group discussions and questionnaire questions will be the MC, people's experiences and opinions of it and preferences regarding what it provides. This focus is not expected to include topics that might be overly personal, sensitive, embarrassing or upsetting. Nevertheless, there is a risk semi-structured interviews and discussion may stray into personal or sensitive areas that participants may not be comfortable with. For this reason, question topics will be explained to participants (and their consultee if they have one) before and interview or group discussion. Participants will also be told they do not have to answer any question they are not comfortable with and that they can stop the interview or leave the discussion at any time. The researchers will also be on alert for any signs of distress and will pause proceedings if a participant shows signs of discomfort or upset, or of reluctance to take part, at any time before or during interview or group discussion. The participant (and their consultee if they have one) will be asked if they are happy to continue, would prefer to re-arrange for another time, or withdraw altogether. If there is any sign of discomfort with a sensitive or personal topic that is not necessary to discuss, researchers will automatically move the conversation on to a topic that is not personal or sensitive. Participants who are attendees of the MC will undertake interviews and discussions at the Meeting Centre itself, with trained staff on hand to help if they do become distressed or upset. Questions will also be framed in terms of how to overcome challenges for success, and what can be learned to help success in the future, and piloted to ensure there is no suggestion that our research means a MC's future is under threat or that blame for any perceived failings in running it is being sought, which could be upsetting for participants.

The DCE questionnaire will be anonymous and only asks for opinions and preferences, hence there are not anticipated to be any risks in taking part. Participant information explaining the nature and objectives of the questionnaire, and consent questions, will be presented as part of the questionnaire itself. We will also hold focus group discussions with participants living with dementia in our MC case study sites, on the same questions covered in the DCE questionnaire, to triangulate, with an identical consent process to the other interviews and group discussions.

For peer review only

Regarding COVID-19 risks, we will only undertake face-to-face data collection if local and national guidance allows visitors to MCs and the MC and potential participants feel safe to do so. This situation will be continually monitored. Where face-to-face data collection is not possible or agreed, we will move to online data collection supplemented by telephone calls where preferred.

Data management and confidentiality

All data gathered that may identify participants will be kept in password protected files and folders on the University of Worcester's secure cloud-based storage, with unique participant identification codes used in data storage, known only to the research team. This includes interview and discussion recordings and transcripts, consent documentation and any personal data collected to maintain contact with participants. Where physical copies of data are necessary, they will be kept in locked cabinets on University premises. Face-to-face meetings will be recorded digitally on an encrypted recording device, online meetings using facilities provided by that online platform, with recordings transferred at the first opportunity and deleted once transcribed by a member of the research team or a trusted external transcription service and checked for accuracy.

Transcripts will be anonymised through the removal of names and other personal information. However, it should be noted that for the sake of analysis some information may be necessary to retain on a participant's role within the system of each MC, or information specific to the local context, which may make jigsaw identification possible. Hence participants will be alerted to this when taking consent and their preferences on anonymity and identification will be gathered and checked before reporting. In reporting, MCs will not be identified specifically by name or town, but only by region and pertinent demographic factors.

Dissemination

The UK-MCSP[54], led by the University of Worcester, has created a UK-wide Community of Learning and Practice comprising 300 organisations involved or interested in providing MCs in their

For peer review only

communities, with more than 30 MCs now on-stream and more planned across the country. There is also a National Reference Group comprising 30 national organisations drawn from policy and practice. This networked community of stakeholders will be consulted to help co-create accessible resources and disseminate them according to their preferences, to ensure knowledge from this research is accessible to those involved in the day-to-day governance, management and running of MCs in the UK. This will also involve workshop activities with PPI representatives/stakeholders within the case study MCs. Dissemination will take place through these MC network channels, as well as to a wider audience through practitioner workshops, webinars, blogs, newsletters and social media. Learning form this study will also be incorporated into training for emerging and existing MC personnel provided by the University of Worcester.

In addition to academic publications and conference presentations, outputs will include an accessible publication and website downloads for a non-academic audience that will detail the three case studies and overall analysis, useful for all in similar community settings looking for a flexible template that they might implement; there will also be published evidence-based guidelines for commissioners and providers of community-based interventions for people affected by dementia. Specifically, Worcestershire County Council will use knowledge from this research to support new MCs in the county. A publicly accessible report summary will be available on University of Worcester Association for Dementia Studies website[55] post-study and findings will be promoted widely at MCs to reach study participants via posters, presentations and leaflets. A full study report will be made available on request.

Word count 4,350

Figure legends

Figure 1. Overview of Get Real with Meeting Centres project.

For peer review only

Figure 2. Consent process flow diagram.

Author affiliations and email

1 Association for Dementia Studies, University of Worcester, Worcester, UK. *t.morton@worc.ac.uk*

2 Association for Dementia Studies, University of Worcester, Worcester, UK. *shirley.evans@worc.ac.uk*

3 Association for Dementia Studies, University of Worcester, Worcester, UK. *d.brooker@worc.ac.uk*

4 Association for Dementia Studies, University of Worcester, Worcester, UK. *Tracey.Williamson@wales.nhs.uk*

5 Nuffield Department of Primary Care Health Sciences, University of Oxford, Oxford, UK. *geoffrey.wong@phc.ox.ac.uk*

6 Care and Policy Evaluation Centre, London School of Economics and Political Science, London, UK. *M.Tinelli@lse.ac.uk*

7 Association for Dementia Studies, University of Worcester, Worcester, UK. *f.frost@worc.ac.uk*

8 Association for Dementia Studies, University of Worcester, Worcester, UK. *j.bray@worc.ac.uk*

9 3 Nations Working Group for Dementia, Swansea, UK. *nigelhullah@aol.co.uk*

Corresponding Author Dr Shirley Evans, Association for Dementia Studies, School of Allied Health and Community, University of Worcester, Henwick Grove, Worcester WR2 6AJ. Email: *shirley.evans@worc.ac.uk*. Tel: 01905 542664

Twitter Follow Thomas Morton @ThomasMortonADS

Author Contributions DB, SBE and TM conceptualised the study with input from TW, GW, MT, FF, JB and NH. TM wrote the first draft of this manuscript. SBE, DB, TW, GW, MT, FF, JB and NH critically contributed to and refined this manuscript. All authors have read and approved the final manuscript.

For peer review only

Acknowledgements The authors would like to thank all those who shared their invaluable experience and contributed to advising and guiding this project as a stakeholder consultant, and all those involved with Meeting Centres, whether running, attending, supporting or in governance, who have agreed to take part in this research or facilitate others to do so.

Funding This paper presents independent research funded by the National Institute for Health Research (NIHR) under its Research for Patient Benefit (RfPB), Research for Social Care Programme (Grant Reference Number NIHR201861). The views expressed are those of the authors and not necessarily those of the NIHR or the Department of Health and Social Care.

Competing interests GW is Deputy Chair of the National Institute for Health Research Health Technology Assessment Prioritisation Committee: Integrated Community Health and Social Care (A).

Ethics approval This project has been reviewed by Health Research Authority research ethics committee Wales REC 4. REC reference: 21/WA/0185; IRAS project ID: 294636.

ISRCTN registration number 10332079

Open Access This is an Open Access article distributed in accordance with the terms of the Creative Commons Attribution (CC BY 4.0) license, which permits others to distribute, remix, adapt and build upon this work, for commercial use, provided the original work is properly cited. See: http:// creativecommons.org/licenses/by/4.0/

REFERENCES

1. World Health Organisation. *Global action plan on the public health response to dementia 2017-2025.* Geneva: World Health Organisation, 2017. https://www.who.int/publications-detail/global-action-plan-on-the-public-health-response-to-dementia-2017---2025

2. Department of Health. *Prime Minister's challenge on dementia 2020.* London: Department of Health, 2015. https://assets.publishing.service.gov.uk/government/uploads/system/uploads/attachment_data/file/414344/pm-dementia2020.pdf

3. Office for National Statistics (ONS). *Living longer: Caring in later life.* London: ONS, 2019. https://www.ons.gov.uk/peoplepopulationandcommunity/birthsdeathsandmarriages/ageing/articles/livinglongerhowourpopulationischangingandwhyitm atters/2019-03-15

4. Care Quality Commission. *The state of health care and adult social care in England 2018/19.* Newcastle-upon-Tyne: Care Quality Commission, 2019. https://www.cqc.org.uk/sites/default/files/20191015b_stateofcare1819_fullreport.pdf

5. Incisive Health. *Care deserts: the impact of a dysfunctional market in adult social care provision.* London: Incisive Health, 2019. https://www.ageuk.org.uk/globalassets/age-uk/documents/reports-and-publications/reports-and-briefings/care-- support/care-deserts---age-ukreport.pdf

6. Age UK. *Briefing: Health and Care of Older People in England 2019.* London: Age UK, 2019. https://www.ageuk.org.uk/globalassets/ageuk/documents/reports-and-publications/reports-and-briefings/health--wellbeing/age_uk_briefing_state_of_health_and_care_of_older_people_july2019.pdf

7. Cornwell EY, Waite LJ. Social disconnectedness, perceived isolation and health among older adults. *J Health Soc Behav* 2009;50:31–48.doi.org/10.1177/002214650905000103

For peer review only

8. Holt-Lunstad J, Smith TB, Baker M, *et al.* Loneliness and social isolation as risk factors for mortality: a meta-analytic review. *Perspect Psychol Sci* 2015;10:227–237.doi.org/10.1177/1745691614568352

9. Alzheimer's Society. *A lonely future: 120,000 people with dementia living alone, set to double in the next 20 years.* London: Alzheimer's Society, 2019. https://www.alzheimers.org.uk/news/2019-05-15/lonely-future-120000-people-dementia-living-alone-set-double-next-20-years

10. Brodaty H, Donkin M. Family carers of people with dementia. *Dialogues Clin Neurosci* 2009;11:217–228.pmid:http://www.ncbi.nlm.nih.gov/pubmed/19585957

11. Wittenberg R, Hu B, Barraza-Araiza L, *et al. Projections of older people with dementia and costs of dementia care in the United Kingdom, 2019-2040 (CPEC Working Paper 50).* London: LSE, 2019. https://www.alzheimers.org.uk/sites/default/files/2019-11/cpec_report_november_2019.pdf

12. Dröes RM, Breebaart E, Meiland FJ, *et al.* Effect of Meeting Centres Support Program on feelings of competence of family carers and delay of institutionalization of people with dementia. *Aging Ment Health* 2004;8:201-211. doi.org/10.1080/13607860410001669732

13. Dröes RM, Meiland FJM, Schmitz M, *et al.* Effect of combined support for people with dementia and carers versus regular day care on behaviour and mood of persons with dementia: results from a multicentre implementation study. *Int J Geriatr Psychiatry* 2004;19:1-12.

14. Brooker D, Evans SC, Evans SB, *et al.* Evaluation of the implementation of the Meeting Centres Support Program in Italy, Poland, and the UK; exploration of the effects on people with dementia. *Int J Geriatr Psychiatry* 2018;33:883-892. doi: 10.1002/gps.4865.

15. Evans SB, Evans SC, Brooker D, *et al.* The impact of the implementation of the Dutch combined Meeting Centres Support Programme for family caregivers of people with dementia in Italy, Poland and UK. *Aging Ment Health* 2020;24:280-290. doi: 10.1080/13607863.2018.1544207

For peer review only

16. Lord K, Beresford-Dent J, Rapaport P, *et al.* Developing the New Interventions for independence in Dementia Study (NIDUS) theoretical model for supporting people to live well with dementia at home for longer: a systematic review of theoretical models and Randomised Controlled Trial evidence. *Soc Psychiatry Psychiatr Epidemiol* 2020;55:1-14. doi: 10.1007/s00127-019-01784-w

17. McDermott O, Charlesworth G, Hogervorst E, *et al.* Psychosocial interventions for people with dementia: a synthesis of systematic reviews. *Aging Ment Health* 2019;23:393–403. doi: 10.1080/13607863.2017.1423031

18. Van't Leven N, Prick A-EJC, Groenewoud JG, *et al.* Dyadic interventions for community-dwelling people with dementia and their family caregivers: a systematic review. *Int Psychogeriatr* 2013;25:1581-1603. doi:10.1017/S1041610213000860. pmid:http://www.ncbi.nlm.nih.gov/pubmed/23883489

19. Morton T, Atkinson T, Brooker D, *et al.* Sustainability of community-based interventions for people affected by dementia: a protocol for the SCI-Dem realist review. *BMJ Open* 2019;9:e032109. doi: 10.1136/bmjopen-2019-032109

20. Morton T, Wong G, Atkinson T, *et al.* Sustaining community-based interventions for people affected by dementia long term: the SCI-Dem realist review. *BMJ Open* 2021;11:e047789. doi: 10.1136/bmjopen-2020-047789

21. Dröes RM, Meiland FJM, de Lange J, *et al.* The meeting centres support programme; an effective way of supporting people with dementia who live at home and their carers. *Dementia-London* 2003;2:421-438.

22. Brooker D, Evans S, Droes RM. Framing outcomes of post-diagnostic psychosocial interventions in dementia: The Adaptation-Coping Model and Adjusting to Change. *Working with Older People* 2017;21(1). doi: 10.1108/WWOP-12-2016-0039

For peer review only - http://bmjopen.bmj.com/site/about/guidelines.xhtml

23. Dröes RM, Meiland FJM, Evans S, *et al.* Comparison of the adaptive implementation and evaluation of the Meeting Centers Support Program for people with dementia and their family carers in Europe; study protocol of the MEETINGDEM project. *BMC Geriatr* 2017;17:79.

24. Mangiaracina F, Chattat R, Farina E, *et al.* Not re-inventing the wheel: the adaptive implementation of the meeting centres support programme in four European countries. *Aging Ment Health* 2016;21:40–48. doi:10.1080/13607863.2016.1258540

25. The MeetingDem Network. *The MeetingDem Network website.* Amsterdam: VU University Medical Centre, 2018. https://www.meetingdem.eu/

26. Dröes RM, Meiland FJM, Schmitz MJ, *et al.* Effect of the Meeting Centres Support Program on informal carers of people with dementia: Results from a multi-centre study. *Aging Ment Health* 2006;10:112-24. doi: 10.1080/13607860500310682

27. Dröes RM, Meiland FJM, Schmitz MJ, *et al.* How do people with dementia and their carers evaluate the Meeting Centres Support Programme? *Non-Pharmacological Therapies in Dementia* 2011;2(1).

28. Lion KM, Szcześniak D, Bulińska K, *et al.* Does the Meeting Centre Support Programme decrease the experience of stigmatisation among people with cognitive deficits? *Aging Ment Health* 2021;25:160-169. doi:10.1080/13607863.2019.1683815

29. Mazurek J, Szcześniak D, Lion KM, *et al.* Does the Meeting Centres Support Programme reduce unmet care needs of community-dwelling older people with dementia? A controlled, 6-month follow-up Polish study. *Clin Interv Aging* 2019;14:113-122. https://doi.org/10.2147/CIA.S185683

30. Meiland FJM, Dröes RM, de Lange J, *et al.* (2010). Predictors of effective support in meeting centers for people with dementia and their carers. *Non-Pharmacological Therapies in Dementia* 2010;1:251-270.

For peer review only

31. Szcześniak D, Dröes R, Meiland F, *et al.* Does the community-based combined Meeting Center Support Programme (MCSP) make the pathway to day-care activities easier for people living with dementia? A comparison before and after implementation of MCSP in three European countries. *Int Psychogeriatr* 2018;30:1717-1734. doi:10.1017/S1041610217002885

32. Szcześniak D, Rymaszewska J, Saibene FL, *et al.* Meeting centres support programme highly appreciated by people with dementia and carers: a European cross-country evaluation. *Aging Ment Health* 2021;25:149-159. doi:10.1080/13607863.2019.1683814

33. Association for Dementia Studies. *ADS blog, November 18, 2021: Meeting Centres – what's the situation?* Worcester: University of Worcester, 2021. https://adsdementiablog.wordpress.com/2021/11/18/meeting-centres-whats-the-situation/#more-2075

34. Bould E, Ball A, Donoghue T, *et al. Dementia-Friendly Rural Communities Guide.* London: The Alzheimer's Society, 2018.

35. Pawson R, Tilley N. *Realistic Evaluation.* London: Sage Publications, 1997.

36. Checkland P. *Systems Thinking, Systems Practice.* New York: J. Wiley, 1999.

37. Dalkin S, Lhussier M, Williams L, *et al.* Exploring the use of Soft Systems Methodology with realist approaches: A novel way to map programme complexity and develop and refine programme theory. *Evaluation* 2018;24:84–97. doi.org/10.1177/1356389017749036

38. Williams L, Rycroft-Malone J, Burton CR, *et al.* Improving skills and care standards in the support workforce for older people: a realist synthesis of workforce development interventions. *BMJ Open* 2016;6:e011964. doi:10.1136/bmjopen-2016-011964

39. Hewitt G, Sims S, Harris R. The realist approach to evaluation research: An introduction. *International Journal of Therapy and Rehabilitation* 2012;19:250–9.

For peer review only

40. DEEP. DEEP: The UK Network of Dementia Voices website, 2021. Available: http://dementiavoices.org.uk

41. TIDE. TIDE: Together in Dementia Everyday website, 2021. Available: https://www.tide.uk.net/

42. Wong G, Westhorp G, Greenhalgh J, *et al.* Quality and reporting standards, resources, training materials and information for realist evaluation: the RAMESES II project. *Health Services and Delivery Research* 2017;5(28). https://doi.org/10.3310/hsdr05280

43. Brooker D. *The Essential Features of a Meeting Centre: UK Meeting Centres Support Programme (UKMCSP).* Worcester: University of Worcester, 2019. https://www.worcester.ac.uk/documents/Essential-Features-of-a-Meeting-Centre-booklet-November-2019-edition.pdf

44. Ministry of Housing, Communities & Local Government. *English Indices of Deprivation 2019.* London: Ministry of Housing, Communities & Local Governmen, 2019. Available: http://imd-bypostcode.opendatacommunities.org/imd/2019

45. Welsh Government. *Welsh Index of Multiple Deprivation 2019.* Cardiff: Welsh Government, 2019. Available: https://gov.wales/welsh-index-multiple-deprivation-full-indexupdate-ranks-2019

46. QSR International. *NVivo (Version 12).* Melbourne: QSR International Pty Ltd, 2018. https://www.qsrinternational.com/nvivo-qualitative-data-analysis-software/home

47. Salloum RG, Shenkman EA, Louviere JJ, *et al.* Application of discrete choice experiments to enhance stakeholder engagement as a strategy for advancing implementation: a systematic review. *Implement Sci* 2017;12:140. https://doi.org/10.1186/s13012-017-0675-8

48. de Bekker-Grob EW, Ryan M, Gerard, K. Discrete choice experiments in health economics: a review of the literature. *Health Econ* 2021;21:145-172. doi: 10.1002/hec.1697

For peer review only

49. Johnson FR, Lancsar E, Marshall D, *et al.* Constructing experimental designs for discrete-choice experiments: report of the ISPOR Conjoint Analysis Experimental Design Good Research Practices Task Force. *Value Health* 2013;16:3–13. doi:10.1016/j.jval.2012.08.2223

50. de Bekker-Grob EW, Donkers B, Jonker MF, *et al.* Sample size requirements for discrete-choice experiments in healthcare: a Practical Guide. *Patient* 2015;8:373–384. https://doi.org/10.1007/s40271-015-0118-z

51. Choice Metrics. *Ngene: Next generation stated choice experiments.* Sydney: Choice Metrics, 2018. http://www.choice-metrics.com

52. United Kingdom. *Mental Capacity Act 2005, c.9.* Available: https://www.legislation.gov.uk/ukpga/2005/9/contents

53. NHS Health Research Authority. *Consent and participant information guidance.* London: NHS Health Research Authority, 2021. Available: http://www.hra-decisiontools.org.uk/consent/

54. The Association for Dementia Studies. *UK Meeting Centres Support Programme (UK MCSP) webpage.* Worcester: University of Worcester, 2018. Available: https://www.worcester.ac.uk/about/academic-schools/school-of-allied-health-and-community/allied-health-research/association-for-dementiastudies/ads-research/uk-meeting-centres.aspx

55. Association for Dementia Studies. *Dementia research webpage.* Worcester: University of Worcester, 2022. Available: https://www.worcester.ac.uk/about/academic-schools/school-of-allied-health-and-community/allied-health-research/association-for-dementia-studies/ads-research/

For peer review only

Get Real with Meeting Centres: Overview Diagram

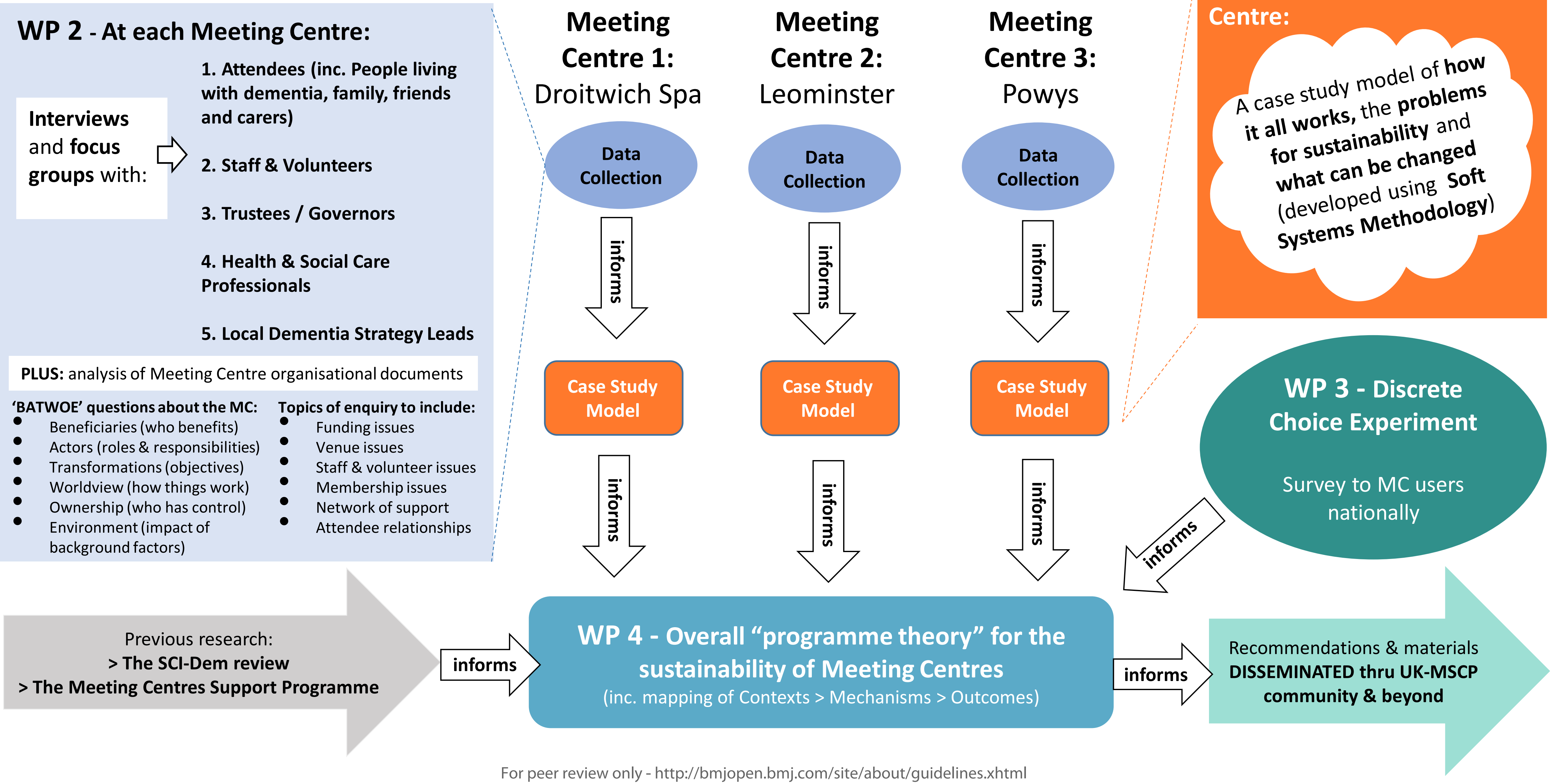

Note: Stakeholders (including patient and public representatives) at each MC - and within the UK-MCSP - will be consulted for input to each stage (WP 1)

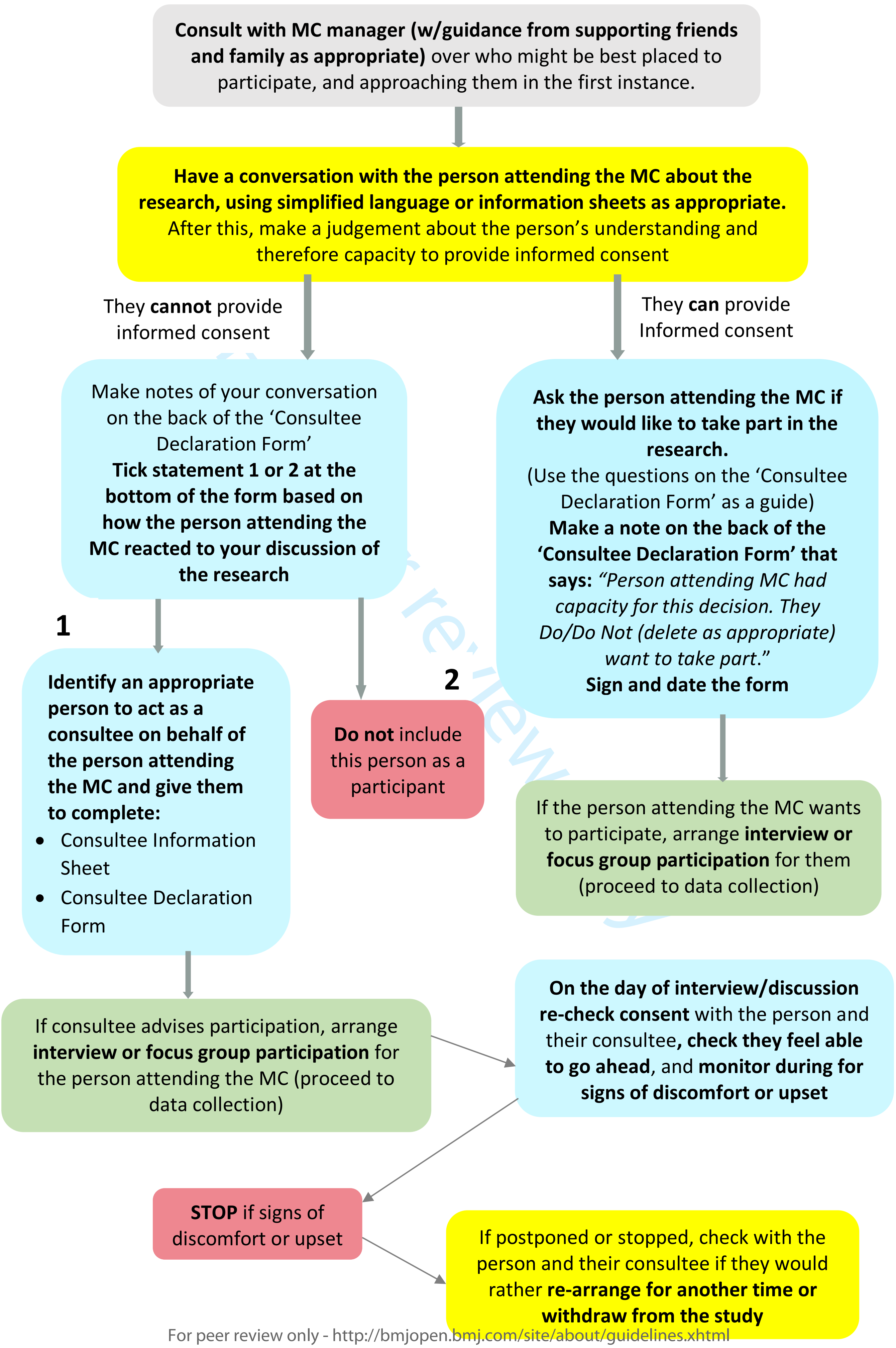
Consult with MC manager (w/guidance from supporting friends and family as appropriate) over who might be best placed to participate, and approaching them in the first instance.
Have a conversation with the person attending the MC about the research, using simplified language or information sheets as appropriate. After this, make a judgement about the person's understanding and therefore capacity to provide informed consent
They **cannot** provide informed consent
They **can** provide Informed consent
Make notes of your conversation on the back of the 'Consultee Declaration Form' **Tick statement 1 or 2 at the bottom of the form based on how the person attending the MC reacted to your discussion of the research**
Ask the person attending the MC if they would like to take part in the research. (Use the questions on the 'Consultee Declaration Form' as a guide) **Make a note on the back of the 'Consultee Declaration Form' that says:** *"Person attending MC had capacity for this decision. They Do/Do Not (delete as appropriate) want to take part."* **Sign and date the form**
1
Identify an appropriate person to act as a consultee on behalf of the person attending the MC and give them to complete:
- Consultee Information Sheet
- Consultee Declaration Form
2
Do not include this person as a participant
If the person attending the MC wants to participate, arrange **interview or focus group participation** for them (proceed to data collection)
If consultee advises participation, arrange **interview or focus group participation** for the person attending the MC (proceed to data collection)
On the day of interview/discussion re-check consent with the person and their consultee**, check they feel able to go ahead**, and **monitor during for signs of discomfort or upset**
STOP if signs of discomfort or upset
If postponed or stopped, check with the person and their consultee if they would rather **re-arrange for another time or withdraw from the study**

Interview/Group Discussion Schedule

For Meeting Centre personnel

Note: Which questions will be asked will be tailored to whether the interviewee has a strategic role or an operational role, in order to keep the number of questions concise and relevant, appropriate and understandable.

Introductions

Introduction of research staff and explanation of purpose:

"The purpose of this interview is to better understand how Meeting Centres have got up and running and how they have kept going over the past few years. We want to ask people for their knowledge and experience of how their Meeting Centre is run and what has made it a success, so that we can learn from it.

We will use what we learn to develop user-friendly guidance and materials to best help Meeting Centres survive and thrive in the long-term. We will also use this information to help policy makers and government understand what they can do to improve this situation. Our project will support the growth of more Meeting Centres to support people in desperate need. Thank you for agreeing to assist with this."

- Check provision of relevant study information sheet
- Confirm informed consent

Clarification of role:

- Ask for a description of the participant's role
- What do you do at the Meeting Centre? What are you responsible for?
- How did you get involved with the Meeting Centre?
- How long have you been involved with the Meeting Centre and has your role changed over time?

For peer review only - http://bmjopen.bmj.com/site/about/guidelines.xhtml

Get Real with Meeting Centres

1: Questions about who the Meeting Centre is aimed at helping:

- Who can become a member and who else benefits?
- How do you assess if a Meeting Centre is appropriate for someone?
- How do you find people? Do they come to you or do you approach them?
- What PR is done?
- What makes people want to come along and keeps people coming back?
- What can put people off coming or lead people to leave?
- Whose needs might not be met and why?
- To what extent do people supporting other attendees (e.g. friends/family) attend themselves?
- Do friends and family feel they also benefit? Are there challenges for them (e.g. arranging transport, fit with working hours)?
- Are all attendees happy with the programme of activities? What is most enjoyed and what could be better?
- How often do you reflect/consult on what is being provided and how well it meets the changing needs of attendees over time?

2: Questions about people's roles at the Meeting Centre:

- Who is responsible for what regarding the planning and running for the Meeting Centre?
- How many people are there in each of the different roles and is that the preferred number?
- What is their background and how did they come to be involved/how were they recruited?
- Have any left those roles any why?
- What training have personnel had and how did that happen?
- Are attendees involved in decision making?
- Are there any external people (e.g. referrers, community stakeholders) who play a key role in supporting the Meeting Centre? How did they come to be involved?
- Are there any external people (e.g. care professionals, practitioners, people who deliver a service) who come into the Meeting Centre?
- Are there any challenges or points of tension in working with external people?

3: Questions about how a Meeting Centre has developed:

- What were the most important things to have in place when setting up? What helped or hindered these things?
- What are the most important things to have in place now, and going forward? Are these the same things, or is the focus different now?
- How has the Meeting Centre and its work changed over time?
- What and links to the broader community have been developed, and how?
- How did the Meeting Centre respond to the COVID-19 pandemic? How has that affected how it might function in the future?

IRAS ID: 294636 Version: v0.3 09/06/21

4: Questions about how well things work.

- What things do you think are most key to keeping a Meeting Centre going long term?
- What are the key challenges in keeping going long term?
- What is good or not so good about how the Meeting Centre is run and its current situation?
- What opportunities are there for things to change?

5: Questions about who has the power to influence the success of a Meeting Centre, including:

- Whose support (person or organization) is essential to making a success of the Meeting Centre?
- What could influence their attitudes and decisions regarding the Meeting Centre?
- How could local or national authorities help or hinder the success of the Meeting Centre?
- Who in the Meeting Centre team could the Meeting Centre not continue without?
- How does the Meeting Centre meet its costs? What are the various sources of funding?
- How sustainable are these sources of funding? How difficult are they to come by?

6: Questions about the place and background circumstances around the Meeting Centre, including:

- Is the venue appropriate and working well for attendees and personnel?
- How does the local healthcare pathway fit with the Meeting Centre? Does it help, hinder or have no bearing on the Meeting Centre's success?
- How does the local/wider community view the Meeting Centre? Are there any misconceptions? Does it help, hinder or have no bearing on the Meeting Centre's success?
- How does the geography of the area (e.g. rural or urban) affect the Meeting Centre's success?
- How do people get to the Meeting Centre? What distance do they come from?
- How suitable is public transport? How available is community transport?

Interview closure

Is there anything else you'd like to tell us that you feel we should have asked you?

Many thanks for your time.

(Confirm any arrangements for checking accuracy of interpretation of views).

For peer review only - http://bmjopen.bmj.com/site/about/guidelines.xhtml

Interview/Group Discussion Schedule

For people who attend Meeting Centres

Note: Which questions are asked will be tailored to whether the interviewee is living with dementia or supporting someone who is, or if people are being interviewed as a pair, to ensure the questions are appropriate, understandable and kept concise and relevant. Concrete examples of people and organizations known to the participant will be used where possible, to make the questions less abstract.

For peer review only

Introductions

Introduction of research staff and explanation of purpose:

"The purpose of this interview is to better understand how Meeting Centres have got up and running and how they have kept going over the past few years. We want to ask people for their knowledge and experience of how their Meeting Centre is run and what has made it a success, so that we can learn from it.

We will use what we learn to develop user-friendly guidance and materials to best help Meeting Centres survive and thrive in the long-term. We will also use this information to help policy makers and government understand what they can do to improve this situation. Our project will support the growth of more Meeting Centres to support people in desperate need. Thank you for agreeing to assist with this."

- Check provision of relevant study information sheet
- Confirm who the participants are (and their relationship to each other if in a pair or group)
- Confirm informed consent or consultee declaration

For peer review only - http://bmjopen.bmj.com/site/about/guidelines.xhtml

Get Real with Meeting Centres

1: Questions about who the Meeting Centre is aimed at helping:

- What do you like about attending the Meeting Centre?
- How did you find out about the Meeting Centre?
- What might put people off coming to the Meeting Centre or want to stop coming along?
- What do you think of the activities at the Meeting Centre?
- Is there anything that make it difficult or challenging to attend?
- How do you feel about the cost?

2: Questions about people's roles at the Meeting Centre:

- Do you have a role in choosing what activities take place and deciding things?
- Do you have a role in helping out around the Meeting Centre?
- How involved in the Meeting Centre is your GP, or any care workers, nurses or other health professionals you know?
- Who provides transport to get there?

3: Questions about how a Meeting Centre has developed:

- How has the Meeting Centre changed over time?
- What happened with the Meeting Centre during lockdown and how did you feel about it?

4: Questions about how well things work:

- What do you like most and what is not so good about the Meeting Centre?
- What is most important to the Meeting Centre's success, and what can get in the way?

For peer review only - http://bmjopen.bmj.com/site/about/guidelines.xhtml

Get Real with Meeting Centres

5: Questions about who has the power to influence the success of a Meeting Centre:

- Do you know if authorities like the County Council, the NHS, the Government or any relevant big national charities help or hinder the success of the Meeting Centre?
- Do you feel like you are in control of what happens at the Meeting Centre?

6: Questions about the place and background circumstances around the Meeting Centre:

- Is the venue appropriate and easy to get around?
- Do you know how your GPs, care workers etc. view the Meeting Centre?
- Do you know how the local community views the Meeting Centre?
- How easy is it for you to get to the Meeting Centre and what is transport like?

Interview closure

Is there anything else you'd like to tell us that you feel we should have asked you?

Many thanks for your time.

(Confirm any arrangements for checking accuracy of interpretation of views).

For peer review only - http://bmjopen.bmj.com/site/about/guidelines.xhtml

Interview/Group Discussion Schedule

For external partners & stakeholders to Meeting Centres

Note: Which questions will be asked will be tailored to suit the relationship the external interviewee has with the Meeting Centre – e.g. whether a direct referrer or someone otherwise involved with the local dementia care pathway – to keep the number of questions concise and relevant, appropriate and understandable.

For peer review only

Introductions

Introduction of research staff and explanation of purpose:

"The purpose of this interview is to better understand how Meeting Centres have got up and running and how they have kept going over the past few years. We want to ask people for their knowledge and experience of how their Meeting Centre is run and what has made it a success, so that we can learn from it.

We will use what we learn to develop user-friendly guidance and materials to best help Meeting Centres survive and thrive in the long-term. We will also use this information to help policy makers and government understand what they can do to improve this situation. Our project will support the growth of more Meeting Centres to support people in desperate need. Thank you for agreeing to assist with this."

- Check provision of relevant study information sheet
- Confirm informed consent
- Ask for a description of the participant's role and involvement with the Meeting Centre

For peer review only - http://bmjopen.bmj.com/site/about/guidelines.xhtml

Get Real with Meeting Centres

1: Questions about who the Meeting Centre is aimed at helping:

- What is your understanding of who can become a member and who else benefits?
- Do you have access to Meeting Centre advertising/info materials? Are they effective?
- How does the Meeting Centre reach the right people?
- If you are involved in referring, how do you assess if a Meeting Centre is appropriate for someone? What level of consultation is carried out with people and families?
- What factors do you think most make Meeting Centres attractive to people? What do people value most?
- What could put people off going to a Meeting Centre?
- To what extent do you think the Meeting Centre meets people's needs?
- What are the main benefits or challenges for the people supporting attendees (such as family carers)?

2: Questions about people's roles at the Meeting Centre:

- What role do you see yourself as playing in the Meeting Centre and how it functions?
- How did you get involved? Has your involvement changed over time?
- How important is the Meeting Centre, or initiatives like it, to your work?
- How important do you think your support is to the Meeting Centre?
- Who else (people or organizations) play a role in supporting the Meeting Centre? What do they do and how did they come to be involved?
- Are there any challenges or points of tension in working with the Meeting Centre?

3: Questions about how a Meeting Centre has developed:

- What was most important in helping or hindering the setting up of the Meeting Centre?
- What are the most important things to have in place now, and going forward? Are these the same things, or is the focus different now?
- How has the Meeting Centre and its work changed over time?
- Did you continue to have involvement with the Meeting Centre during lockdown? If so, how?
- How has the pandemic impacted upon the Meeting Centre, now and going forward?

4: Questions about how well things work.

- What do you think is most key to keeping a Meeting Centre going long term? What are the main challenges?
- What is good about how the Meeting Centre is run and its current situation?

IRAS ID: 294636 Version: v0.3 09/06/21

For peer review only

- What is not so good about how the Meeting Centre is run and its current situation?
- What opportunities are there for things to change – what should be done and what can be done?

5: Questions about who has the power to influence the success of a Meeting Centre, including:

- Whose support (person or organization) is essential to making a success of the Meeting Centre?
- Does anyone (person or organization) have the power to stop the Meeting Centre from being able to run?
- What could influence their attitudes and decisions regarding the Meeting Centre?
- How could local authorities help or hinder the success of the Meeting Centre? How could national authorities help?
- Who in the Meeting Centre team could the Meeting Centre not continue without?

6: Questions about the place and background circumstances around the Meeting Centre, including:

- Do you know if the venue is appropriate and working well for everyone?
- How does the local healthcare pathway fit with the Meeting Centre? Does it help, hinder or have no bearing on the Meeting Centre's success? How could things be done differently?
- How does the local/wider community view the Meeting Centre? Does it help, hinder or have no bearing on the Meeting Centre's success?
- How does the geography of the area (e.g. rural or urban) affect the Meeting Centre's success?
- How do people get to the Meeting Centre? What distance do they come from?
- How suitable is public transport? How available is community transport?

Interview closure

Is there anything else you'd like to tell us that you feel we should have asked you?

Many thanks for your time.

(Confirm any arrangements for checking accuracy of interpretation of views).

For peer review only - http://bmjopen.bmj.com/site/about/guidelines.xhtml

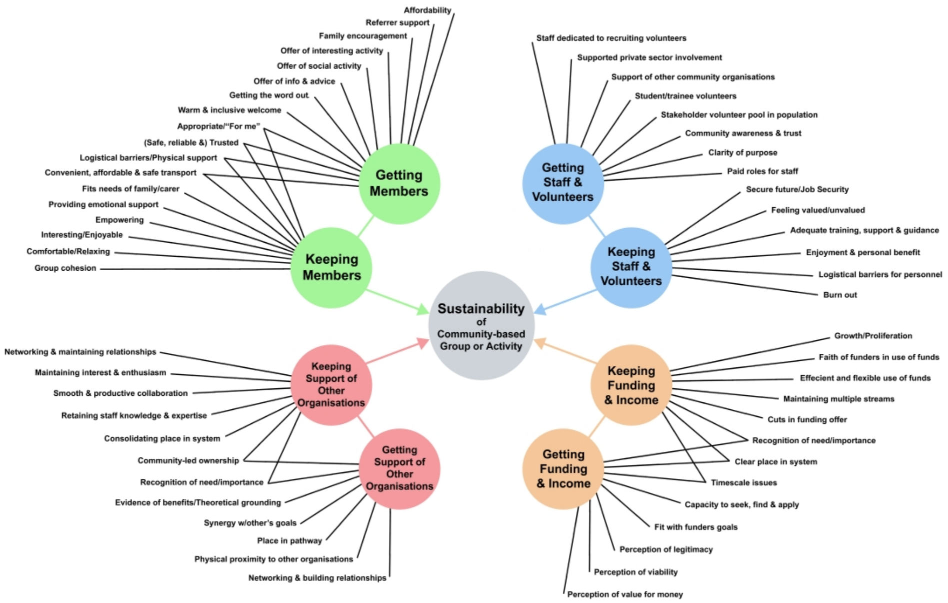

SCI-Dem overview: Factors affecting the sustainability of community-based groups and activities

600x424mm (300 x 300 DPI)

Information Sheet for people involved in Meeting Centres

Research Study Title - Get Real with Meeting Centres: A Realist Evaluation

You have received this information sheet because we would like you to take part in our research study. The following information explains what the research study is and what it would mean for you if you decide to be involved. Please take time to read over it and discuss the information with other people before you make your decision.

What is the purpose of the study?

This study will look at how three Meeting Centres have got up and running and how they have kept going over the past few years. One of these is the Meeting Centre that you have involvement with. Many more Meeting Centres are now starting, so it is important we learn more about what has helped to make Meeting Centres successful. We have developed this study so that we can learn from it and develop tips and guidance for others. We will take all the information people tell us and map out how each Meeting Centre works, how it has worked over the past few years, and how best to plan for the future, going forward.

Why am I being asked to take part?

We are asking you to take part as you have direct experience of being involved in a Meeting Centre. We want to ask people for their knowledge and experience of how their Meeting Centre is run and what has made it a success.

Do I have to take part?

No, you do not have to take part in the study if you do not want to. You will be given a week to decide. If you decide against it, it will have no impact on your involvement with your Meeting Centre. You can change your mind about being involved in the study at any time and without giving a reason. Information about people who have not consented to be involved will not be recorded as part of the study. You also have the right to withdraw your data after participation, in which case it will be destroyed on request.

For peer review only - http://bmjopen.bmj.com/site/about/guidelines.xhtml
For peer review only

If you decide that you would like to take part in this research study, you will first be asked to sign a consent form and then we will arrange with you the next steps of taking part.

What will happen if I wish to take part?

You will have the opportunity to be involved in informal interviews and group discussions over the coming months. You can make the decision on how much involvement you would like to have and which aspects you would like to get involved with.

The research study and COVID

We hope to be able to hold interviews and group discussions in person, but this is dependent on the COVID-19 restrictions. We are hopeful that in the near future it will be safe for us to visit each Meeting Centre. If so, we will abide by all current local and national guidance on what is safe to do when visiting. If this is not possible, then we will carry out our interviews and discussion groups by online meetings (such as via Zoom) or by telephone and email.

Even if we can attend Meeting Centres, you can let us know whether you would like to be involved face-to-face or via an online method such as Zoom.

Will my involvement be kept confidential?

We keep the specific feedback in individual interviews confidential, but we will state our general findings in our final report. However, if something is disclosed during interviews or discussions that could impact upon the safety of yourself or others, confidentially may be breached if necessary to ensure safeguarding. Any feedback that we get from you will be checked for accuracy and anonymised through the removal of people's names and other personal information. When we write up our findings, we will use quotes from people we have spoken with. You will be asked your preferences on whether or not you would like your name to be referenced by the quote. When the results of the study are presented, we will not use the name of the Meeting Centres involved in the study but will refer to them by region.

For peer review only - http://bmjopen.bmj.com/site/about/guidelines.xhtml

What are the possible disadvantages to taking part?

There are no obvious disadvantages to you taking part in the research. We are interested to hear your feedback.

What are the possible benefits of taking part?

By taking part in this study you will be providing valuable information that we can learn from, to help other Meeting Centres to be a success. You get to share your opinions on Meeting Centres, what you think of them and how they might improve to help those who attend them. You may also help your own Meeting Centre, by helping us to understand how it works and what else we can do to help it in the future.

We hope that you will find taking part in interviews and discussions with us interesting and enjoyable.

What will happen to the results of the study?

The results will give us the information to make recommendations to other Meeting Centres across the UK. The results of the study will be written up in a final report and shared with the National Institute of Health Research, who are funding the study. The results will also be used to write articles, reports or include in presentations. We will make sure our results are made available to share with you once the study is complete.

Who is organising or funding the study?

This study is being led by the Association for Dementia Studies at the University of Worcester. It is funded by the **National Institute for Health Research (NIHR)**

Who has reviewed the study?

This research study has been reviewed and approved by XXX. A research ethics committee is a group of people who review research studies to ensure they protect the dignity, rights, safety and well-being of research participants and researchers.

For peer review only - http://bmjopen.bmj.com/site/about/guidelines.xhtml

For any further information please contact:

Shirley Evans – PI

s.evans@worc.ac.uk

Association for Dementia Studies

University of Worcester

Henwick Grove, Worcester, WR2 6AJ

Thomas Morton – Research Associate

t.morton@worc.ac.uk

Association for Dementia Studies

University of Worcester

Henwick Grove, Worcester, WR2 6AJ

Thank you for taking time to read this information sheet.

For peer review only - http://bmjopen.bmj.com/site/about/guidelines.xhtml

Information Sheet (short version)

The Get Real with Meeting Centres Study

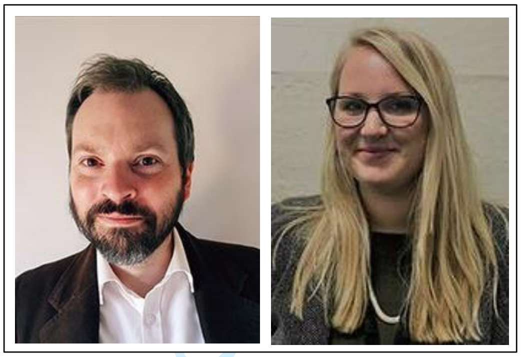

The Researchers:
Thomas (left) and Faith (Right)

What we are doing

We are doing a research study at the Meeting Centre that you go to because we want to find out more about Meeting Centres and what makes them successful. Meeting Centres are really popular and there are lots of new ones that are being set up. We hope that we can find out how to make them the best that they can be.

We would like you to talk to us about the Meeting Centre that you go to and your feelings about it.

How you can take part

Tell someone at your Meeting Centre if you would like to take part. You will be invited to take part in things like **group discussions** and **one-to-one conversations,** to talk to us about the Meeting Centre you go to.

Group Discussions

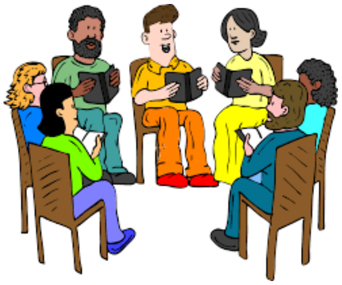

One-to-one Conversations

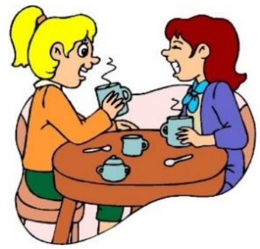

For peer review only - http://bmjopen.bmj.com/site/about/guidelines.xhtml

What will happen next?

We will ask you to sign a consent form saying that you want to take part. We will then arrange the next steps, like when you would like to talk with us and how.

Will my information be confidential?

The information you tell us will be checked for accuracy and anonymised by removing anyone's names and any other personal information. If we quote something you have said, we will ask you if you wish for your name to be used before we share it with anyone. Confidentiality will only be broken if there is a danger to the safety of yourself or someone else.

The Meeting Centre you go to will not be named in any reports, but we will describe it by the region it is located in.

What if I have a concern?

If you have any worries or questions please talk to us or someone else that you feel comfortable with. We are happy to arrange a time to come and talk with you and answer any questions. **For more info, contact:**

Thomas Morton

University of Worcester, Henwick Grove, Worcester, WR2 6AJ

Tel: 01905 542326 **Email: t.morton@worc.ac.uk**

Please remember:

You do not have to take part in this research study. It is your decision if you want to stop taking part in the research at any point and you do not have to give a reason why. If you do not decide to take part but then change your mind, that is fine as well. You can join in when you wish.

Whatever you decide, it will not affect your involvement with the Meeting Centre you go to.

This research has been approved by XXX

Thank you for taking time to read this information sheet.

For peer review only - http://bmjopen.bmj.com/site/about/guidelines.xhtml

Information Sheet for external partners working with and supporting Meeting Centres

External partners may include health, social care and third sector professionals, community-based partners and those involved with the local dementia pathway/DFC

Research Study Title - Get Real with Meeting Centres: A Realist Evaluation

You have received this information sheet because we would like you to take part in our research study. The following information explains what the research study is and what it would mean for you if you decide to get involved. Please take time to read over it and discuss the information.

What is the purpose of the study?

This study will look at how three Meeting Centres have got up and running and how they have kept going over the past few years. One of these is the Meeting Centre that you work with/ support. Many more Meeting Centres are now starting, so it is important we learn more about what has helped to make Meeting Centres successful. We have developed this study so that we can learn from it and develop tips and guidance for others. We will take all the information people tell us and map out how each Meeting Centre works, how it has worked over the past few years, and how best to plan for the future, going forward.

Why am I being asked to take part?

We are asking you to take part as you have direct experience of working with/supporting a Meeting Centre. We want to ask people for their knowledge and experience of how they think the Meeting Centre is run and what has made it a success.

Do I have to take part?

No, you do not have to take part in the study. You will be given a week to decide. If you decide against it, it will have no impact on your relationship with the Meeting Centre you currently work with/support. You can change your mind at any time and without giving a reason.

For peer review only - http://bmjopen.bmj.com/site/about/guidelines.xhtml

Information about people who have not consented to be involved will not be recorded as part of the study. You also have the right to withdraw your data after participation by which it will be destroyed on request.

If you decide that you would like to take part in this research study, you will first be asked to sign a consent form. We will then discuss with you the next steps of taking part.

What will happen if I wish to take part?

You will have the opportunity to be involved in informal interviews and group discussions over the coming months. You can make the decision on how much involvement you would like to have and which aspects you would like to get involved with.

The research study and COVID

We hope to be able to hold interviews and group discussions in person, but this is dependent on the COVID-19 restrictions. We are hopeful that in the near future it will be safe for us to visit each Meeting Centre. If so, we will abide by all current local and national guidance on what is safe to do when visiting. If this is not possible, then we will carry out our interviews and discussion group by online meetings (such as via Zoom) or by telephone and email.

Even if we can attend Meeting Centres, you can let us know whether you would like to be involved face-to-face or via an online method such as Zoom.

Will my involvement be kept confidential?

We keep the specific feedback in individual interviews confidential, but we will state our general findings in our final report. However, if something is disclosed during interviews or discussions that could impact upon the safety of yourself or others, confidentially may be breached if necessary to ensure safeguarding. Any feedback that we get from you will be checked for accuracy and anonymised through the removal of people's names and other personal information. When we write up our findings, we will use quotes from people we have spoken with. You will be asked your preferences on whether or not you would like your name

For peer review only - http://bmjopen.bmj.com/site/about/guidelines.xhtml
For peer review only

to be referenced by the quote. When the results of the study are presented, we will not use the name of the Meeting Centres involved in the study but will refer to them by region.

What are the possible disadvantages to taking part?

There are no obvious disadvantages to you taking part in the research. We are interested to hear your feedback.

What are the possible benefits of taking part?

By taking part in this study you will be providing valuable information that we can learn from to help other Meeting Centres to be a success. You get to share your opinions on Meeting Centres, what you think of them and how they might improve to help those who attend them. You may also help the Meeting Centre you work with/support, by helping us to understand how it works and what else we can do to help it in the future.

What will happen to the results of the study?

The results will give us the information to make recommendations to other Meeting Centres across the UK. They will be written up in a final report and shared with the National Institute of Health Research, who are funding the study. The results will also be used to write articles, reports or be included in presentations. We will make sure our results are made available to share with you once the study is complete.

Who is organising or funding the study?

This study is being led by the Association for Dementia Studies at the University of Worcester. It is funded by the **National Institute for Health Research (NIHR)**

Who has reviewed the study?

This research study has been reviewed and approved by XXX. A research ethics committee is a group of people who review research studies to ensure they protect the dignity, rights, safety and well-being of research participants and researchers.

For peer review only - http://bmjopen.bmj.com/site/about/guidelines.xhtml

For any further information please contact:

Shirley Evans – PI

s.evans@worc.ac.uk

Association for Dementia Studies

University of Worcester

Henwick Grove, Worcester, WR2 6AJ

Thomas Morton – Research Associate

t.morton@worc.ac.uk

Association for Dementia Studies

University of Worcester

Henwick Grove, Worcester, WR2 6AJ

Thank you for taking time to read this information sheet.

For peer review only - http://bmjopen.bmj.com/site/about/guidelines.xhtml

Information Sheet for Personal Consultees

A personal consultee is someone who advises on the preferences and past wishes of a person who is no longer able to give informed consent.

Research Study Title - Get Real with Meeting Centres: A Realist Evaluation

You have received this information sheet because we are inviting your relative/friend/the person you represent to take part in our research study. The following information explains what the research study is and what it would mean for the person you are acting as a personal consultee on behalf of to get involved. Please take time to read over it and discuss the information with other people before you make your decision.

What is the purpose of the study?

This study will look at how three Meeting Centres have got up and running and how they have kept going over the past few years. One of these is the Meeting Centre that your relative/friend /the person you represent goes to. Many more Meeting Centres are now starting, so it is important we learn more about what has helped to make Meeting Centres successful. We have developed this study so that we can learn from it and develop tips and guidance for others. We will take all the information people tell us and map out how each Meeting Centre works, how it has worked over the past few years, and how best to plan for the future, going forward.

Why am I being asked about this study?

We would like to involve your relative/friend/the person you represent in this study. Due to the fact that your relative/friend/the person you represent may be unable to decide for him/ herself, we are asking you to inform us on their behalf. We are asking them to take part as they have direct experience of going to a Meeting Centre. We want to ask people for their knowledge and experience of how they think the Meeting Centre is run and what has made it a success.

For peer review only - http://bmjopen.bmj.com/site/about/guidelines.xhtml

Does my relative/friend/the person I represent have to take part?

No. We are asking for your advice about whether they might object to taking part. You will be given a week to decide. They do not have to take part and their experience of going to their Meeting Centre will not change if they do not take part.

You may want to think about whether they have expressed an interest in taking part in research before. If you think they would **not object** to taking part, then please complete the **Consultee Declaration Form** and return it to the researchers (or a member of Meeting Centre staff to pass on to them).

If you feel that the person would not like to take part, it will have no impact on yours or their relationship with the Meeting Centre that they currently go to. You can change your mind at any time, without giving a reason. Information about people who have not consented to be involved will not be recorded as part of the study. You also have the right to withdraw your data after participation, in which case it will be destroyed on request.

If you think your relative/friend/the person you represent would like to take part in this research, we will arrange the next steps of taking part with both of you.

What will happen if I advise they would wish to take part?

If you say yes to your relative/friend/the person you represent taking part the following things will happen:

1. After reading this information sheet, you will need to complete a consultee declaration form as the person's consultee.
2. A member of the research team will then explain the study to you and your relative/ friend/the person you represent and invite them to take part in participating in an informal interview or a group discussion around the Meeting Centre that they go to.
3. You and your relative/friend/the person you represent can make the decision on how much involvement they would like to have and which aspects they would like to get involved with.

For peer review only - http://bmjopen.bmj.com/site/about/guidelines.xhtml

4. The researcher will take some notes of your relative/friend/the person you represent's feedback.
5. The research team will then look at this feedback and integrate it into their findings.

The research study and COVID

We hope to be able to hold interviews and group discussions in person, but this is dependent on the COVID-19 restrictions. We are hopeful that in the near future it will be safe for us to visit each Meeting Centre. If so, we will abide by all current local and national guidance on what is safe to do when visiting. If this is not possible, then we will carry out our interviews and discussion group by online meetings (such as via Zoom) or by telephone and email.

Even if we can attend Meeting Centres, you can let us know whether you would like to be involved face-to-face or via an online method such as Zoom.

Will the involvement of the person I represent be kept confidential?

We keep the specific feedback in individual interviews confidential, but we will state our general findings in our final report. However, if something is disclosed during interviews or discussions that could impact upon the safety of your relative/friend/the person you represent or others, confidentially may be breached if necessary to ensure safeguarding. Any feedback that we get from you will be checked for accuracy and anonymised through the removal of people's names and other personal information. When we write up our findings, we will use quotes from people we have spoken with, but these will be attributed to a pseudonym to retain anonymity in the case of people who are unable to give informed consent. When the results of the study are presented, we will not use the name of the Meeting Centres involved in the study but will refer to them by region.

What are the possible disadvantages to taking part?

There are no obvious disadvantages to your relative/friend/the person you represent taking part in the research. We are interested to hear their feedback.

For peer review only - http://bmjopen.bmj.com/site/about/guidelines.xhtml

What are the possible benefits of taking part?

By taking part in this study you will be providing valuable information that we can learn from to help other Meeting Centres to be a success. Your relative/friend/the person you represent will be able to have their say on Meeting Centres, what they think of them and how they might improve to help those who attend them. By being involved, they may also help the Meeting Centre that they go to by helping us to understand how it works and what else we can do to help it in the future.

What will happen to the results of the study?

The results will give us the information to make recommendations to other Meeting Centres across the UK. The results of the study will be written up in a final report and shared with the National Institute of Health Research, who are funding the study. The results will also be used to write articles, reports or be included in presentations. We will make sure our results are made available to share with you once the study is complete.

Who is organising or funding the study?

This study is being led by the Association for Dementia Studies at the University of Worcester. It is funded by the **National Institute for Health Research (NIHR)**

Who has reviewed the study?

This research study has been reviewed and approved by XXX. A research ethics committee is a group of people who review research studies to ensure they protect the dignity, rights, safety and well-being of research participants and researchers.

For peer review only - http://bmjopen.bmj.com/site/about/guidelines.xhtml

For any further information please contact:

Shirley Evans – PI

s.evans@worc.ac.uk

Association for Dementia Studies

University of Worcester

Henwick Grove, Worcester, WR2 6AJ

Thomas Morton – Research Associate

t.morton@worc.ac.uk

Association for Dementia Studies

University of Worcester

Henwick Grove, Worcester, WR2 6AJ

Thank you for taking time to read this information sheet.

For peer review only

For peer review only - http://bmjopen.bmj.com/site/about/guidelines.xhtml

IRAS ID: 294636

Centre Number:

Study Number:

Participant Identification Number:

University of Worcester
Association for Dementia Studies

Research Study Consent Form

Title of Project: Get Real with Meeting Centres: A Realist Evaluation

Name of Researcher:

Please initial box

1. I confirm that I have read the information sheet dated 09/06/21 (version 0.4) for the above study. I have had the opportunity to consider the information, ask questions and have had these answered satisfactorily. ☐

2. I understand that my participation is voluntary and that I am free to withdraw at any time without giving any reason, without my involvement in the Meeting Centre or legal rights being affected. ☐

3. I understand that the information collected about me will be used to support other research in the future and may be shared anonymously with other researchers. ☐

4. I understand that an audio recording may be made of interviews and/or discussions that I take part in as part of this research, and do not object to this. ☐

5. I understand that quotes from what I say in my interviews and/or discussions may be used when the findings of this study are written up. ☐

   With regards to the above, please tick one of the below:

   I would like my name to be changed on any quotes used from me. ☐

   I am happy my name to be used on any quotes from me. ☐

Please turn over…

For peer review only - http://bmjopen.bmj.com/site/about/guidelines.xhtml

6. I agree to take part in the above study. ☐

7. I know who to contact if I have any concerns about this research. ☐

Name of participant:	Date:	Signature:
____________________	________________	________________

Name of person taking consent:	Date:	Signature:
____________________	________________	________________

For peer review only

For peer review only - http://bmjopen.bmj.com/site/about/guidelines.xhtml

IRAS ID: 294636

Centre Number:

Study Number:

Participant Identification Number for this study:

University of Worcester
Association for Dementia Studies

Consultee Declaration Form

Title of Project: Get Real with Meeting Centres: A Realist Evaluation

Name of Researcher:

Please initial box

1. I ……………………..….. have been consulted about ……………………….….’s participation in this research project. I have had the opportunity to ask questions about the study and understand what is involved. ☐

2. I understand that I do not have to act as consultee and I can change my mind at any time. ☐

3. In my opinion he/she would have no objection to taking part in the above study. ☐

4. I understand I can request he/she is withdrawn from the study at any time, without giving a reason and without his/her involvement in the Meeting Centre being affected. ☐

5. I understand that the information collected about him/her will be used to support other research in the future, and may be shared anonymously with other researchers. ☐

6. I understand that an audio recording may be made of interviews and/or discussions that he/she takes part in as part of this research, and in my opinion he/she would not object to this. ☐

7. I understand that quotes from what he/she says in interviews and/or discussions may be used when the findings of this study are written up, with names changed to preserve anonymity, and in my opinion he/she would not object to this. ☐

Please turn over…

For peer review only

For peer review only - http://bmjopen.bmj.com/site/about/guidelines.xhtml

8. I know who to contact if I have any concerns about this research. ☐

Name of Consultee: Date: Signature:

Relationship to participant:

Person undertaking consultation:

Name Date Signature

To be completed by researcher prior to seeking consultee declaration (tick one),

☐ 1. Potential participant showed signs of a positive or interested attitude when going through the participant information

☐ 2. Potential participant showed signs of a negative or anxious attitude toward the research or taking part in it when going through the information

For peer review only - http://bmjopen.bmj.com/site/about/guidelines.xhtml
For peer review only

RAMESES II reporting standards for realist evaluations: checklist

Title: The sustainability of locally-driven centres for those affected by dementia: A protocol for the Get Real with Meeting Centres realist evaluation

TITLE			Reported in document Y/N/Unclear/ Not applicable	Page(s) in document	Comment
1		In the title, identify the document as a realist evaluation.	Y	1,2	
SUMMARY OR ABSTRACT					
2		Journal articles will usually require an abstract, while reports and other forms of publication will usually benefit from a short summary. The abstract or summary should include brief details on: the policy, programme or initiative under evaluation; programme setting; purpose of the evaluation; evaluation question(s) and/or objective(s); evaluation strategy; data collection, documentation and analysis methods; key findings and conclusions.<br><br>Where journals require it and the nature of the study is appropriate, brief details of respondents to the evaluation and recruitment and sampling processes may also be included.<br><br>Sufficient detail should be provided to identify that a realist approach was used and that realist programme theory was developed and/or refined.	Y	2-3	Abstract presented in study protocol format

For peer review only

**INTRODUCTION**					
**3**	Rationale for evaluation	Explain the purpose of the evaluation and the implications for its focus and design.	Y	4-6	
**4**	Programme theory	Describe the initial programme theory (or theories) that underpin the programme, policy or initiative.	Y	4, 6, 10, 11. Supplementary file 2.	As this is a study protocol these will be developed, however programme theory from the previous SCI-Dem study will act as a preliminary framework, referenced in the Introduction (page 4) and Methods (pages 6 and 10) sections and described in Supplementary file 2. MCs are also guided by the Adaptation Coping Model, referenced on pages 4 and 11.
**5**	Evaluation questions, objectives and focus	State the evaluation question(s) and specify the objectives for the evaluation. Describe whether and how the programme theory was used to define the scope and focus of the evaluation.	Y	6-7	
**6**	Ethical approval	State whether the realist evaluation required and has gained ethical approval from the relevant authorities, providing details as appropriate. If ethical approval was deemed unnecessary, explain why.	Y	13	As this is a study protocol, this is dealt with in the Ethics and Dissemination section.
**METHODS**					

For peer review only

**7**	Rationale for using realist evaluation	Explain why a realist evaluation approach was chosen and (if relevant) adapted.	Y	8	
**8**	Environment surrounding the evaluation	Describe the environment in which the evaluation took place.	Y	9	
**9**	Describe the programme policy, initiative or product evaluated	Provide relevant details on the programme, policy or initiative evaluated.	Y	5,7,9	
**10**	Describe and justify the evaluation design	A description and justification of the evaluation design (i.e. the account of what was planned, done and why) should be included, at least in summary form or as an appendix, in the document which presents the main findings. If this is not done, the omission should be justified and a reference or link to the evaluation design given. It may also be useful to publish or make freely available (e.g. online on a website) any original evaluation design document or protocol, where they exist.	Y	7-13	
**11**	Data collection methods	Describe and justify the data collection methods – which ones were used, why and how they fed into developing, supporting, refuting or refining programme theory.<br><br>Provide details of the steps taken to enhance the trustworthiness of data collection and documentation.	Y	9-13	

For peer review only

**12**	Recruitment process and sampling strategy	Describe how respondents to the evaluation were recruited or engaged and how the sample contributed to the development, support, refutation or refinement of programme theory.	Y	7-15	Recruitment and sampling is covered both in the Methods, and Ethics and Dissemination sections.
**13**	Data analysis	Describe in detail how data were analysed. This section should include information on the constructs that were identified, the process of analysis, how the programme theory was further developed, supported, refuted and refined, and (where relevant) how analysis changed as the evaluation unfolded.	Y	10-13	As this is a study protocol, this covers plans for data analysis.
**RESULTS**					
**14**	Details of participants	Report (if applicable) who took part in the evaluation, the details of the data they provided and how the data was used to develop, support, refute or refine programme theory.	Not applicable		Study protocol
**15**	Main findings	Present the key findings, linking them to contexts, mechanisms and outcome configurations. Show how they were used to further develop, test or refine the programme theory.	Not applicable		Study protocol
**DISCUSSION**					
**16**	Summary of findings	Summarise the main findings with attention to the evaluation questions, purpose of the evaluation, programme theory and intended audience.	Not applicable		Study protocol

For peer review only

**17**	Strengths, limitations and future directions	Discuss both the strengths of the evaluation and its limitations. These should include (but need not be limited to): (1) consideration of all the steps in the evaluation processes; and (2) comment on the adequacy, trustworthiness and value of the explanatory insights which emerged.<br><br>In many evaluations, there will be an expectation to provide guidance on future directions for the programme, policy or initiative, its implementation and/or design. The particular implications arising from the realist nature of the findings should be reflected in these discussions.	Not applicable		Study protocol
**18**	Comparison with existing literature	Where appropriate, compare and contrast the evaluation's findings with the existing literature on similar programmes, policies or initiatives.	Not applicable		Study protocol
**19**	Conclusion and recommendations	List the main conclusions that are justified by the analyses of the data. If appropriate, offer recommendations consistent with a realist approach.	Not applicable		Study protocol
**20**	Funding and conflict of interest	State the funding source (if any) for the evaluation, the role played by the funder (if any) and any conflicts of interests of the evaluators.	Y	13,18	

Adapted from table 1 in: Wong G, Westhorp G, Manzano A, et al. RAMESES II reporting standards for realist evaluations. BMC Med 2016;14:96

For peer review only
