# Supplementary material for: Sustainability of locally driven centres for those affected by dementia: a protocol for the get real with meeting centres realist evaluation
Source: BMJ Open. 2022 May 2;12(5):e062697. doi: 10.1136/bmjopen-2022-062697 (PMC9062872; doi:10.1136/bmjopen-2022-062697)
Supplement: Reviewer comments [file bmjopen-2022-062697.reviewer_comments.pdf]

PEER REVIEW HISTORY

BMJ Open publishes all reviews undertaken for accepted manuscripts. Reviewers are asked to complete a checklist review form (**http://bmjopen.bmj.com/site/about/resources/checklist.pdf**) and are provided with free text boxes to elaborate on their assessment. These free text comments are reproduced below.

ARTICLE DETAILS

**TITLE (PROVISIONAL)**	The sustainability of locally-driven centres for those affected by dementia: A protocol for the Get Real with Meeting Centres realist evaluation
**AUTHORS**	Morton, Thomas; Evans, Shirley; Brooker, Dawn; Williamson, Tracey; Wong, Geoff; Tinelli, M; Frost, Faith; Bray, Jennifer; Hullah, Nigel

This article was not externally reviewed at BMJ Open. Protocols that have been independently assessed prior to submission to BMJ Open are usually fast-tracked to publication on the grounds that further substantial changes will not be possible. This independent assessment will usually be external, independent review for both a substantial grant award from a non-commercial or government funder and ethics approval.
